# Metabarcoding reveals that a non-nutritive sweetener and sucrose yield similar gut microbiota patterns in Wistar rats

**DOI:** 10.1590/1678-4685-GMB-2019-0028

**Published:** 2020-03-16

**Authors:** Tiago Falcon, Kelly Carraro Foletto, Marina Siebert, Denise Entrudo Pinto, Michael Andrades, Marcello Casaccia Bertoluci

**Affiliations:** 1 Hospital de Clínicas de Porto Alegre (HCPA), Centro de Pesquisa Experimental, Núcleo de Bioinformática, Porto Alegre, RS, Brazil.; 2 Universidade Federal do Rio Grande do Sul (UFRGS), Programa de Pós-Graduação em Medicina: Ciências Médicas, Porto Alegre, RS, Brazil.; 3 Hospital de Clínicas de Porto Alegre (HCPA), Centro de Pesquisa Experimental, Unidade de Pesquisa Laboratorial, Porto Alegre, RS, Brazil.; 4 Hospital de Clínicas de Porto Alegre (HCPA), Serviço de Endocrinologia, Porto Alegre, RS, Brazil.

**Keywords:** *16S* rDNA, deep sequencing, saccharin, cyclamate, yogurt

## Abstract

The effects of non-nutritive sweeteners (NNS) on the gut microbiota are an area of increasing research interest due to their potential influence on weight gain, insulin resistance, and inflammation. Studies have shown that mice and rats fed saccharin develop weight gain and metabolic alterations, possibly related to changes in gut microbiota. Here, we hypothesized that chronic exposure to a commercial NNS would change the gut microbiota composition in Wistar rats when compared to sucrose exposure. To test this hypothesis, Wistar rats were fed either NNS- or sucrose-supplemented yogurt for 17 weeks alongside standard chow (*ad libitum*). The gut microbiome was assessed by *16S* rDNA deep sequencing. Assembly and quantification were conducted using the Brazilian Microbiome Project pipeline for Ion Torrent data with modifications. Statistical analyses were performed in the R software environment. We found that chronic feeding of a commercial NNS-sweetened yogurt to Wistar rats, within the recommended dose range, did not significantly modify gut microbiota composition in comparison to sucrose-sweetened yogurt. Our findings do not support the hypothesis that moderate exposure to NNS is associated with changes in gut microbiota pattern compared to sucrose, at least in this experimental model.

## Introduction

Non-nutritive sweeteners (NNS) impart a sweet taste to foodstuffs without adding caloric value, and are thus widely used in the management of overweight, glucose intolerance, and other metabolic diseases (Fitch *et al.*, 2012). This class of molecules was first considered inert, but chronic consumption has raised concerns regarding user health and environmental safety (Pepino, 2015; Praveena *et al.*, 2018). Experimental studies suggest that NNS are not as inert as initially thought; dopaminergic degeneration (Amin *et al.*, 2018), changes in the gut microbiota (Suez *et al.*, 2014; Bian et al., 2017a; Uebanso et al., 2017), and cell barrier disruption in vitro (Santos *et al.*, 2018) have been described as consequences of their chronic intake.

Epidemiological studies have shown that NNS intake is paradoxically associated with increased weight gain and adiposity, the metabolic syndrome, type 2 diabetes mellitus, and cardiovascular disease (Colditz *et al.*, 1990; Dhingra *et al.*, 2007; Fowler *et al.*, 2008; Lutsey *et al.*, 2008). In previous experiments, we found that rats fed an isocaloric diet supplemented with commercial NNS gained more weight than rats fed a sucrose-supplemented diet (Feijó *et al.*, 2013; Pinto *et al.*, 2017) or a non-sweetened diet (Foletto *et al.*, 2016). Some mechanisms have been proposed to explain the metabolic alterations seen after NNS use, such as changes in energy expenditure rate (Pinto *et al.*, 2017), interaction with sweet receptors (with consequences for insulin secretion), and changes in the gastrointestinal environment (Pepino, 2015). Emerging evidence has shown that gut microbiota composition is associated with metabolic diseases (Akbari and Hendijani, 2016; Boulangé et al., 2016). Furthermore, the balance between resident microorganisms may predispose the host to weight gain by facilitating recovery of nutrients from food (Turnbaugh *et al.*, 2006)**.** This phenomenon has been demonstrated by fecal transplantation from obese humans to lean mice, which eventually acquired the phenotype of the donors (Goodrich *et al.*, 2014). Some studies have shown that the gut microbiome is disturbed by chronic exposure to several NNS, including acesulfame potassium, aspartame, cyclamate, neotame, saccharin, sucralose, and steviol glycosides (Lobach *et al.*, 2018).

Although these studies are important, results still need to be confirmed for different NNS types, doses, and degrees of exposure. In the present study, we hypothesized that chronic intake of a commercially available NNS (0.3% sodium saccharin and sodium cyclamate, Zero-Cal) would induce changes in the rat gut microbiome.

## Materials and Methods

### Study design and animals

The present study is a reanalysis of previous research; details are given elsewhere (Pinto *et al.*, 2017). In brief, adult male Wistar rats weighing 210 ± 6 grams (mean ± SE) were ranked according to the baseline weight and assigned to receive NNS-supplemented yogurt (NNS, n = 10) or sucrose-sweetened yogurt (SUC, n = 9) in" a block randomization approach.

All the animals received standard chow and water *ad libitum* and were kept individually in translucent polypropylene cages with controlled humidity (65-70%), temperature (22 ± 1°C), and light-dark cycle (12/12 hours).

After 17 weeks, the animals were euthanized by decapitation, the abdomen was opened, and the distal portion of the large intestine was removed. The fecal pellets were moved directly to a sterile cryotube which was immediately frozen in liquid nitrogen and stored in a freezer at -80 °C.

Trained researchers were blinded to group allocation. All the procedures were reviewed and approved by the local institutional animal care and use committee (Comissão de Ética no Uso de Animais em Pesquisa do Hospital de Clínicas de Porto Alegre, protocol number 16-0011) and conducted in accordance with Brazilian law. This manuscript was written in accordance with the ARRIVE guidelines (Kilkenny *et al.*, 2010).

### Dietary manipulation

All rats received standard chow pellets (2.93 kcal/g) (Nuvital CR-1, Nuvilab) *ad libitum*. Chow was added to the top of the cages every 24 hours as needed, and remaining chow was weighed and recorded once a week with an electronic precision scale (AS 5500, Marte, SP, Brazil). Intake was thus calculated and recorded weekly. The largest solid pellets were allocated in the grid feeders. A bottom crumb collector on the outside of the cage was installed to minimize losses. Cages were carefully monitored for any evidence of chow spillage and crumbs were considered for the control of chow intake.

Sweetened yogurt supplements were prepared according to an established protocol, described elsewhere (Feijó *et al.*, 2013; Foletto *et al.*, 2016; Pinto *et al.*, 2017). Briefly, 20 mL of standardized low-fat yogurt (Nestlé, SP, Brazil) was supplemented with either 20% sucrose (União, SP, Brazil) or a commercial NNS (0.3% sodium saccharin and sodium cyclamate, Zero-Cal, SP, Brazil). Additionally, 15 mL of pure water was added into yogurt to dilute and adjust viscosity to allow easier drinking, yielding a solution of 11.4% sucrose or 0.17% NNS. Yogurt supplements were offered for 22 hours each day (from 11 AM to 9 AM), 7 days a week, throughout the experiment. Yogurt was offered in special bottles with adapted nozzles to avoid leakage. The caloric densities of sucrose- and NNS-supplemented yogurt were 0.63 kcal/mL (~170 kcal/wk) and 0.24 kcal/mL (~60 kcal/wk), respectively. All rats ingested more than 65% of the yogurt supplement offered. The yogurt bottles were also checked for any sign of leakage or clogging. Water bottles were changed every 2 or 3 days and leftover water volumes were recorded. No preference for any beverage was noted during the study.

### DNA extraction and sequencing of the *16S* gene

Bacterial DNA was extracted from fecal pellets using the QIAmp DNA Stool Mini Kit (Qiagen) and then quantitated using the Qubit dsDNA HS assay kit (Thermo Fisher Scientific) in a Qubit fluorimeter (Invitrogen).

Approximately 50 ng of DNA was used for amplification of the V4 hypervariable region of the bacterial *16S* rRNA encoding gene by the polymerase chain reaction (PCR). The resulting product was purified and used in the preparation of the emulsion PCR followed by the sequencing reaction in an Ion Torrent Personal Genome Machine (PGM) System (Life Technologies). Sequencing data were processed in QIIME software. Bacterial diversity analyses were based on the degree of similarity between *16S* rDNA sequences, which were grouped into Operational Taxonomic Units (OTUs).

### Sequencing data analysis

We followed the Brazilian Microbiome Project (BMP) *16S* profiling analysis pipeline for Ion Torrent (Pylro *et al.*, 2014) with a few modifications. The BMP pipeline uses a mix of the UPARSE (Edgar, 2013) and QIIME (v 1.9.1) (Caporaso *et al.*, 2010) pipelines. UPARSE is used for chimera removal and *de novo* OTU picking and quantification, while QIIME is used to map and classify the OTUs based on the Greengenes database (13_8), generate the output table, and perform alpha and beta diversity analyses. Using QIIME^TM^, we changed the standard classifier from uclust to rdp. The rdp classifier (Wang *et al.*, 2007) in QIIME^TM^ uses the Greengenes database sequences to train the classifier and thus perform OTU classification. Another difference was that the BMP pipeline suggests the QIIME^TM^ default of 0.5 as the least confidence to record an assignment; here, we used a least confidence of 0.8.

### Statistical analysis

All data were normalized using Aitchison’s log-ratio (Aitchison, 1982, 1986), as we are working with compositional data (Gloor *et al.*, 2017). The R software (v 3.4.4) (R Core Team, 2018), package vegan (v. 2.4) (Oksanen *et al.*, 2017), was used to perform the diversity analyses (alpha and beta diversities, Shannon and Simpson diversity indices, and richness comparisons) and their respective plots. The Shapiro-Wilk test of normality was applied to all identified taxa levels, except those unclassified (Table S1). Then, Student’s *t*-test or the Wilcoxon rank-sum test were used in accordance with the value distribution (*t*-test for parametric and Wilcoxon for non-parametric data). All p-values were corrected using the false discovery rate (FDR) method. Only those taxa comparisons with FDR < 0.05 were considered as a statistically significant difference between groups. Unclassified taxa were used only for the diversity analyses.

## Results

Yogurt intake was similar in the NNS- and SUC-supplemented groups over the 17-week study period. NNS intake in the NNS group was stable over time (median = 154 μL/kg/day; IQR = 39 μL/kg/day) (Figure S1).

**Figure 1:**
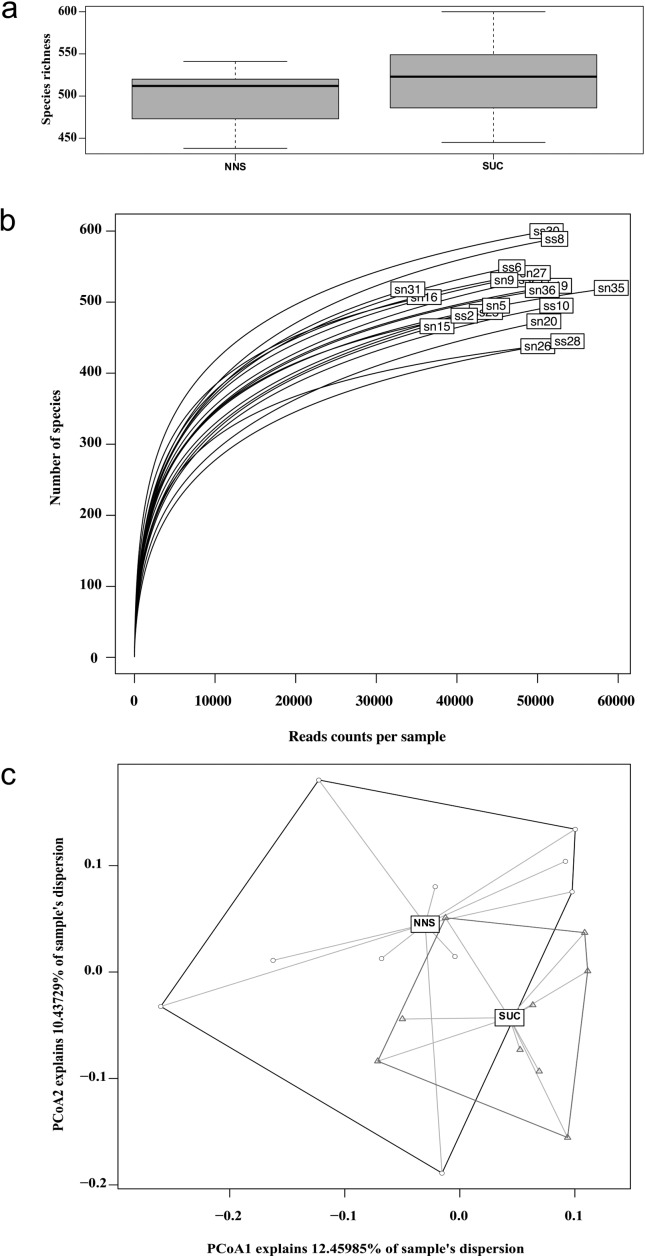
Diversity analysis plots. (a) Species richness boxplot. The dark line inside the boxplots is the median, separating the upper quantile. Error bars represent the standard deviation. (b) Rarefaction plot per sample. sn, samples belonging to the NNS group; ss, samples belonging to the SUC group. Numbers indicate the number of the rat in the box for internal annotation. (c) Weighted beta diversity plot using principal coordinate analysis. Black circles represent the NNS samples; red triangles represent the SUC samples. Dark lines connect the most distant samples from the centroid of the NNS group, while red lines connect the most distant samples from the centroid of the SUC group. NNS, non-nutritive sweetener (n = 10); SUC, sucrose (n = 9).

**Figure 2:**
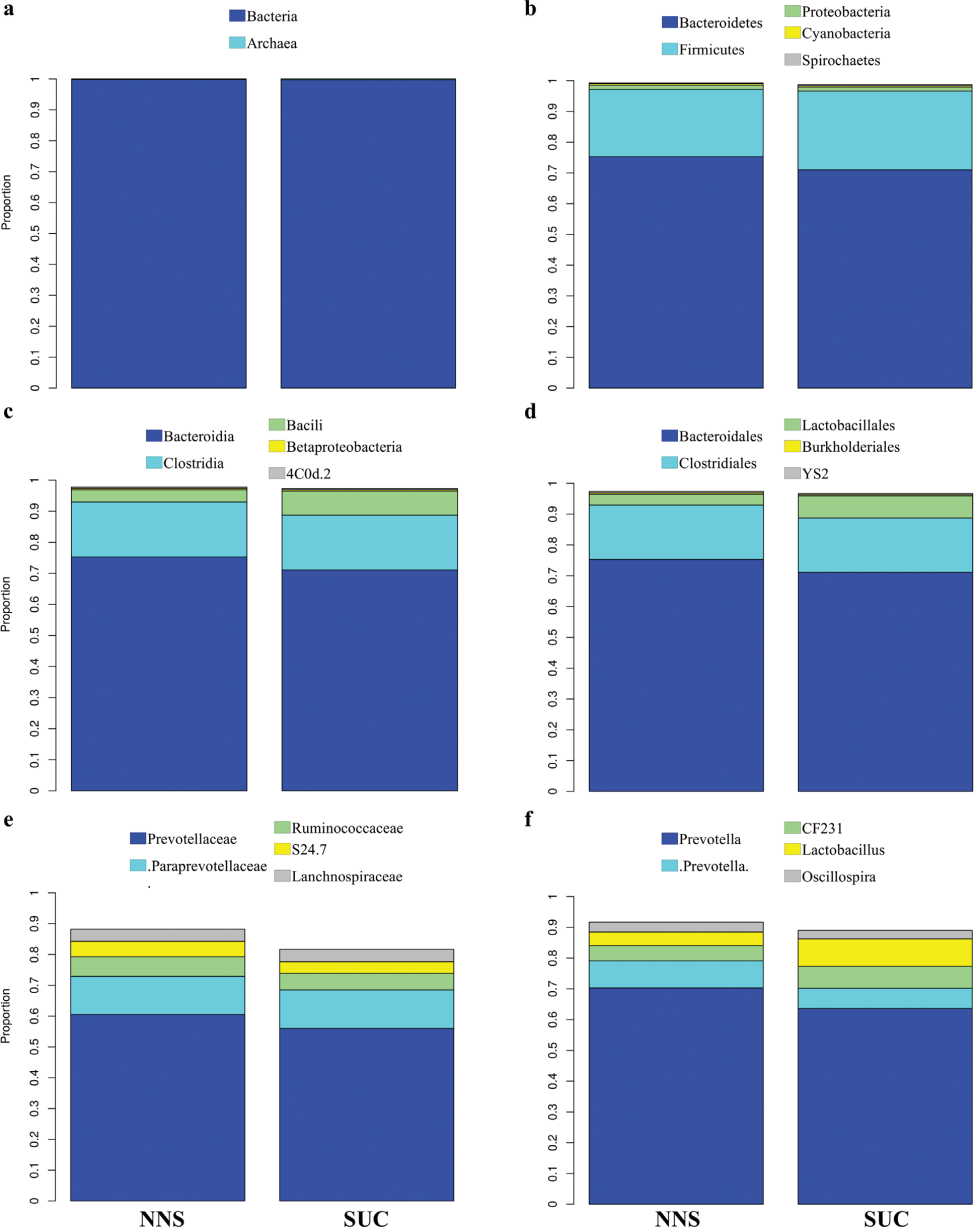
Average proportion of the two represented (a) domains and the five most representative (b) phyla, (c) classes, (d) orders, (e) families, and (f) genera among the experimental groups. Color key at the top of each figure. NNS, non-nutritive sweetener (n = 10); SUC, sucrose (n = 9).

Diversity analyses indicated no significant difference between the NNS or SUC groups regarding species richness (Figure 1a), or Shannon (ranging from 3.410 to 4.574) or Simpson (ranging from 0.891 to 0.975) (File S1) diversity indices (p = 0.297 and t = -1.085, p = 0.551 and t = 0.611, p = 0.198 and t = 1.356, respectively). Samples show overlapping rarefaction curves and a similar number of species per read counts per sample (Figure 1b). The weighted beta diversity approximates the centroids, mixing samples from both groups (Figure 1c). Proportion data for all identified taxa, analyzed one by one at all taxonomic levels, were similar in the NNS and SUC groups, with no significant differences (Figure 2, File S2).

## Discussion

Concerns about chronic intake of NNS have been raised in recent years. However, here we present evidence that addition of NNS- or SUC-sweetened yogurt to a standard chow diet for 17 weeks yielded similar microbiota composition and high diversity indices.

We previously described that Wistar rats fed yogurt supplemented with a daily recommended dose of commercial NNS for 17 weeks gained more weight than animals fed sucrose-supplemented yogurt. This phenomenon could be partially explained by a decrease in energy expenditure at rest (Pinto *et al.*, 2017). Our findings are in line with epidemiological studies that have associated NNS with weight gain, the metabolic syndrome, type 2 diabetes mellitus, and cardiovascular disease (Colditz *et al.*, 1990; Dhingra *et al.*, 2007; Fowler *et al.*, 2008; Lutsey *et al.*, 2008). Other mechanisms have also been proposed to explain this counterintuitive effect, such as NNS interaction with sweet receptors followed by changes in glucose absorption and insulin secretion and changes in the gastrointestinal environment (Pepino, 2015).

Because of the intimate relationship between host microbiota and nutritional availability, this mechanism has become widely explored. In fact, evidence points to a harmful effect of NNS on the microbiome, with acesulfame potassium, aspartame, cyclamate, neotame, saccharin, sucralose, and steviol glycosides all potentially associated with dysbiosis (Lobach *et al.*, 2018). Regarding saccharin use, Suez *et al.*, 2014 described 40 altered OTUs and glucose intolerance in mice receiving high doses in tap water for 11 weeks.

Despite these results, in the present study, we found that, when added to the diet at a level equivalent to recommended human adult doses, a commercial NNS widely employed as a food additive had no effect on the rat microbiota, even with chronic consumption. Our results run counter to those of previous studies that offered saccharin at doses far beyond the acceptable human daily intake (ADI) to the animals (Anderson and Kirkland, 1980; Suez *et al.*, 2014). Similar results have been found for other NNS: acesulfame potassium and aspartame, given at 2.5 times or 30 times the ADI, respectively, promoted alterations in the gut microbiota (Suez *et al.*, 2014; Bian *et al.*, 2017a), whereas no such alterations were found when the ADI was respected (Palmnäs *et al.*, 2014; Uebanso *et al.*, 2017). One exception is the gut dysbiosis and liver inflammatory markers seen in mice receiving water supplemented with saccharin at an ADI-equivalent dose (0.3 mg/mL) (Bian *et al.*, 2017b). In this line, Lobach *et al.* (2018) recently raised concerns about the real cause of dysbiosis caused by NNS.

Besides using a quantity of NNS equivalent to usual human exposure, we also mixed the sweetener with a food product. This is a noteworthy difference from previous studies, in which NNS were usually given dissolved in the tap water. We are aware that yogurts contain live microorganisms, and their consumption can alter the gut microbial community (Lisko *et al.*, 2017). We recognize this may be perceived as a limitation of the present study. However, we used a randomized controlled design in which both groups (NNS and SUC) received yogurt, normalizing the potential impact of its components on the gut microbiota. Thus, we understand that the use of yogurt in the present study represents a strength rather than a limitation, as in real-world circumstances, NNS are usually consumed mixed with food and beverages and not in pure water.

Our findings also suggest three other hypotheses that should be tested in future: (i) gut microbiota composition is not altered by saccharin, independently of the sweetener carrier. Unfortunately, most previous studies have tested saccharin in water at doses exceeding the ADI range, which hinders comparisons. One recent study showed changes in gut microbiome and in lung inflammatory markers after 6 months of exposure to saccharin within human ADI range (0.3 mg/mL) (Bian *et al.*, 2017b). Still, a well-designed experiment comparing water to food carriers is necessary; (ii) yogurt may act as the main modifying agent of the gut microbiota; or (iii) yogurt may act to maintain microbiota homeostasis despite NNS exposure.

## Conclusion

Our hypothesis of an association between chronic intake of a commercially available NNS and changes in gut microbiota was refuted. Rats on a prolonged diet of exposure to sucrose or moderate doses of NNS diluted in low-fat yogurt showed a similar microbiota composition pattern.

## Data Access

Raw sequence data and information about the barcodes were submitted to the NCBI BioProject database under the number PRJNA480648.

## Supplementary Material

The following online material is available for this article:

Figure S1 - Yogurt (a) and NNS intake (b) over time.

File S1 - Shannon and Simpson indices of diversity. The name of each sheet indicates the respective index.

File S2 - One-by-one comparison of identified taxa (from phylum to genus).

Table S1 - Operational Taxonomic Unit (OTU) table.
