# Supplementary material for: Metabarcoding reveals that a non-nutritive sweetener and sucrose yield similar gut microbiota patterns in Wistar rats
Source: Genet Mol Biol. 2020 Mar 16;43(1):e20190028. doi: 10.1590/1678-4685-GMB-2019-0028 (PMC7197999; doi:10.1590/1678-4685-GMB-2019-0028)
Supplement: Supplementary file 1 [file 1415-4757-GMB-43-1-e20190028-s4.pdf]

Genetics and Molecular Biology 43, 1 (2020)
Copyright © 2020, Sociedade Brasileira de Genética

Supplementary material to “Metabarcoding reveals that a non-nutritive sweetener and sucrose yield similar gut microbiota patterns in Wistar rats”

Table S1 - Operational Taxonomic Unit (OTU) table. “sn” are the samples that belong to the non-nutritive sweetener (NNS) group; “ss” are the samples that belong to the sucrose (SUC) group. The numbers indicate the number of the rat in the box for internal annotation. Levels 1 to 7: from Domain to the species epithet.

Level_1	Level_2	Level_3	Level_4	Level_5	Level_6	Level_7	ss10	sn15	sn16	ss19	sn20	ss21	ss25	sn26	sn27	ss28	ss2	ss30	sn31	sn35	sn36	sn5	ss6	ss8	sn9
k__Bacteria	p__Bacteroidetes	c__Bacteroidia	o__Bacteroidales	f__Prevotellaceae	g__Prevotella	s__copri	3992	791	421	609	1586	2720	2154	4328	2554	4375	702	596	1017	1	356	1331	4214	1436	640
k__Bacteria	p__Bacteroidetes	c__Bacteroidia	o__Bacteroidales	f__Prevotellaceae	g__Prevotella	s__copri	11106	3791	2708	9440	12851	9132	980	3154	4484	15533	3044	1956	2592	1465	7954	2234	1096	1270	1086
k__Bacteria	p__Bacteroidetes	c__Bacteroidia	o__Bacteroidales	f__[Paraprevotellaceae]	g__CF231	Unclassified	3052	3278	1100	1359	1332	926	1931	247	1381	1469	1842	2419	2016	27	104	1435	2586	2819	662
k__Bacteria	p__Bacteroidetes	c__Bacteroidia	o__Bacteroidales	f__[Paraprevotellaceae]	g__[Prevotella]	Unclassified	1646	61	128	0	1042	3	561	0	785	223	110	24	223	0	45	176	0	6	13
k__Bacteria	p__Bacteroidetes	c__Bacteroidia	o__Bacteroidales	f__Prevotellaceae	g__Prevotella	Unclassified	5465	4454	3318	3719	1325	3345	2577	1973	5939	2135	3271	4677	3181	2913	6161	4542	3947	3389	3343
k__Bacteria	p__Bacteroidetes	c__Bacteroidia	o__Bacteroidales	f__Prevotellaceae	g__Prevotella	s__copri	1629	1267	401	337	2957	555	410	1053	1201	177	224	509	593	19	312	1352	272	7	1146
k__Bacteria	p__Bacteroidetes	c__Bacteroidia	o__Bacteroidales	f__Bacteroidaceae	g__Bacteroides	Unclassified	176	219	29	195	82	109	20	292	99	66	108	424	151	718	317	149	278	1764	643
k__Bacteria	p__Bacteroidetes	c__Bacteroidia	o__Bacteroidales	f__Prevotellaceae	g__Prevotella	s__stercorea	3258	1495	273	0	0	4604	0	1	782	2	184	187	775	1448	3753	459	657	1610	586
k__Bacteria	p__Bacteroidetes	c__Bacteroidia	o__Bacteroidales	Unclassified	Unclassified	Unclassified	47	0	0	0	21	0	0	0	20	25	0	13	4	0	3	5	0	0	0
k__Bacteria	p__Bacteroidetes	c__Bacteroidia	o__Bacteroidales	f__S24-7	Unclassified	Unclassified	260	57	65	0	4	0	44	0	197	0	135	74	60	0	0	68	0	162	62
k__Bacteria	p__Firmicutes	c__Clostridia	o__Clostridiales	Unclassified	Unclassified	Unclassified	454	406	137	3719	1559	243	1	428	763	11	466	510	62	101	238	438	630	920	360

Genetics and Molecular Biology 43, 1 (2020)
Copyright © 2020, Sociedade Brasileira de Genética

Level_1	Level_2	Level_3	Level_4	Level_5	Level_6	Level_7	ss10	sn15	sn16	ss19	sn20	ss21	ss25	sn26	sn27	ss28	ss2	ss30	sn31	sn35	sn36	sn5	ss6	ss8	sn9
k__Bacteria	p__Firmicutes	c__Clostridia	o__Clostridiales	f__Veillonellaceae	g__Phascolarctobacterium	Unclassified	644	88	22	0	173	1295	13	36	175	172	170	30	36	35	49	375	0	1158	1526
k__Bacteria	p__Bacteroidetes	c__Bacteroidia	o__Bacteroidales	f__Prevotellaceae	g__Prevotella	Unclassified	1904	275	36	848	1833	141	78	1261	84	0	666	170	439	365	992	264	881	664	433
k__Bacteria	p__Firmicutes	c__Bacilli	o__Lactobacillales	f__Lactobacillaceae	g__Lactobacillus	Unclassified	1715	265	133	60	855	347	6766	1584	724	2582	11297	187	379	5648	237	2489	2470	266	750
k__Bacteria	p__Bacteroidetes	c__Bacteroidia	o__Bacteroidales	f__Prevotellaceae	g__Prevotella	Unclassified	677	825	2194	34	581	2292	496	1536	1991	100	344	402	1571	6062	2072	749	413	2743	2455
k__Bacteria	p__Bacteroidetes	c__Bacteroidia	o__Bacteroidales	f__Prevotellaceae	g__Prevotella	Unclassified	938	1300	579	384	1048	2077	1313	1102	1006	3119	1314	1721	1349	1960	2512	2140	1536	1846	2329
k__Bacteria	p__Bacteroidetes	c__Bacteroidia	o__Bacteroidales	f__Prevotellaceae	g__Prevotella	Unclassified	2	1	0	1	1	0	1	2	2	0	1	1	1	0	3	2	0	4	3
k__Bacteria	p__Bacteroidetes	c__Bacteroidia	o__Bacteroidales	f__Prevotellaceae	g__Prevotella	Unclassified	1961	1798	760	1145	5802	1760	6517	3873	5536	3412	937	3076	3164	6057	2948	2018	5918	3673	2206
k__Bacteria	p__Bacteroidetes	c__Bacteroidia	o__Bacteroidales	f__S24-7	Unclassified	Unclassified	99	61	64	219	34	32	97	122	191	78	68	43	60	115	194	53	126	323	119
k__Bacteria	p__Firmicutes	c__Bacilli	o__Lactobacillales	f__Lactobacillaceae	g__Lactobacillus	Unclassified	122	13	2	22	4	19	13	9	1	90	31	0	0	0	0	20	75	8	11
k__Bacteria	p__Bacteroidetes	c__Bacteroidia	o__Bacteroidales	f__Prevotellaceae	g__Prevotella	s__copri	812	1375	1111	6	3402	2827	47	502	1048	2399	1589	201	912	85	770	1928	305	21	286
k__Bacteria	p__Bacteroidetes	c__Bacteroidia	o__Bacteroidales	f__[Paraprevotellaceae]	g__[Prevotella]	Unclassified	123	97	1344	0	631	569	0	0	0	0	0	611	0	0	184	191	0	0	0
k__Bacteria	p__Firmicutes	c__Clostridia	o__Clostridiales	f__[Mogibacteriaceae]	g__Mogibacterium	Unclassified	14	0	2	2	0	5	0	0	5	0	0	2	3	0	0	1	0	5	1
k__Bacteria	p__Spirochaetes	c__Spirochaetes	o__Spirochaetales	f__Spirochaetaceae	g__Treponema	Unclassified	85	0	0	379	0	37	228	0	6	0	0	0	45	0	0	3	49	0	1
k__Bacteria	p__Bacteroidetes	c__Bacteroidia	o__Bacteroidales	f__S24-7	Unclassified	Unclassified	83	105	54	48	0	6	56	618	22	5	36	18	14	0	61	25	200	141	211

Copyright © 2020, Sociedade Brasileira de Genética

Level_1	Level_2	Level_3	Level_4	Level_5	Level_6	Level_7	ss10	sn15	sn16	ss19	sn20	ss21	ss25	sn26	sn27	ss28	ss2	ss30	sn31	sn35	sn36	sn5	ss6	ss8	sn9
k__Bacteria	p__WPS-2	Unclassified	Unclassified	Unclassified	Unclassified	Unclassified	102	0	0	0	0	16	0	0	3	0	0	16	2	0	0	8	0	0	0
k__Bacteria	p__Bacteroidetes	c__Bacteroidia	o__Bacteroidales	f__Prevotellaceae	g__Prevotella	s__copri	40	17	31	9	56	11	16	51	38	36	26	46	34	24	84	14	22	14	40
k__Bacteria	p__Bacteroidetes	c__Bacteroidia	o__Bacteroidales	f__Prevotellaceae	g__Prevotella	s__copri	487	281	269	1	497	1448	34	367	347	501	357	71	312	89	731	323	155	3	57
k__Bacteria	p__Bacteroidetes	c__Bacteroidia	o__Bacteroidales	f__Prevotellaceae	g__Prevotella	Unclassified	196	1898	395	670	393	418	1726	326	776	1478	397	348	1195	928	1362	870	1418	1202	1037
k__Bacteria	p__Firmicutes	c__Clostridia	o__Clostridiales	Unclassified	Unclassified	Unclassified	190	68	96	79	53	422	42	155	434	13	149	33	433	17	699	82	129	694	338
k__Bacteria	p__Bacteroidetes	c__Bacteroidia	o__Bacteroidales	f__Prevotellaceae	g__Prevotella	Unclassified	30	9	101	15	145	142	18	15	232	9	71	261	72	37	125	354	66	143	54
k__Bacteria	p__Bacteroidetes	c__Bacteroidia	o__Bacteroidales	f__Prevotellaceae	g__Prevotella	s__copri	348	464	77	2411	1269	418	2602	4995	112	551	50	435	911	1672	267	1085	17	1826	1282
k__Bacteria	p__Bacteroidetes	c__Bacteroidia	o__Bacteroidales	f__Bacteroidaceae	g__Bacteroides	Unclassified	20	412	135	2997	10	94	175	111	71	103	121	107	22	192	117	77	630	471	1053
k__Bacteria	p__Bacteroidetes	c__Bacteroidia	o__Bacteroidales	f__Prevotellaceae	g__Prevotella	Unclassified	467	1005	675	47	0	236	104	0	272	1210	0	391	541	0	1	1243	353	0	0
k__Bacteria	p__Bacteroidetes	c__Bacteroidia	o__Bacteroidales	f__Prevotellaceae	g__Prevotella	Unclassified	269	108	16	0	13	277	112	0	39	15	71	268	78	0	0	216	338	0	0
k__Bacteria	p__Bacteroidetes	c__Bacteroidia	o__Bacteroidales	f__S24-7	Unclassified	Unclassified	111	59	53	18	66	35	28	100	107	60	78	16	13	23	105	23	68	98	7
k__Bacteria	p__Proteobacteria	c__Epsilonproteobacteria	o__Campylobacterales	f__Helicobacteraceae	Unclassified	Unclassified	127	105	107	522	39	51	27	122	69	48	38	336	85	12	12	80	124	83	41
k__Bacteria	p__Bacteroidetes	c__Bacteroidia	o__Bacteroidales	f__S24-7	Unclassified	Unclassified	16	0	17	0	0	2	1	20	0	4	2	0	0	1	0	0	3	13	0
k__Bacteria	p__Actinobacteria	c__Actinobacteria	o__Bifidobacteriales	f__Bifidobacteriaceae	g__Bifidobacterium	Unclassified	146	116	32	83	1	88	0	14	191	1	3	53	33	0	49	1	137	11	1

Copyright © 2020, Sociedade Brasileira de Genética

**Level_1**	**Level_2**	**Level_3**	**Level_4**	**Level_5**	**Level_6**	**Level_7**	**ss10**	**sn15**	**sn16**	**ss19**	**sn20**	**ss21**	**ss25**	**sn26**	**sn27**	**ss28**	**ss2**	**ss30**	**sn31**	**sn35**	**sn36**	**sn5**	**ss6**	**ss8**	**sn9**
k__Bacteria	p__Firmicutes	c__Clostridia	o__Clostridiales	Unclassified	Unclassified	Unclassified	51	25	1	54	1	0	0	33	0	0	0	69	1	1	35	3	115	1	0
k__Bacteria	p__Bacteroidetes	c__Bacteroidia	o__Bacteroidales	f__Porphyromonadaceae	g__Parabacteroides	Unclassified	264	159	105	355	0	160	188	291	128	133	142	206	119	157	135	321	245	244	100
k__Bacteria	p__Firmicutes	c__Bacilli	o__Turicibacterales	f__Turicibacteraceae	g__Turicibacter	Unclassified	347	957	5	1266	179	134	0	61	133	0	122	266	30	170	32	4	90	189	128
k__Bacteria	p__Cyanobacteria	c__4C0d-2	o__YS2	Unclassified	Unclassified	Unclassified	56	11	281	15	43	3	84	0	0	106	12	0	21	78	0	0	47	179	35
k__Bacteria	p__Firmicutes	c__Clostridia	o__Clostridiales	Unclassified	Unclassified	Unclassified	9	0	6	3	1	3	3	7	17	4	6	55	7	27	18	4	21	12	6
k__Bacteria	p__Firmicutes	c__Clostridia	o__Clostridiales	f__Ruminococcaceae	g__Oscillospira	Unclassified	78	52	303	96	59	69	92	184	110	98	43	214	25	228	87	60	72	62	65
k__Bacteria	p__Bacteroidetes	c__Bacteroidia	o__Bacteroidales	f__S24-7	Unclassified	Unclassified	293	12	0	87	42	0	18	0	0	0	0	0	3	0	79	0	247	0	4
k__Bacteria	p__Bacteroidetes	c__Bacteroidia	o__Bacteroidales	f__[Paraprevotellaceae]	g__YRC22	Unclassified	154	206	69	92	153	274	170	77	121	79	349	38	164	59	132	135	103	520	685
k__Bacteria	p__Firmicutes	c__Clostridia	o__Clostridiales	f__Ruminococcaceae	g__Oscillospira	Unclassified	106	25	96	238	24	24	96	138	40	20	24	269	63	94	46	15	147	35	144
k__Bacteria	p__Firmicutes	c__Bacilli	o__Lactobacillales	f__Lactobacillaceae	g__Lactobacillus	Unclassified	147	89	1	350	213	17	20	1062	242	256	396	70	107	73	9	75	1597	4	23
k__Bacteria	p__Bacteroidetes	c__Bacteroidia	o__Bacteroidales	f__[Paraprevotellaceae]	Unclassified	Unclassified	1	0	0	0	0	0	0	0	0	0	0	0	0	0	1	0	1	0	0
k__Bacteria	p__Bacteroidetes	c__Bacteroidia	o__Bacteroidales	f__Prevotellaceae	g__Prevotella	Unclassified	109	135	21	40	8	17	314	0	56	287	163	1	202	23	338	0	0	0	94
k__Bacteria	p__Firmicutes	c__Clostridia	o__Clostridiales	f__Clostridiaceae	Unclassified	Unclassified	299	115	13	244	58	158	0	22	43	2	55	66	66	21	34	7	83	167	47
k__Bacteria	p__Firmicutes	c__Clostridia	o__Clostridiales	f__Ruminococcaceae	Unclassified	Unclassified	235	38	96	138	564	261	240	9	219	312	222	64	116	18	75	261	366	213	271

Copyright © 2020, Sociedade Brasileira de Genética

**Level_1**	**Level_2**	**Level_3**	**Level_4**	**Level_5**	**Level_6**	**Level_7**	**ss10**	**sn15**	**sn16**	**ss19**	**sn20**	**ss21**	**ss25**	**sn26**	**sn27**	**ss28**	**ss2**	**ss30**	**sn31**	**sn35**	**sn36**	**sn5**	**ss6**	**ss8**	**sn9**
k__Bacteria	p__Bacteroidetes	c__Bacteroidia	o__Bacteroidales	f__Prevotellaceae	g__Prevotella	Unclassified	12	62	63	0	21	73	59	0	60	149	121	130	145	53	290	55	23	10	107
k__Bacteria	p__Firmicutes	c__Clostridia	o__Clostridiales	f__Lachnospiraceae	Unclassified	Unclassified	9	5	2	0	0	2	0	11	0	4	1	0	0	0	0	0	0	0	0
k__Bacteria	p__Proteobacteria	c__Betaproteobacteria	o__Burkholderiales	f__Alcaligenaceae	g__Sutterella	Unclassified	358	114	125	232	315	96	33	289	96	11	208	14	80	104	284	36	170	373	380
k__Bacteria	p__Firmicutes	c__Clostridia	o__Clostridiales	f__Clostridiaceae	g__Clostridium	Unclassified	5	2	0	4	0	0	0	0	11	0	0	2	2	5	0	1	0	0	4
k__Bacteria	p__Firmicutes	c__Clostridia	o__Clostridiales	f__Ruminococcaceae	g__Ruminococcus	s__flavefaciens	93	14	0	5	84	176	0	0	185	115	40	41	32	0	84	16	125	153	33
k__Bacteria	p__Firmicutes	c__Clostridia	o__Clostridiales	f__Ruminococcaceae	Unclassified	Unclassified	42	16	16	22	32	35	28	0	33	8	14	32	15	33	31	21	77	13	19
k__Bacteria	p__Firmicutes	c__Bacilli	o__Lactobacillales	f__Lactobacillaceae	g__Lactobacillus	s__reuteri	162	31	8	110	143	30	130	87	14	450	143	9	7	2	0	21	237	37	6
k__Bacteria	p__Firmicutes	c__Clostridia	o__Clostridiales	f__Lachnospiraceae	g__Blautia	Unclassified	40	2	7	0	31	40	6	3	5	41	28	2	1	21	5	6	6	4	4
k__Bacteria	p__Proteobacteria	c__Deltaproteobacteria	o__Desulfovibrionales	f__Desulfovibrionaceae	g__Desulfovibrio	Unclassified	5	0	0	1	0	0	0	0	0	0	1	0	0	0	1	0	1	1	0
k__Bacteria	p__Bacteroidetes	c__Bacteroidia	o__Bacteroidales	f__p-2534-18B5	Unclassified	Unclassified	177	0	0	0	0	22	0	0	77	0	0	149	70	0	0	129	172	460	378
k__Bacteria	p__Bacteroidetes	c__Bacteroidia	o__Bacteroidales	f__Prevotellaceae	g__Prevotella	s__copri	12	3	4	0	7	32	0	1	4	2	5	1	11	1	26	8	5	0	0
k__Bacteria	p__Proteobacteria	c__Betaproteobacteria	Unclassified	Unclassified	Unclassified	Unclassified	3	0	0	81	23	0	0	0	10	5	0	0	0	0	0	0	52	0	0
k__Bacteria	p__Proteobacteria	c__Epsilonproteobacteria	o__Campylobacterales	f__Helicobacteraceae	g__Flexispira	Unclassified	11	0	25	27	4	2	16	0	2	13	0	100	15	7	2	3	3	17	0
k__Bacteria	p__Bacteroidetes	c__Bacteroidia	o__Bacteroidales	f__S24-7	Unclassified	Unclassified	66	0	3	1	15	22	25	6	24	9	20	5	23	0	35	17	19	2	32

Copyright © 2020, Sociedade Brasileira de Genética

Level_1	Level_2	Level_3	Level_4	Level_5	Level_6	Level_7	ss10	sn15	sn16	ss19	sn20	ss21	ss25	sn26	sn27	ss28	ss2	ss30	sn31	sn35	sn36	sn5	ss6	ss8	sn9
k__Bacteria	p__Verrucomicrobia	c__Verruco-5	o__WCHB1-41	f__RFP12	Unclassified	Unclassified	13	0	0	3	4	16	3	0	11	4	11	85	19	0	0	32	14	35	12
k__Bacteria	p__Bacteroidetes	c__Bacteroidia	o__Bacteroidales	f__S24-7	Unclassified	Unclassified	65	21	29	73	17	21	36	20	32	27	37	33	17	20	57	31	39	30	40
k__Bacteria	p__Bacteroidetes	c__Bacteroidia	o__Bacteroidales	Unclassified	Unclassified	Unclassified	71	32	133	26	54	35	41	0	123	12	121	410	24	139	172	160	137	157	145
k__Bacteria	p__Firmicutes	c__Clostridia	o__Clostridiales	Unclassified	Unclassified	Unclassified	89	17	0	31	10	72	0	0	32	17	4	6	7	7	7	29	29	1	12
k__Bacteria	p__Firmicutes	c__Clostridia	o__Clostridiales	f__Ruminococcaceae	g__Oscillospira	Unclassified	47	17	101	79	9	33	36	70	65	52	17	112	19	154	109	35	64	34	18
k__Bacteria	p__Firmicutes	c__Bacilli	o__Lactobacillales	f__Lactobacillaceae	g__Lactobacillus	Unclassified	158	7	0	60	13	1	1	5	39	2	99	12	7	0	0	0	100	1	0
k__Bacteria	p__Firmicutes	c__Clostridia	o__Clostridiales	f__Lachnospiraceae	Unclassified	Unclassified	9	3	6	4	6	3	10	0	26	22	2	0	2	0	0	3	12	1	36
k__Bacteria	p__Bacteroidetes	c__Bacteroidia	o__Bacteroidales	f__S24-7	Unclassified	Unclassified	20	21	25	5	23	43	27	28	53	28	18	28	21	17	88	23	53	41	38
k__Bacteria	p__Bacteroidetes	c__Bacteroidia	o__Bacteroidales	f__S24-7	Unclassified	Unclassified	235	0	0	0	177	0	82	34	61	6	5	0	0	0	0	2	0	192	145
k__Bacteria	p__Firmicutes	c__Clostridia	o__Clostridiales	f__Lachnospiraceae	g__Blautia	Unclassified	193	16	5	2	7	126	0	0	15	154	2	1	0	13	0	14	0	0	2
k__Bacteria	p__Bacteroidetes	c__Bacteroidia	o__Bacteroidales	Unclassified	Unclassified	Unclassified	34	66	240	44	85	175	307	0	204	211	143	257	116	0	0	101	147	18	202
k__Bacteria	p__Firmicutes	c__Clostridia	o__Clostridiales	f__Ruminococcaceae	g__Oscillospira	Unclassified	284	87	106	567	53	164	441	391	115	36	57	297	129	3212	46	140	318	58	37
k__Bacteria	p__Firmicutes	c__Clostridia	o__Clostridiales	f__Lachnospiraceae	Unclassified	Unclassified	9	27	4	0	6	0	4	0	50	0	7	0	13	4	0	1	6	5	35
k__Bacteria	p__Firmicutes	c__Clostridia	o__Clostridiales	f__Lachnospiraceae	Unclassified	Unclassified	51	5	6	14	9	24	9	30	88	30	14	6	11	9	67	27	27	38	33

Copyright © 2020, Sociedade Brasileira de Genética

**Level_1**	**Level_2**	**Level_3**	**Level_4**	**Level_5**	**Level_6**	**Level_7**	**ss10**	**sn15**	**sn16**	**ss19**	**sn20**	**ss21**	**ss25**	**sn26**	**sn27**	**ss28**	**ss2**	**ss30**	**sn31**	**sn35**	**sn36**	**sn5**	**ss6**	**ss8**	**sn9**
k__Bacteria	p__Bacteroidetes	c__Bacteroidia	o__Bacteroidales	f__S24-7	Unclassified	Unclassified	100	527	197	47	367	368	129	1742	521	136	238	339	236	0	687	226	548	238	868
k__Bacteria	p__Firmicutes	c__Clostridia	o__Clostridiales	f__Ruminococcaceae	Unclassified	Unclassified	3	4	15	9	1	2	5	0	3	0	3	11	2	31	5	2	4	9	6
k__Bacteria	p__Firmicutes	c__Clostridia	o__Clostridiales	f__Ruminococcaceae	Unclassified	Unclassified	95	2	16	0	0	31	16	0	20	0	0	2	0	0	12	1	51	16	8
k__Bacteria	p__Spirochaetes	c__Spirochaetes	o__Spirochaetales	f__Spirochaetaceae	g__Treponema	Unclassified	6	2	202	13	9	12	12	2	17	5	1	143	8	229	14	16	0	27	6
k__Bacteria	p__Firmicutes	c__Erysipelotrichi	o__Erysipelotrichales	f__Erysipelotrichaceae	g__Allobaculum	Unclassified	89	24	15	30	6	20	0	123	232	9	8	12	10	71	102	0	140	37	68
k__Bacteria	p__Bacteroidetes	c__Bacteroidia	o__Bacteroidales	Unclassified	Unclassified	Unclassified	7	4	28	21	12	1	6	9	33	13	0	7	3	7	41	5	1	0	17
k__Bacteria	p__Firmicutes	c__Clostridia	o__Clostridiales	f__Veillonellaceae	g__Phascolarctobacterium	Unclassified	1	3	0	0	0	1	0	0	0	1	0	0	0	0	0	0	0	0	0
k__Bacteria	p__Firmicutes	c__Clostridia	o__Clostridiales	f__Lachnospiraceae	Unclassified	Unclassified	146	11	1	23	150	4	32	0	51	58	1	0	0	11	0	5	4	0	0
k__Bacteria	p__Bacteroidetes	c__Bacteroidia	o__Bacteroidales	Unclassified	Unclassified	Unclassified	9	0	0	2	12	7	9	0	8	2	3	21	13	10	0	17	5	8	13
k__Bacteria	p__Bacteroidetes	c__Bacteroidia	o__Bacteroidales	f__S24-7	Unclassified	Unclassified	39	4	51	0	9	4	15	19	39	21	41	8	12	21	17	2	6	1	24
k__Bacteria	p__Firmicutes	c__Clostridia	o__Clostridiales	f__Ruminococcaceae	g__Oscillospira	Unclassified	25	37	88	24	1	66	20	18	3	7	5	48	3	184	2	2	34	45	13
k__Bacteria	p__Firmicutes	c__Clostridia	o__Clostridiales	f__Ruminococcaceae	Unclassified	Unclassified	45	11	0	6	0	2	82	55	0	0	2	164	6	3	9	1	68	2	27
k__Bacteria	p__Bacteroidetes	c__Bacteroidia	o__Bacteroidales	f__[Paraprevotellaceae]	g__CF231	Unclassified	25	3	8	3	8	52	932	8	0	0	1	0	52	0	10	74	438	7	111
k__Bacteria	p__Firmicutes	c__Clostridia	o__Clostridiales	f__Lachnospiraceae	Unclassified	Unclassified	47	44	0	35	21	7	0	0	11	6	2	0	0	0	5	0	1	0	0

Copyright © 2020, Sociedade Brasileira de Genética

Level_1	Level_2	Level_3	Level_4	Level_5	Level_6	Level_7	ss10	sn15	sn16	ss19	sn20	ss21	ss25	sn26	sn27	ss28	ss2	ss30	sn31	sn35	sn36	sn5	ss6	ss8	sn9
k__Bacteria	p__Bacteroidetes	c__Bacteroidia	o__Bacteroidales	f__Prevotellaceae	g__Prevotella	Unclassified	95	103	299	186	493	356	106	719	169	86	93	1127	313	314	396	140	258	313	447
k__Bacteria	p__Firmicutes	c__Clostridia	o__Clostridiales	Unclassified	Unclassified	Unclassified	104	38	61	44	116	87	119	14	207	87	46	106	81	60	8	52	153	49	47
k__Bacteria	p__Firmicutes	c__Erysipelotrichi	o__Erysipelotrichales	f__Erysipelotrichaceae	Unclassified	Unclassified	6	11	8	0	8	16	13	0	15	4	1	6	2	0	9	21	5	0	6
k__Bacteria	p__Tenericutes	c__Mollicutes	o__RF39	Unclassified	Unclassified	Unclassified	13	46	0	4	0	0	1	0	0	0	4	0	0	0	0	40	76	0	12
k__Bacteria	p__Firmicutes	c__Clostridia	o__Clostridiales	f__Ruminococcaceae	g__Oscillospira	Unclassified	39	33	149	360	10	49	46	51	29	24	20	153	31	153	43	25	43	33	19
k__Bacteria	p__Firmicutes	c__Clostridia	o__Clostridiales	f__Ruminococcaceae	g__Ruminococcus	Unclassified	7	0	0	0	0	0	0	0	0	0	0	0	1	0	0	0	0	0	0
k__Bacteria	p__Bacteroidetes	c__Bacteroidia	o__Bacteroidales	f__RF16	Unclassified	Unclassified	74	34	24	133	13	95	250	229	134	37	80	172	32	191	26	202	31	158	133
k__Bacteria	p__Firmicutes	c__Clostridia	o__Clostridiales	f__Lachnospiraceae	Unclassified	Unclassified	95	0	1	67	42	0	0	0	20	0	0	8	0	0	0	2	5	1	12
k__Bacteria	p__Bacteroidetes	c__Bacteroidia	o__Bacteroidales	f__S24-7	Unclassified	Unclassified	11	0	0	0	0	2	5	51	17	3	0	0	0	15	5	0	25	0	2
k__Bacteria	p__Bacteroidetes	c__Bacteroidia	o__Bacteroidales	f__Prevotellaceae	g__Prevotella	Unclassified	13	8	17	16	3	17	19	29	10	16	24	68	21	35	35	25	18	41	32
k__Bacteria	p__Firmicutes	c__Clostridia	o__Clostridiales	f__Veillonellaceae	Unclassified	Unclassified	128	60	1103	679	97	129	117	272	67	73	113	788	52	2071	127	87	96	3	14
k__Bacteria	p__Firmicutes	c__Clostridia	o__Clostridiales	f__Ruminococcaceae	Unclassified	Unclassified	23	25	147	74	26	80	45	21	36	28	21	115	44	422	125	14	37	25	29
k__Bacteria	p__Firmicutes	c__Clostridia	o__Clostridiales	f__Ruminococcaceae	Unclassified	Unclassified	154	41	16	12	44	37	0	0	25	26	13	41	7	29	42	39	0	6	21
k__Bacteria	p__Firmicutes	c__Erysipelotrichi	o__Erysipelotrichales	f__Erysipelotrichaceae	g__Allobaculum	Unclassified	24	28	0	19	0	78	2	202	68	7	8	11	12	2	5	1	159	10	37

Copyright © 2020, Sociedade Brasileira de Genética

Level_1	Level_2	Level_3	Level_4	Level_5	Level_6	Level_7	ss10	sn15	sn16	ss19	sn20	ss21	ss25	sn26	sn27	ss28	ss2	ss30	sn31	sn35	sn36	sn5	ss6	ss8	sn9
k__Bacteria	p__Bacteroidetes	c__Bacteroidia	o__Bacteroidales	f__Prevotellaceae	g__Prevotella	Unclassified	9	4	0	0	0	9	0	0	7	0	1	1	2	4	25	1	1	0	2
k__Bacteria	p__Bacteroidetes	c__Bacteroidia	o__Bacteroidales	f__S24-7	Unclassified	Unclassified	68	0	0	0	32	60	0	0	41	0	0	88	53	0	0	0	9	0	0
k__Bacteria	p__Firmicutes	c__Clostridia	o__Clostridiales	f__Lachnospiraceae	Unclassified	Unclassified	36	0	0	0	0	4	0	0	21	0	1	0	0	4	0	3	0	0	0
k__Bacteria	p__Firmicutes	c__Clostridia	o__Clostridiales	f__Ruminococcaceae	Unclassified	Unclassified	9	4	31	48	2	3	6	3	7	2	1	68	5	112	7	10	2	1	0
k__Bacteria	p__Firmicutes	c__Clostridia	o__Clostridiales	f__[Mogibacteriaceae]	Unclassified	Unclassified	10	11	20	3	5	13	11	18	9	17	10	33	8	26	12	10	15	18	9
k__Bacteria	Unclassified	Unclassified	Unclassified	Unclassified	Unclassified	Unclassified	47	19	0	0	0	197	0	0	0	0	12	0	19	0	0	70	0	0	0
k__Bacteria	p__Firmicutes	c__Clostridia	o__Clostridiales	f__Ruminococcaceae	Unclassified	Unclassified	2	1	14	12	3	3	4	10	15	9	12	18	1	10	3	9	6	9	5
k__Bacteria	p__Elusimicrobia	c__Elusimicrobia	o__Elusimicrobiales	f__Elusimicrobiaceae	Unclassified	Unclassified	36	59	107	29	37	121	215	49	118	120	4	510	37	201	51	114	9	185	14
k__Bacteria	p__Firmicutes	c__Clostridia	o__Clostridiales	f__Ruminococcaceae	g__Oscillospira	Unclassified	55	7	35	62	28	4	18	40	54	39	15	202	25	30	83	39	104	8	63
k__Bacteria	p__Bacteroidetes	c__Bacteroidia	o__Bacteroidales	f__S24-7	Unclassified	Unclassified	18	115	99	97	111	17	188	354	55	21	159	112	58	43	88	66	55	104	305
k__Bacteria	p__Bacteroidetes	c__Bacteroidia	o__Bacteroidales	f__[Paraprevotellaceae]	g__[Prevotella]	Unclassified	147	306	77	1245	2	194	0	1290	252	145	569	321	243	5	26	1512	30	2490	1490
k__Bacteria	p__Bacteroidetes	c__Bacteroidia	o__Bacteroidales	Unclassified	Unclassified	Unclassified	5	7	1	0	2	2	0	17	7	2	0	3	0	0	0	0	0	11	9
k__Bacteria	p__Firmicutes	c__Clostridia	o__Clostridiales	Unclassified	Unclassified	Unclassified	24	12	99	67	25	18	8	30	28	7	5	184	34	105	32	15	21	15	21
k__Bacteria	p__Firmicutes	c__Clostridia	o__Clostridiales	Unclassified	Unclassified	Unclassified	5	0	0	0	5	4	1	4	9	3	0	7	1	2	0	0	1	2	1

Copyright © 2020, Sociedade Brasileira de Genética

Level_1	Level_2	Level_3	Level_4	Level_5	Level_6	Level_7	ss10	sn15	sn16	ss19	sn20	ss21	ss25	sn26	sn27	ss28	ss2	ss30	sn31	sn35	sn36	sn5	ss6	ss8	sn9
k__Bacteria	p__Firmicutes	c__Clostridia	o__Clostridiales	f__Lachnospiraceae	Unclassified	Unclassified	32	1	0	246	1	68	49	0	23	6	33	6	47	498	27	83	21	107	231
k__Bacteria	p__Firmicutes	c__Bacilli	o__Lactobacillales	f__Lactobacillaceae	g__Lactobacillus	Unclassified	115	0	2	0	0	0	0	0	29	0	0	0	9	0	16	0	11	12	0
k__Bacteria	p__Firmicutes	c__Clostridia	o__Clostridiales	f__Ruminococcaceae	g__Oscillospira	Unclassified	1	0	0	0	1	0	0	0	4	0	1	0	1	1	0	0	0	0	2
k__Bacteria	p__Proteobacteria	c__Deltaproteobacteria	o__Desulfovibrionales	f__Desulfovibrionaceae	Unclassified	Unclassified	43	62	389	169	51	69	32	59	27	33	22	467	64	129	45	26	91	24	34
k__Bacteria	Unclassified	Unclassified	Unclassified	Unclassified	Unclassified	Unclassified	1	0	0	0	0	0	0	0	0	0	0	0	0	0	0	0	1	3	1
k__Bacteria	p__Firmicutes	c__Clostridia	o__Clostridiales	f__Lachnospiraceae	g__Blautia	Unclassified	19	30	7	2	27	27	19	6	12	5	3	8	1	3	6	4	29	7	7
k__Bacteria	p__Bacteroidetes	c__Bacteroidia	o__Bacteroidales	f__p-2534-18B5	Unclassified	Unclassified	14	116	120	0	75	12	0	0	4	0	109	0	5	0	0	64	0	206	0
k__Archaea	p__Euryarchaeota	c__Thermoplasmata	o__E2	f__[Methanomassiliicoccaceae]	g__vadinCA11	Unclassified	75	8	3	22	61	79	0	0	75	80	12	327	69	72	82	42	63	196	34
k__Bacteria	p__Bacteroidetes	c__Bacteroidia	o__Bacteroidales	f__S24-7	Unclassified	Unclassified	34	7	25	41	27	36	6	99	75	10	2	43	33	0	15	11	34	83	79
k__Bacteria	p__Firmicutes	c__Clostridia	o__Clostridiales	f__Ruminococcaceae	g__Ruminococcus	Unclassified	53	86	2	5	29	18	0	67	38	0	7	130	3	0	2	23	25	6	43
k__Bacteria	p__Firmicutes	Unclassified	Unclassified	Unclassified	Unclassified	Unclassified	4	2	4	3	0	3	3	0	8	0	0	0	1	4	0	6	12	8	2
k__Bacteria	p__Bacteroidetes	c__Bacteroidia	o__Bacteroidales	f__Porphyromonadaceae	g__Parabacteroides	Unclassified	17	25	38	267	50	17	112	218	19	33	56	31	9	198	177	14	107	221	258
k__Bacteria	p__Firmicutes	c__Clostridia	o__Clostridiales	f__Ruminococcaceae	g__Ruminococcus	Unclassified	30	0	0	0	1	0	0	35	3	4	30	0	0	0	3	19	6	2	0
k__Archaea	p__Euryarchaeota	c__Methanobacteria	o__Methanobacteriales	f__Methanobacteriaceae	g__Methanobrevibacter	Unclassified	13	5	19	3	20	23	8	0	37	0	44	42	43	12	10	32	12	25	54

Copyright © 2020, Sociedade Brasileira de Genética

**Level_1**	**Level_2**	**Level_3**	**Level_4**	**Level_5**	**Level_6**	**Level_7**	**ss10**	**sn15**	**sn16**	**ss19**	**sn20**	**ss21**	**ss25**	**sn26**	**sn27**	**ss28**	**ss2**	**ss30**	**sn31**	**sn35**	**sn36**	**sn5**	**ss6**	**ss8**	**sn9**
k__Bacteria	p__Bacteroidetes	c__Bacteroidia	o__Bacteroidales	f__Prevotellaceae	g__Prevotella	Unclassified	129	71	96	366	173	59	288	31	110	106	272	113	96	7	94	306	391	248	139
k__Bacteria	p__Firmicutes	c__Clostridia	o__Clostridiales	f__Ruminococcaceae	Unclassified	Unclassified	85	0	0	0	0	0	0	0	0	0	0	0	0	0	0	0	0	0	0
k__Bacteria	p__Firmicutes	c__Clostridia	o__Clostridiales	Unclassified	Unclassified	Unclassified	23	79	1462	1	125	1	246	62	3	6	0	38	0	515	300	5	15	20	0
k__Bacteria	p__Bacteroidetes	c__Bacteroidia	o__Bacteroidales	f__Prevotellaceae	Unclassified	Unclassified	40	21	17	68	8	20	36	41	57	10	22	49	28	37	58	43	47	73	47
k__Bacteria	p__Bacteroidetes	c__Bacteroidia	o__Bacteroidales	Unclassified	Unclassified	Unclassified	42	18	8	0	0	10	14	32	9	3	14	0	0	42	9	0	39	10	1
k__Bacteria	p__Firmicutes	c__Clostridia	o__Clostridiales	f__Ruminococcaceae	g__Oscillospira	Unclassified	34	16	51	87	16	18	13	33	6	3	12	109	19	89	13	6	19	45	37
k__Bacteria	p__Firmicutes	c__Clostridia	o__Clostridiales	f__Ruminococcaceae	Unclassified	Unclassified	13	0	5	9	3	9	1	3	35	10	3	14	9	33	32	2	6	9	5
k__Bacteria	p__Bacteroidetes	c__Bacteroidia	o__Bacteroidales	f__Prevotellaceae	g__Prevotella	Unclassified	5	5	5	6	4	11	13	6	15	4	14	26	11	12	33	8	14	5	11
k__Bacteria	p__Firmicutes	c__Clostridia	o__Clostridiales	f__Ruminococcaceae	g__Oscillospira	Unclassified	3	2	10	10	3	1	4	3	4	0	1	11	0	6	3	3	7	3	1
k__Bacteria	p__Firmicutes	c__Bacilli	o__Lactobacillales	f__Streptococcaceae	g__Streptococcus	Unclassified	28	6	0	5	149	21	139	27	21	19	11	2	12	2	2	11	12	1	9
k__Bacteria	p__Firmicutes	c__Clostridia	o__Clostridiales	Unclassified	Unclassified	Unclassified	55	65	716	26	122	32	0	13	209	10	7	3	38	25	296	11	11	9	118
k__Bacteria	Unclassified	Unclassified	Unclassified	Unclassified	Unclassified	Unclassified	16	1	0	3	0	5	0	0	0	0	7	0	0	0	0	3	4	19	2
k__Bacteria	p__Bacteroidetes	c__Bacteroidia	o__Bacteroidales	f__Porphyromonadaceae	g__Parabacteroides	Unclassified	6	10	8	1284	85	36	29	0	23	33	18	59	24	47	7	29	45	31	15
k__Bacteria	p__Firmicutes	c__Clostridia	o__Clostridiales	f__Ruminococcaceae	Unclassified	Unclassified	2	0	0	1	2	0	0	0	0	0	0	3	0	0	0	0	4	2	0

Copyright © 2020, Sociedade Brasileira de Genética

Level_1	Level_2	Level_3	Level_4	Level_5	Level_6	Level_7	ss10	sn15	sn16	ss19	sn20	ss21	ss25	sn26	sn27	ss28	ss2	ss30	sn31	sn35	sn36	sn5	ss6	ss8	sn9
k__Bacteria	p__Firmicutes	c__Clostridia	o__Clostridiales	f__Ruminococcaceae	Unclassified	Unclassified	1	0	7	0	10	0	8	11	3	0	1	0	0	0	0	1	1	1	0
k__Bacteria	p__Firmicutes	c__Clostridia	o__Clostridiales	f__Ruminococcaceae	Unclassified	Unclassified	18	3	31	3	2	8	3	0	11	6	3	11	11	22	23	6	9	49	7
k__Bacteria	p__Firmicutes	c__Clostridia	o__Clostridiales	Unclassified	Unclassified	Unclassified	2	1	3	1	3	1	1	0	8	4	2	2	2	2	5	1	6	11	10
k__Bacteria	p__Bacteroidetes	c__Bacteroidia	o__Bacteroidales	Unclassified	Unclassified	Unclassified	12	1	37	0	0	18	25	0	30	26	8	51	42	0	21	12	0	39	24
k__Bacteria	p__Firmicutes	c__Clostridia	o__Clostridiales	f__Ruminococcaceae	Unclassified	Unclassified	3	0	4	0	15	1	30	6	14	9	1	17	5	7	2	2	5	1	0
k__Bacteria	p__Firmicutes	c__Clostridia	o__Clostridiales	Unclassified	Unclassified	Unclassified	42	58	0	80	69	135	79	0	105	20	0	30	23	9	16	41	106	78	40
k__Bacteria	p__Bacteroidetes	Unclassified	Unclassified	Unclassified	Unclassified	Unclassified	29	11	4	0	7	23	17	15	16	6	10	3	9	2	0	15	31	45	9
k__Bacteria	p__Firmicutes	c__Clostridia	o__Clostridiales	Unclassified	Unclassified	Unclassified	13	3	4	11	5	14	5	13	7	4	6	4	1	15	2	2	11	8	13
k__Bacteria	p__Firmicutes	c__Clostridia	o__Clostridiales	f__[Mogibacteriaceae]	Unclassified	Unclassified	2	1	3	2	3	2	2	4	5	1	2	1	0	16	1	2	2	1	0
k__Bacteria	p__Bacteroidetes	c__Bacteroidia	o__Bacteroidales	f__Rikenellaceae	Unclassified	Unclassified	4	4	2	13	3	2	6	26	1	4	6	22	0	6	0	5	2	12	20
k__Bacteria	p__Bacteroidetes	c__Bacteroidia	o__Bacteroidales	f__S24-7	Unclassified	Unclassified	35	44	70	199	32	54	13	8	49	11	95	53	21	0	26	31	245	90	90
k__Bacteria	p__Firmicutes	c__Clostridia	o__Clostridiales	f__Ruminococcaceae	Unclassified	Unclassified	4	0	1	4	2	4	4	0	4	8	0	2	6	16	4	0	3	0	0
k__Bacteria	p__Firmicutes	c__Clostridia	o__Clostridiales	f__Ruminococcaceae	Unclassified	Unclassified	6	2	5	2	8	10	15	9	17	6	1	9	5	20	6	12	10	0	24
k__Bacteria	p__Firmicutes	c__Clostridia	o__Clostridiales	f__Lachnospiraceae	Unclassified	Unclassified	2	0	0	4	0	0	1	0	0	2	0	0	1	2	0	0	0	2	2

Copyright © 2020, Sociedade Brasileira de Genética

Level_1	Level_2	Level_3	Level_4	Level_5	Level_6	Level_7	ss10	sn15	sn16	ss19	sn20	ss21	ss25	sn26	sn27	ss28	ss2	ss30	sn31	sn35	sn36	sn5	ss6	ss8	sn9
k__Bacteria	p__Firmicutes	c__Clostridia	o__Clostridiales	f__Ruminococcaceae	Unclassified	Unclassified	155	46	20	56	0	0	3	0	4	0	266	80	100	16	1	0	147	28	165
k__Bacteria	p__Firmicutes	c__Clostridia	o__Clostridiales	Unclassified	Unclassified	Unclassified	15	7	30	17	14	27	22	12	48	14	10	13	12	27	26	16	4	17	21
k__Bacteria	p__Bacteroidetes	c__Bacteroidia	o__Bacteroidales	f__S24-7	Unclassified	Unclassified	9	27	12	3	21	2	17	9	2	7	6	5	8	4	21	6	10	5	0
k__Bacteria	p__Bacteroidetes	c__Bacteroidia	o__Bacteroidales	Unclassified	Unclassified	Unclassified	21	14	38	45	39	34	131	66	113	42	29	228	44	70	111	12	43	100	29
k__Bacteria	p__Firmicutes	c__Clostridia	o__Clostridiales	f__Ruminococcaceae	Unclassified	Unclassified	19	68	16	19	14	11	10	179	114	0	0	40	6	3	59	49	0	14	35
k__Bacteria	p__Bacteroidetes	c__Bacteroidia	o__Bacteroidales	f__[Paraprevotellaceae]	Unclassified	Unclassified	1	0	0	9	0	2	0	1	0	1	0	0	3	1	4	3	1	1	1
k__Bacteria	p__Bacteroidetes	c__Bacteroidia	o__Bacteroidales	f__[Paraprevotellaceae]	g__CF231	Unclassified	17	30	21	80	38	89	0	799	20	0	92	20	27	0	0	204	85	182	426
k__Bacteria	p__Firmicutes	c__Clostridia	o__Clostridiales	Unclassified	Unclassified	Unclassified	19	12	256	13	14	20	109	33	44	4	39	375	45	61	54	36	68	90	13
k__Bacteria	p__Firmicutes	c__Clostridia	o__Clostridiales	Unclassified	Unclassified	Unclassified	17	0	0	0	3	58	0	0	1	0	1	0	75	1	0	0	149	12	11
k__Bacteria	p__Bacteroidetes	c__Bacteroidia	o__Bacteroidales	f__[Odoribacteraceae]	g__Butyricimonas	Unclassified	2	1	0	11	1	7	11	0	9	0	5	16	2	10	9	3	10	4	3
k__Bacteria	p__Firmicutes	c__Clostridia	o__Clostridiales	Unclassified	Unclassified	Unclassified	2	1	0	3	0	0	0	0	5	0	0	0	1	0	2	0	4	0	5
k__Bacteria	p__Firmicutes	c__Clostridia	o__Clostridiales	f__Lachnospiraceae	g__Coprococcus	Unclassified	8	41	0	55	17	38	30	25	25	31	14	7	3	87	2	52	24	49	12
k__Bacteria	p__Firmicutes	c__Clostridia	o__Clostridiales	Unclassified	Unclassified	Unclassified	25	3	1	2	163	82	30	2	0	0	2	6	162	4	2	5	263	61	45
k__Bacteria	p__Bacteroidetes	c__Bacteroidia	o__Bacteroidales	f__Porphyromonadaceae	g__Parabacteroides	Unclassified	11	16	10	8	66	23	0	35	40	0	21	54	21	537	98	14	72	201	269

Copyright © 2020, Sociedade Brasileira de Genética

Level_1	Level_2	Level_3	Level_4	Level_5	Level_6	Level_7	ss10	sn15	sn16	ss19	sn20	ss21	ss25	sn26	sn27	ss28	ss2	ss30	sn31	sn35	sn36	sn5	ss6	ss8	sn9
k__Bacteria	p__Firmicutes	c__Clostridia	o__Clostridiales	f__Ruminococcaceae	g__Oscillospira	Unclassified	9	12	31	15	26	14	7	22	6	13	10	26	6	72	36	15	14	30	6
k__Bacteria	p__Firmicutes	c__Clostridia	o__Clostridiales	f__Lachnospiraceae	g__Blautia	Unclassified	47	0	3	3	26	43	0	1	16	46	2	4	5	1	0	9	0	1	1
k__Bacteria	p__Bacteroidetes	c__Bacteroidia	o__Bacteroidales	f__S24-7	Unclassified	Unclassified	15	0	0	11	0	0	0	0	0	0	0	0	0	0	0	0	0	37	9
k__Bacteria	p__Firmicutes	c__Clostridia	o__Clostridiales	f__Ruminococcaceae	g__Oscillospira	Unclassified	40	19	124	228	79	18	70	44	50	26	31	292	39	133	76	35	37	16	47
k__Bacteria	p__Firmicutes	c__Clostridia	o__Clostridiales	f__Ruminococcaceae	Unclassified	Unclassified	7	4	11	4	6	7	7	2	16	10	12	12	3	16	16	14	9	5	8
k__Bacteria	p__Firmicutes	c__Clostridia	o__Clostridiales	Unclassified	Unclassified	Unclassified	10	1	7	2	63	62	101	16	117	52	25	5	7	124	9	50	24	16	86
k__Bacteria	p__Firmicutes	c__Clostridia	o__Clostridiales	Unclassified	Unclassified	Unclassified	5	1	13	0	3	3	2	20	0	65	1	0	0	0	2	0	38	63	2
k__Bacteria	p__Firmicutes	c__Clostridia	o__Clostridiales	f__Ruminococcaceae	Unclassified	Unclassified	2	0	1	0	1	0	1	0	5	0	0	0	0	1	5	1	1	0	0
k__Bacteria	p__Firmicutes	c__Clostridia	o__Clostridiales	f__Lachnospiraceae	Unclassified	Unclassified	3	0	27	1	0	1	0	0	0	0	0	0	2	21	32	75	36	258	9
k__Bacteria	p__Firmicutes	c__Clostridia	o__Clostridiales	f__Lachnospiraceae	g__Roseburia	Unclassified	37	252	19	68	15	4	5	0	143	0	23	385	5	3	39	166	4	234	270
k__Bacteria	p__Firmicutes	c__Erysipelotrichi	o__Erysipelotrichales	f__Erysipelotrichaceae	g__Allobaculum	Unclassified	33	5	7	15	1	1	0	22	8	1	0	6	13	0	12	0	10	7	3
k__Bacteria	p__Bacteroidetes	c__Bacteroidia	o__Bacteroidales	f__S24-7	Unclassified	Unclassified	16	4	6	0	0	2	0	4	0	0	0	0	0	0	0	0	11	0	0
k__Bacteria	p__Firmicutes	c__Clostridia	o__Clostridiales	f__Ruminococcaceae	Unclassified	Unclassified	3	4	45	11	4	8	6	16	4	3	14	33	5	41	13	10	5	6	2
k__Bacteria	p__Firmicutes	c__Erysipelotrichi	o__Erysipelotrichales	f__Erysipelotrichaceae	g__RFN20	Unclassified	5	0	0	0	0	2	0	0	1	0	0	1	1	0	0	2	4	2	0

Copyright © 2020, Sociedade Brasileira de Genética

**Level_1**	**Level_2**	**Level_3**	**Level_4**	**Level_5**	**Level_6**	**Level_7**	**ss10**	**sn15**	**sn16**	**ss19**	**sn20**	**ss21**	**ss25**	**sn26**	**sn27**	**ss28**	**ss2**	**ss30**	**sn31**	**sn35**	**sn36**	**sn5**	**ss6**	**ss8**	**sn9**
k__Bacteria	p__Firmicutes	c__Clostridia	o__Clostridiales	f__Ruminococcaceae	g__Ruminococcus	Unclassified	53	12	2	0	1	0	2	100	3	10	11	1	6	0	6	14	3	0	7
k__Bacteria	p__Bacteroidetes	c__Bacteroidia	o__Bacteroidales	f__Prevotellaceae	g__Prevotella	s__copri	29	51	32	1	73	133	5	24	65	79	61	5	56	2	63	80	15	0	13
k__Bacteria	p__Firmicutes	c__Clostridia	o__Clostridiales	f__Ruminococcaceae	Unclassified	Unclassified	1	0	0	0	2	1	0	0	0	0	0	0	0	0	0	0	9	3	0
k__Bacteria	Unclassified	Unclassified	Unclassified	Unclassified	Unclassified	Unclassified	21	0	0	0	0	0	0	0	0	0	0	57	0	0	0	0	0	0	0
k__Bacteria	p__Firmicutes	c__Bacilli	o__Lactobacillales	f__Streptococcaceae	g__Streptococcus	Unclassified	2	11	5	1	4	9	5	0	3	6	4	0	0	1	2	10	5	1	2
k__Bacteria	p__Firmicutes	c__Clostridia	o__Clostridiales	f__Ruminococcaceae	Unclassified	Unclassified	15	3	1	25	15	4	3	16	17	2	3	28	3	7	0	3	16	20	18
k__Bacteria	p__Bacteroidetes	c__Bacteroidia	o__Bacteroidales	f__Prevotellaceae	g__Prevotella	s__copri	7	0	0	0	3	0	6	3	5	3	0	1	2	0	0	4	8	2	0
k__Bacteria	p__Firmicutes	c__Clostridia	o__Clostridiales	f__Ruminococcaceae	Unclassified	Unclassified	3	0	1	4	0	0	0	0	2	0	4	17	2	2	8	0	2	1	0
k__Bacteria	p__Firmicutes	c__Clostridia	o__Clostridiales	f__Lachnospiraceae	g__Coprococcus	Unclassified	44	22	0	7	0	13	1	0	22	22	0	2	7	8	36	21	25	37	115
k__Bacteria	p__Bacteroidetes	c__Bacteroidia	o__Bacteroidales	f__Prevotellaceae	g__Prevotella	Unclassified	5	2	3	1	3	6	1	28	5	11	7	9	10	5	10	11	14	13	28
k__Bacteria	p__Firmicutes	c__Clostridia	o__Clostridiales	f__Ruminococcaceae	Unclassified	Unclassified	35	24	12	12	109	68	32	32	169	59	9	12	18	61	14	47	17	7	21
k__Bacteria	p__Firmicutes	c__Bacilli	o__Lactobacillales	f__Lactobacillaceae	g__Lactobacillus	Unclassified	27	1	1	0	14	5	147	24	18	57	116	2	16	73	3	54	39	6	16
k__Bacteria	p__Firmicutes	c__Clostridia	o__Clostridiales	f__Veillonellaceae	g__Phascolarctobacterium	Unclassified	2	0	0	0	1	7	0	1	1	1	1	1	0	0	0	1	0	1	13
k__Bacteria	p__Firmicutes	c__Clostridia	o__Clostridiales	f__Ruminococcaceae	Unclassified	Unclassified	9	0	69	1	0	55	66	19	0	4	0	0	17	8	0	28	20	33	1

Copyright © 2020, Sociedade Brasileira de Genética

Level_1	Level_2	Level_3	Level_4	Level_5	Level_6	Level_7	ss10	sn15	sn16	ss19	sn20	ss21	ss25	sn26	sn27	ss28	ss2	ss30	sn31	sn35	sn36	sn5	ss6	ss8	sn9
k__Bacteria	p__Bacteroidetes	c__Bacteroidia	o__Bacteroidales	f__S24-7	Unclassified	Unclassified	17	4	40	0	8	31	4	57	15	13	4	11	10	45	23	3	16	4	22
k__Bacteria	p__Bacteroidetes	c__Bacteroidia	o__Bacteroidales	f__S24-7	Unclassified	Unclassified	13	5	17	0	1	15	0	3	76	2	1	0	5	0	3	0	6	0	0
k__Bacteria	p__Bacteroidetes	c__Bacteroidia	o__Bacteroidales	f__Prevotellaceae	g__Prevotella	Unclassified	21	214	88	72	255	177	14	18	11	37	416	402	25	281	957	132	158	0	140
k__Bacteria	p__Firmicutes	c__Clostridia	o__Clostridiales	f__Lachnospiraceae	Unclassified	Unclassified	20	8	163	86	2	51	35	4	59	11	22	155	23	274	48	97	45	120	220
k__Bacteria	p__Firmicutes	c__Clostridia	o__Clostridiales	f__Lachnospiraceae	Unclassified	Unclassified	5	0	2	10	1	0	12	3	1	0	0	2	2	3	1	1	1	2	0
k__Bacteria	p__Firmicutes	c__Clostridia	o__Clostridiales	f__Lachnospiraceae	Unclassified	Unclassified	13	0	1	0	1	0	12	2	0	1	0	2	0	4	2	0	1	0	0
k__Bacteria	p__Firmicutes	c__Clostridia	o__Clostridiales	f__Peptococcaceae	Unclassified	Unclassified	5	2	21	39	0	6	8	1	5	5	8	46	5	36	11	2	11	5	2
k__Bacteria	p__Firmicutes	c__Clostridia	o__Clostridiales	f__Ruminococcaceae	g__Oscillospira	Unclassified	2	2	1	2	0	1	1	8	3	0	0	17	0	4	8	0	3	1	0
k__Bacteria	p__Bacteroidetes	c__Bacteroidia	o__Bacteroidales	f__S24-7	Unclassified	Unclassified	23	3	16	0	90	3	15	7	6	7	4	2	24	20	149	10	70	7	30
k__Bacteria	p__Proteobacteria	c__Gammaproteobacteria	o__Enterobacteriales	f__Enterobacteriaceae	Unclassified	Unclassified	6	0	0	3	0	0	0	0	0	0	0	0	0	0	0	0	0	0	0
k__Bacteria	p__Bacteroidetes	c__Bacteroidia	o__Bacteroidales	Unclassified	Unclassified	Unclassified	19	0	0	19	4	19	26	0	37	13	15	123	30	28	0	15	88	190	102
k__Bacteria	p__Firmicutes	c__Clostridia	Unclassified	Unclassified	Unclassified	Unclassified	2	2	0	1	3	0	6	2	0	0	0	0	0	0	2	0	12	4	8
k__Bacteria	p__Firmicutes	c__Clostridia	o__Clostridiales	f__Lachnospiraceae	Unclassified	Unclassified	16	2	19	13	5	20	1	0	37	0	12	1	6	11	22	23	10	7	20
k__Bacteria	p__Firmicutes	c__Clostridia	o__Clostridiales	Unclassified	Unclassified	Unclassified	12	0	226	1	2	1	1	3	362	0	145	21	10	0	21	1	93	41	6

Copyright © 2020, Sociedade Brasileira de Genética

Level_1	Level_2	Level_3	Level_4	Level_5	Level_6	Level_7	ss10	sn15	sn16	ss19	sn20	ss21	ss25	sn26	sn27	ss28	ss2	ss30	sn31	sn35	sn36	sn5	ss6	ss8	sn9
k__Bacteria	p__Firmicutes	c__Clostridia	o__Clostridiales	f__[Mogibacteriaceae]	Unclassified	Unclassified	12	3	3	26	11	9	14	23	29	5	3	5	1	11	10	1	4	9	4
k__Bacteria	p__Bacteroidetes	c__Bacteroidia	o__Bacteroidales	f__[Paraprevotellaceae]	g__[Prevotella]	Unclassified	2	1700	1047	17	112	730	1563	1842	1390	1699	1832	1792	905	5197	2027	1580	835	836	2941
k__Bacteria	Unclassified	Unclassified	Unclassified	Unclassified	Unclassified	Unclassified	8	0	3	0	13	7	14	6	12	4	2	8	2	45	20	4	29	20	3
k__Bacteria	p__Firmicutes	c__Clostridia	o__Clostridiales	f__Lachnospiraceae	g__Blautia	Unclassified	7	15	1	0	1	26	5	5	7	1	1	3	6	11	1	2	6	6	3
k__Bacteria	p__Bacteroidetes	c__Bacteroidia	o__Bacteroidales	Unclassified	Unclassified	Unclassified	17	3	6	0	0	2	0	0	4	0	1	5	2	3	0	0	0	0	0
k__Bacteria	p__Firmicutes	c__Clostridia	o__Clostridiales	f__Lachnospiraceae	Unclassified	Unclassified	9	8	3	0	15	31	0	0	5	0	1	0	1	0	0	0	0	3	1
k__Bacteria	p__Bacteroidetes	c__Bacteroidia	o__Bacteroidales	f__Prevotellaceae	g__Prevotella	s__copri	3	5	0	2	2	5	0	0	1	8	1	1	0	0	5	2	0	0	0
k__Bacteria	p__Firmicutes	c__Clostridia	o__Clostridiales	Unclassified	Unclassified	Unclassified	2	0	1	0	0	1	2	0	0	0	0	0	1	2	2	0	1	1	0
k__Bacteria	p__Bacteroidetes	c__Bacteroidia	o__Bacteroidales	f__Prevotellaceae	g__Prevotella	s__copri	15	23	17	2	33	35	1	14	17	29	32	6	20	1	27	26	14	0	6
k__Bacteria	p__Firmicutes	c__Clostridia	o__Clostridiales	Unclassified	Unclassified	Unclassified	14	24	34	0	3	7	9	17	25	7	17	15	8	0	1	20	6	38	5
k__Bacteria	p__Cyanobacteria	c__4C0d-2	o__YS2	Unclassified	Unclassified	Unclassified	9	0	37	0	0	0	0	0	0	1	0	235	0	0	0	9	3	0	7
k__Bacteria	p__Firmicutes	c__Clostridia	o__Clostridiales	f__Lachnospiraceae	g__Coprococcus	Unclassified	30	5	0	0	9	1	0	0	0	2	0	1	11	3	0	0	0	3	0
k__Bacteria	p__Bacteroidetes	c__Bacteroidia	o__Bacteroidales	f__S24-7	Unclassified	Unclassified	19	4	135	0	26	6	8	1	76	64	14	37	39	6	105	5	76	0	15
k__Bacteria	p__Bacteroidetes	c__Bacteroidia	o__Bacteroidales	f__Prevotellaceae	g__Prevotella	Unclassified	3	0	0	0	1	1	0	0	1	2	0	1	1	0	1	2	0	2	2

Copyright © 2020, Sociedade Brasileira de Genética

Level_1	Level_2	Level_3	Level_4	Level_5	Level_6	Level_7	ss10	sn15	sn16	ss19	sn20	ss21	ss25	sn26	sn27	ss28	ss2	ss30	sn31	sn35	sn36	sn5	ss6	ss8	sn9
k__Bacteria	p__Firmicutes	c__Clostridia	o__Clostridiales	Unclassified	Unclassified	Unclassified	12	1	76	162	4	1	7	123	3	21	34	911	3	17	102	116	0	246	61
k__Bacteria	p__Bacteroidetes	c__Bacteroidia	o__Bacteroidales	Unclassified	Unclassified	Unclassified	19	1	0	2	0	3	0	0	1	7	4	6	1	10	1	1	12	3	1
k__Bacteria	p__Proteobacteria	c__Deltaproteobacteria	o__Desulfovibrionales	f__Desulfovibrionaceae	Unclassified	Unclassified	7	0	0	3	2	8	4	0	11	2	4	7	3	13	10	4	1	6	0
k__Bacteria	p__Firmicutes	c__Clostridia	o__Clostridiales	f__Ruminococcaceae	g__Ruminococcus	Unclassified	2	0	0	0	0	3	5	0	4	0	0	3	2	0	0	0	0	1	0
k__Bacteria	p__Firmicutes	c__Clostridia	o__Clostridiales	f__Lachnospiraceae	Unclassified	Unclassified	8	0	1	0	0	4	1	0	9	0	6	3	2	2	4	0	4	0	2
k__Bacteria	p__Firmicutes	c__Clostridia	o__Clostridiales	Unclassified	Unclassified	Unclassified	6	2	0	6	0	8	6	131	13	2	10	323	4	6	36	8	1	24	5
k__Bacteria	p__Firmicutes	Unclassified	Unclassified	Unclassified	Unclassified	Unclassified	9	11	0	6	4	33	0	0	37	0	11	2	1	15	0	2	14	1	12
k__Bacteria	p__Bacteroidetes	c__Bacteroidia	o__Bacteroidales	f__[Paraprevotellaceae]	Unclassified	Unclassified	2	1	2	0	2	1	0	0	0	2	1	0	1	0	0	0	0	0	0
k__Bacteria	p__Firmicutes	c__Clostridia	o__Clostridiales	f__Ruminococcaceae	g__Ruminococcus	Unclassified	2	1	0	0	1	0	0	0	0	0	0	2	1	0	0	0	2	3	1
k__Bacteria	p__Firmicutes	c__Clostridia	o__Clostridiales	f__Ruminococcaceae	g__Oscillospira	Unclassified	2	1	11	13	0	4	4	0	0	0	0	16	2	5	2	0	0	3	0
k__Bacteria	p__Proteobacteria	c__Deltaproteobacteria	o__Desulfovibrionales	f__Desulfovibrionaceae	g__Desulfovibrio	Unclassified	3	5	37	16	1	10	2	7	21	5	5	86	7	33	15	5	19	2	2
k__Bacteria	p__Firmicutes	c__Clostridia	o__Clostridiales	f__Ruminococcaceae	Unclassified	Unclassified	3	1	2	6	1	2	1	0	0	20	0	1	0	0	3	3	14	5	0
k__Bacteria	p__Bacteroidetes	c__Bacteroidia	o__Bacteroidales	f__S24-7	Unclassified	Unclassified	4	117	91	199	0	52	0	0	234	1	292	172	5	0	24	0	112	0	43
k__Bacteria	p__Firmicutes	c__Clostridia	o__Clostridiales	f__Ruminococcaceae	g__Ruminococcus	Unclassified	1	0	0	1	0	0	0	0	0	0	0	0	0	72	0	2	5	2	0

Copyright © 2020, Sociedade Brasileira de Genética

Level_1	Level_2	Level_3	Level_4	Level_5	Level_6	Level_7	ss10	sn15	sn16	ss19	sn20	ss21	ss25	sn26	sn27	ss28	ss2	ss30	sn31	sn35	sn36	sn5	ss6	ss8	sn9
k__Bacteria	p__Firmicutes	c__Clostridia	o__Clostridiales	f__Ruminococcaceae	Unclassified	Unclassified	2	0	7	12	0	0	4	0	0	1	1	14	2	13	1	1	2	0	3
k__Bacteria	p__Bacteroidetes	c__Bacteroidia	o__Bacteroidales	f__S24-7	Unclassified	Unclassified	29	3	60	42	42	15	11	10	34	24	18	66	15	17	8	48	16	41	6
k__Bacteria	p__Cyanobacteria	c__4C0d-2	o__YS2	Unclassified	Unclassified	Unclassified	33	52	44	81	58	0	39	0	6	321	0	55	0	263	28	504	44	53	9
k__Bacteria	p__Lentisphaerae	c__[Lentisphaeria]	o__Victivallales	f__Victivallaceae	Unclassified	Unclassified	12	2	3	0	2	1	12	0	4	1	6	13	6	12	2	1	11	24	0
k__Bacteria	p__Firmicutes	c__Clostridia	o__Clostridiales	Unclassified	Unclassified	Unclassified	7	0	1	0	1	8	0	7	7	0	24	9	0	0	0	11	6	6	0
k__Bacteria	p__Firmicutes	c__Clostridia	o__Clostridiales	f__Peptococcaceae	g__rc4-4	Unclassified	7	27	1	14	90	46	2	14	54	33	16	5	7	30	26	39	6	14	45
k__Bacteria	p__Firmicutes	c__Erysipelotrichi	o__Erysipelotrichales	f__Erysipelotrichaceae	g__Allobaculum	Unclassified	18	2	0	16	0	0	0	29	17	0	1	0	3	2	5	0	1	1	0
k__Bacteria	p__Firmicutes	c__Clostridia	o__Clostridiales	f__Ruminococcaceae	Unclassified	Unclassified	2	4	2	6	2	1	3	10	1	0	0	20	0	2	2	0	4	0	0
k__Bacteria	p__Bacteroidetes	c__Bacteroidia	o__Bacteroidales	f__[Paraprevotellaceae]	g__CF231	Unclassified	2	1	0	0	1	0	1	0	0	1	1	2	1	0	0	0	1	0	0
k__Bacteria	p__Firmicutes	c__Clostridia	o__Clostridiales	f__Clostridiaceae	g__Clostridium	Unclassified	3	9	12	0	14	52	49	122	94	13	1	123	21	5	4	116	1	25	43
k__Bacteria	p__Firmicutes	c__Clostridia	o__Clostridiales	f__Ruminococcaceae	g__Oscillospira	Unclassified	7	2	32	39	2	6	8	4	2	3	1	42	4	10	3	0	4	3	0
k__Bacteria	p__Firmicutes	c__Bacilli	o__Lactobacillales	f__Lactobacillaceae	g__Lactobacillus	Unclassified	7	2	0	0	5	2	22	5	3	14	44	1	2	21	0	13	5	0	4
k__Bacteria	p__Bacteroidetes	c__Bacteroidia	o__Bacteroidales	f__Rikenellaceae	Unclassified	Unclassified	2	0	1	0	0	1	0	0	7	0	0	0	2	0	0	0	0	1	0
k__Bacteria	p__Bacteroidetes	c__Bacteroidia	o__Bacteroidales	f__S24-7	Unclassified	Unclassified	8	53	21	33	68	22	6	261	67	27	61	121	41	151	52	11	78	77	102

Copyright © 2020, Sociedade Brasileira de Genética

**Level_1**	**Level_2**	**Level_3**	**Level_4**	**Level_5**	**Level_6**	**Level_7**	**ss10**	**sn15**	**sn16**	**ss19**	**sn20**	**ss21**	**ss25**	**sn26**	**sn27**	**ss28**	**ss2**	**ss30**	**sn31**	**sn35**	**sn36**	**sn5**	**ss6**	**ss8**	**sn9**
k__Bacteria	p__Firmicutes	c__Clostridia	o__Clostridiales	Unclassified	Unclassified	Unclassified	18	0	0	0	0	14	7	0	13	1	1	0	3	13	3	12	18	23	7
k__Bacteria	p__Firmicutes	c__Clostridia	o__Clostridiales	f__Lachnospiraceae	Unclassified	Unclassified	5	4	4	3	11	8	1	3	14	6	4	1	2	5	9	4	5	4	13
k__Bacteria	p__Firmicutes	c__Clostridia	o__Clostridiales	f__[Mogibacteriaceae]	Unclassified	Unclassified	9	2	1	38	8	5	3	0	7	1	3	34	3	2	1	4	10	5	5
k__Bacteria	p__Firmicutes	c__Clostridia	o__Clostridiales	f__Lachnospiraceae	g__[Ruminococcus]	s__gnavus	5	2	4	18	14	1	9	7	2	2	0	6	2	3	0	9	5	23	4
k__Bacteria	p__Firmicutes	c__Clostridia	o__Clostridiales	f__Lachnospiraceae	Unclassified	Unclassified	8	1	0	5	0	0	0	1	0	0	0	0	0	0	2	0	0	2	0
k__Bacteria	p__Bacteroidetes	c__Bacteroidia	o__Bacteroidales	f__Prevotellaceae	g__Prevotella	s__copri	10	8	14	4	8	11	1	15	8	7	13	1	5	0	3	10	11	1	3
k__Bacteria	p__Firmicutes	c__Clostridia	o__Clostridiales	f__Ruminococcaceae	Unclassified	Unclassified	8	1	1	0	4	2	7	1	15	1	1	3	0	0	1	1	1	2	5
k__Bacteria	p__Firmicutes	c__Clostridia	o__Clostridiales	f__Christensenellaceae	Unclassified	Unclassified	19	7	23	15	16	13	19	0	25	6	13	22	16	39	16	12	12	24	20
k__Bacteria	p__Actinobacteria	c__Actinobacteria	o__Actinomycetales	f__Micrococcaceae	g__Rothia	Unclassified	1	1	2	0	0	1	1	1	0	0	0	2	0	0	0	5	3	2	1
k__Bacteria	p__Tenericutes	c__Mollicutes	o__RF39	Unclassified	Unclassified	Unclassified	5	4	5	2	1	5	5	1	23	6	3	5	4	25	17	9	21	10	2
k__Bacteria	p__Firmicutes	c__Clostridia	o__Clostridiales	f__[Mogibacteriaceae]	Unclassified	Unclassified	1	1	0	0	2	1	2	0	1	5	0	0	0	1	1	0	0	0	0
k__Bacteria	p__Firmicutes	c__Clostridia	o__Clostridiales	Unclassified	Unclassified	Unclassified	1	1	3	3	2	1	3	4	5	6	5	0	1	2	3	0	3	1	2
k__Bacteria	p__Firmicutes	c__Clostridia	o__Clostridiales	f__Lachnospiraceae	Unclassified	Unclassified	3	1	9	7	3	22	12	1	1	5	2	12	0	0	0	6	50	8	0
k__Bacteria	p__Bacteroidetes	c__Bacteroidia	o__Bacteroidales	Unclassified	Unclassified	Unclassified	3	11	24	39	5	12	13	0	14	0	33	55	10	0	0	2	5	25	5

Genetics and Molecular Biology 43, 1 (2020)
Copyright © 2020, Sociedade Brasileira de Genética

Level_1	Level_2	Level_3	Level_4	Level_5	Level_6	Level_7	ss10	sn15	sn16	ss19	sn20	ss21	ss25	sn26	sn27	ss28	ss2	ss30	sn31	sn35	sn36	sn5	ss6	ss8	sn9
k__Bacteria	p__Bacteroidetes	c__Bacteroidia	o__Bacteroidales	f__Prevotellaceae	g__Prevotella	s__copri	9	2	2	3	23	14	0	0	6	25	4	1	7	0	7	1	1	0	3
k__Bacteria	p__Firmicutes	c__Clostridia	o__Clostridiales	f__Lachnospiraceae	Unclassified	Unclassified	7	13	12	4	1	4	1	4	2	0	0	36	1	23	2	2	2	4	5
k__Bacteria	p__Proteobacteria	c__Deltaproteobacteria	o__Desulfovibrionales	f__Desulfovibrionaceae	Unclassified	Unclassified	5	2	41	66	2	9	7	0	48	2	3	87	8	63	16	4	15	15	9
k__Bacteria	p__Bacteroidetes	c__Bacteroidia	o__Bacteroidales	f__Bacteroidaceae	g__Bacteroides	Unclassified	7	0	0	655	119	0	364	0	7	51	0	7	31	0	0	45	0	0	0
k__Bacteria	p__Firmicutes	c__Clostridia	o__Clostridiales	f__Dehalobacteriaceae	g__Dehalobacterium	Unclassified	2	1	0	0	0	0	0	0	1	0	1	0	0	5	0	1	1	0	0
k__Bacteria	p__Firmicutes	c__Clostridia	o__Clostridiales	Unclassified	Unclassified	Unclassified	12	113	526	296	47	157	119	296	299	106	168	331	115	463	179	141	58	167	196
k__Bacteria	p__Proteobacteria	Unclassified	Unclassified	Unclassified	Unclassified	Unclassified	10	0	0	5	11	1	9	0	9	79	17	7	7	0	0	22	9	13	4
k__Bacteria	p__Firmicutes	c__Clostridia	o__Clostridiales	f__Ruminococcaceae	Unclassified	Unclassified	6	14	35	27	10	1	12	24	5	4	3	39	6	60	11	4	10	9	9
k__Bacteria	p__Bacteroidetes	c__Bacteroidia	o__Bacteroidales	Unclassified	Unclassified	Unclassified	8	4	10	18	6	30	4	47	17	33	19	60	31	45	61	6	42	37	31
k__Bacteria	p__Firmicutes	c__Clostridia	o__Clostridiales	Unclassified	Unclassified	Unclassified	2	0	22	0	2	0	0	1	12	2	0	0	16	12	12	15	3	8	10
k__Bacteria	Unclassified	Unclassified	Unclassified	Unclassified	Unclassified	Unclassified	1	0	3	0	0	0	0	0	0	1	0	1	0	4	0	0	1	3	0
k__Bacteria	p__Firmicutes	c__Clostridia	o__Clostridiales	Unclassified	Unclassified	Unclassified	9	3	37	12	5	0	0	265	14	1	14	15	3	2	2	27	0	4	1
k__Bacteria	p__Firmicutes	c__Clostridia	o__Clostridiales	f__Ruminococcaceae	Unclassified	Unclassified	10	0	0	0	0	8	0	0	19	0	6	3	0	0	2	4	0	0	0
k__Bacteria	p__Verrucomicrobia	c__Verruco-5	o__WCHB1-41	f__RFP12	Unclassified	Unclassified	1	0	0	0	0	1	0	0	1	0	0	4	2	0	0	0	1	2	0

Copyright © 2020, Sociedade Brasileira de Genética

Level_1	Level_2	Level_3	Level_4	Level_5	Level_6	Level_7	ss10	sn15	sn16	ss19	sn20	ss21	ss25	sn26	sn27	ss28	ss2	ss30	sn31	sn35	sn36	sn5	ss6	ss8	sn9
k__Bacteria	p__Firmicutes	c__Clostridia	o__Clostridiales	f__Ruminococcaceae	Unclassified	Unclassified	1	1	2	11	0	1	0	18	1	1	0	7	0	1	1	0	0	4	0
k__Bacteria	p__Firmicutes	c__Clostridia	o__Clostridiales	f__Lachnospiraceae	g__Coprococcus	Unclassified	11	6	25	37	0	13	26	5	6	8	5	99	14	7	3	6	46	19	7
k__Bacteria	p__Bacteroidetes	c__Bacteroidia	o__Bacteroidales	Unclassified	Unclassified	Unclassified	8	6	0	127	18	40	0	0	23	0	6	63	8	9	88	25	58	11	15
k__Bacteria	p__Bacteroidetes	c__Bacteroidia	o__Bacteroidales	f__Prevotellaceae	g__Prevotella	s__copri	5	4	1	64	14	8	7	14	0	15	5	3	2	6	7	10	12	20	16
k__Bacteria	p__Bacteroidetes	c__Bacteroidia	o__Bacteroidales	f__[Odoribacteraceae]	g__Odoribacter	Unclassified	5	2	3	5	0	0	3	11	0	0	5	14	1	5	1	0	11	1	0
k__Bacteria	p__Firmicutes	c__Bacilli	o__Gemellales	f__Gemellaceae	Unclassified	Unclassified	1	0	0	1	0	0	0	0	0	1	0	0	0	2	0	1	1	0	1
k__Bacteria	p__Bacteroidetes	c__Bacteroidia	o__Bacteroidales	f__Prevotellaceae	g__Prevotella	Unclassified	27	0	0	0	0	0	43	2	11	0	4	9	24	142	172	27	12	1	2
k__Bacteria	p__Firmicutes	c__Clostridia	o__Clostridiales	f__Ruminococcaceae	g__Ruminococcus	s__bromii	4	0	0	3	46	0	47	0	0	31	0	2	6	6	0	7	3	2	10
k__Bacteria	p__Firmicutes	c__Clostridia	o__Clostridiales	Unclassified	Unclassified	Unclassified	4	1	8	4	1	5	1	4	0	0	2	45	1	1	1	1	1	2	2
k__Bacteria	p__Firmicutes	c__Clostridia	o__Clostridiales	f__Ruminococcaceae	Unclassified	Unclassified	9	8	4	11	0	33	0	0	3	1	0	1	0	31	1	5	14	21	2
k__Bacteria	p__Bacteroidetes	c__Bacteroidia	o__Bacteroidales	f__Prevotellaceae	g__Prevotella	s__copri	15	14	9	2	45	38	0	15	13	37	26	2	15	1	21	37	7	0	6
k__Bacteria	p__Firmicutes	c__Clostridia	o__Clostridiales	f__Ruminococcaceae	Unclassified	Unclassified	2	0	6	10	1	2	3	5	0	1	2	3	3	3	1	0	4	3	2
k__Bacteria	p__Bacteroidetes	c__Bacteroidia	o__Bacteroidales	f__Prevotellaceae	g__Prevotella	Unclassified	1	2	1	4	7	1	8	4	8	5	1	3	2	8	4	1	2	6	4
k__Bacteria	p__Firmicutes	c__Clostridia	o__Clostridiales	f__Lachnospiraceae	Unclassified	Unclassified	3	23	1059	890	19	16	2	0	352	0	38	56	6	248	250	138	1	95	5

Copyright © 2020, Sociedade Brasileira de Genética

**Level_1**	**Level_2**	**Level_3**	**Level_4**	**Level_5**	**Level_6**	**Level_7**	**ss10**	**sn15**	**sn16**	**ss19**	**sn20**	**ss21**	**ss25**	**sn26**	**sn27**	**ss28**	**ss2**	**ss30**	**sn31**	**sn35**	**sn36**	**sn5**	**ss6**	**ss8**	**sn9**
k__Bacteria	p__Firmicutes	c__Clostridia	o__Clostridiales	f__Ruminococcaceae	g__Oscillospira	Unclassified	1	0	9	4	2	5	0	3	3	3	11	9	1	71	11	0	4	0	6
k__Bacteria	p__Bacteroidetes	c__Bacteroidia	o__Bacteroidales	f__S24-7	Unclassified	Unclassified	10	0	10	94	14	4	41	0	24	17	33	19	2	277	57	27	93	17	13
k__Bacteria	p__Firmicutes	c__Clostridia	o__Clostridiales	f__Ruminococcaceae	g__Ruminococcus	Unclassified	1	0	0	22	10	7	39	4	0	0	5	5	2	12	1	24	15	32	5
k__Bacteria	Unclassified	Unclassified	Unclassified	Unclassified	Unclassified	Unclassified	4	3	8	27	0	0	0	0	0	1	0	233	1	0	5	1	7	57	32
k__Bacteria	p__Firmicutes	c__Clostridia	o__Clostridiales	Unclassified	Unclassified	Unclassified	7	0	2	126	0	7	0	103	0	0	0	10	1	1	34	2	1	67	20
k__Bacteria	p__Firmicutes	c__Clostridia	o__Clostridiales	f__Ruminococcaceae	g__Ruminococcus	Unclassified	2	5	10	11	2	1	0	3	3	0	0	67	5	16	2	5	10	8	4
k__Bacteria	p__Actinobacteria	c__Coriobacteriia	o__Coriobacteriales	f__Coriobacteriaceae	Unclassified	Unclassified	2	0	0	1	0	0	0	0	0	1	0	0	0	1	0	0	0	0	0
k__Bacteria	p__Firmicutes	c__Clostridia	o__Clostridiales	f__Ruminococcaceae	g__Oscillospira	Unclassified	9	11	90	69	11	11	158	20	13	13	36	48	17	178	89	12	115	31	28
k__Bacteria	p__Firmicutes	c__Clostridia	o__Clostridiales	f__Lachnospiraceae	Unclassified	Unclassified	1	6	2	0	3	10	0	0	1	4	5	11	1	7	2	1	3	0	0
k__Bacteria	p__Firmicutes	c__Clostridia	o__Clostridiales	f__Ruminococcaceae	g__Ruminococcus	Unclassified	6	17	32	13	3	4	11	24	47	2	6	24	8	18	14	6	55	14	8
k__Bacteria	p__Firmicutes	c__Clostridia	o__Clostridiales	f__Ruminococcaceae	Unclassified	Unclassified	2	1	0	0	0	4	6	0	1	1	0	30	1	0	0	1	4	4	1
k__Bacteria	Unclassified	Unclassified	Unclassified	Unclassified	Unclassified	Unclassified	1	4	40	10	0	1	3	21	0	2	12	1	7	16	2	0	7	2	0
k__Bacteria	p__Bacteroidetes	c__Bacteroidia	o__Bacteroidales	f__[Paraprevotellaceae]	g__CF231	Unclassified	4	298	558	0	0	131	138	0	832	171	117	755	936	0	0	433	656	1193	114
k__Bacteria	p__Bacteroidetes	c__Bacteroidia	o__Bacteroidales	f__Prevotellaceae	g__Prevotella	s__copri	5	0	0	0	8	0	4	3	6	1	2	1	0	0	0	4	14	0	1

Copyright © 2020, Sociedade Brasileira de Genética

Level_1	Level_2	Level_3	Level_4	Level_5	Level_6	Level_7	ss10	sn15	sn16	ss19	sn20	ss21	ss25	sn26	sn27	ss28	ss2	ss30	sn31	sn35	sn36	sn5	ss6	ss8	sn9
k__Bacteria	p__Proteobacteria	Unclassified	Unclassified	Unclassified	Unclassified	Unclassified	1	0	2	0	1	1	1	2	1	1	0	3	4	0	0	1	0	3	1
k__Bacteria	p__Firmicutes	c__Clostridia	o__Clostridiales	f__Lachnospiraceae	Unclassified	Unclassified	3	4	3	1	1	0	0	1	0	0	0	17	0	1	0	1	6	1	0
k__Bacteria	p__Firmicutes	c__Clostridia	o__Clostridiales	f__[Mogibacteriaceae]	Unclassified	Unclassified	6	0	0	0	3	1	2	0	4	4	0	0	0	0	0	0	0	18	2
k__Bacteria	p__Firmicutes	c__Clostridia	o__Clostridiales	f__Lachnospiraceae	Unclassified	Unclassified	8	5	4	3	8	6	0	0	7	0	1	1	1	0	2	0	11	1	6
k__Bacteria	p__Firmicutes	c__Clostridia	o__Clostridiales	f__Ruminococcaceae	g__Oscillospira	Unclassified	2	1	4	1	0	3	0	0	2	1	0	5	0	0	0	0	2	3	0
k__Bacteria	p__Firmicutes	c__Clostridia	o__Clostridiales	f__Ruminococcaceae	Unclassified	Unclassified	2	1	1	4	1	1	0	3	1	3	3	3	3	0	2	0	4	3	0
k__Bacteria	p__Firmicutes	c__Clostridia	o__Clostridiales	f__Ruminococcaceae	g__Ruminococcus	Unclassified	9	6	2	0	0	19	4	11	28	11	30	10	16	0	0	24	19	14	36
k__Bacteria	p__Firmicutes	c__Clostridia	o__Clostridiales	f__Lachnospiraceae	g__Dorea	Unclassified	4	6	0	13	2	1	8	36	0	1	1	3	0	1	1	1	5	0	5
k__Bacteria	p__Tenericutes	c__Mollicutes	o__RF39	Unclassified	Unclassified	Unclassified	2	0	0	0	0	0	40	0	0	0	2	0	0	0	0	0	0	0	0
k__Bacteria	p__Bacteroidetes	c__Bacteroidia	o__Bacteroidales	f__Prevotellaceae	g__Prevotella	s__copri	1	0	2	0	1	0	0	0	1	2	0	0	0	0	0	1	1	0	0
k__Bacteria	Unclassified	Unclassified	Unclassified	Unclassified	Unclassified	Unclassified	13	4	3	2	3	3	8	18	0	1	0	1	5	153	16	6	6	5	0
k__Bacteria	p__Proteobacteria	c__Gammaproteobacteria	o__Pasteurellales	f__Pasteurellaceae	g__Aggregatibacter	Unclassified	1	1	4	1	0	1	0	0	0	2	1	1	2	0	5	4	0	1	2
k__Bacteria	p__Firmicutes	c__Clostridia	o__Clostridiales	f__Ruminococcaceae	g__Oscillospira	Unclassified	9	1	3	7	6	2	4	3	15	9	14	8	3	46	28	25	7	8	20
k__Bacteria	Unclassified	Unclassified	Unclassified	Unclassified	Unclassified	Unclassified	2	0	0	0	1	2	0	0	0	0	0	0	0	0	0	0	0	0	0

Copyright © 2020, Sociedade Brasileira de Genética

**Level_1**	**Level_2**	**Level_3**	**Level_4**	**Level_5**	**Level_6**	**Level_7**	**ss10**	**sn15**	**sn16**	**ss19**	**sn20**	**ss21**	**ss25**	**sn26**	**sn27**	**ss28**	**ss2**	**ss30**	**sn31**	**sn35**	**sn36**	**sn5**	**ss6**	**ss8**	**sn9**
k__Bacteria	p__Firmicutes	c__Clostridia	o__Clostridiales	f__Ruminococcaceae	Unclassified	Unclassified	2	6	0	0	0	0	19	0	0	0	0	3	3	5	4	1	0	0	3
k__Bacteria	p__Firmicutes	c__Clostridia	o__Clostridiales	f__Lachnospiraceae	Unclassified	Unclassified	2	2	1	2	0	2	0	0	0	0	0	0	7	0	0	0	114	2	16
k__Bacteria	p__Firmicutes	c__Clostridia	o__Clostridiales	f__[Mogibacteriaceae]	Unclassified	Unclassified	9	0	1	0	4	5	0	0	18	0	0	1	0	1	4	3	2	5	0
k__Bacteria	Unclassified	Unclassified	Unclassified	Unclassified	Unclassified	Unclassified	2	0	8	0	0	0	0	0	4	0	1	12	1	0	0	0	0	0	0
k__Bacteria	p__Firmicutes	c__Clostridia	o__Clostridiales	Unclassified	Unclassified	Unclassified	5	0	0	0	0	1	0	0	3	0	1	3	0	0	0	2	1	0	0
k__Bacteria	p__Firmicutes	c__Clostridia	o__Clostridiales	f__Lachnospiraceae	Unclassified	Unclassified	2	1	0	0	0	0	0	0	0	0	0	0	0	1	0	0	0	0	0
k__Bacteria	p__Verrucomicrobia	c__Verruco-5	o__WCHB1-41	f__RFP12	Unclassified	Unclassified	4	0	0	0	1	2	0	0	3	0	4	25	9	0	0	0	5	26	7
k__Bacteria	p__Firmicutes	c__Clostridia	o__Clostridiales	f__Lachnospiraceae	Unclassified	Unclassified	2	0	8	1	0	1	3	20	0	1	0	0	1	1	0	0	4	3	0
k__Bacteria	p__Firmicutes	c__Clostridia	o__Clostridiales	f__Ruminococcaceae	Unclassified	Unclassified	5	0	6	3	4	28	1	0	9	0	8	30	4	58	11	17	0	0	1
k__Bacteria	p__Firmicutes	c__Clostridia	o__Clostridiales	Unclassified	Unclassified	Unclassified	4	1	0	3	0	0	0	2	0	0	0	4	0	0	2	0	13	0	0
k__Bacteria	p__Firmicutes	c__Clostridia	o__Clostridiales	f__Lachnospiraceae	Unclassified	Unclassified	2	7	0	8	0	6	0	0	0	0	0	0	0	0	22	0	4	7	3
k__Bacteria	p__Firmicutes	c__Clostridia	o__Clostridiales	f__Ruminococcaceae	Unclassified	Unclassified	5	13	6	19	13	1	20	3	0	11	0	4	40	16	11	1	4	2	17
k__Bacteria	Unclassified	Unclassified	Unclassified	Unclassified	Unclassified	Unclassified	1	0	1	4	1	1	1	0	0	1	1	3	0	2	1	0	1	0	0
k__Bacteria	p__Firmicutes	c__Clostridia	o__Clostridiales	f__Ruminococcaceae	g__Oscillospira	Unclassified	2	0	0	0	1	0	0	0	5	0	0	2	1	0	0	2	0	0	0

Copyright © 2020, Sociedade Brasileira de Genética

Level_1	Level_2	Level_3	Level_4	Level_5	Level_6	Level_7	ss10	sn15	sn16	ss19	sn20	ss21	ss25	sn26	sn27	ss28	ss2	ss30	sn31	sn35	sn36	sn5	ss6	ss8	sn9
k__Bacteria	p__Bacteroidetes	c__Bacteroidia	o__Bacteroidales	f__RF16	Unclassified	Unclassified	18	0	0	0	0	19	0	0	0	0	0	0	78	0	0	65	0	0	0
k__Bacteria	p__Firmicutes	c__Clostridia	o__Clostridiales	f__Dehalobacteriaceae	Unclassified	Unclassified	2	1	0	1	1	0	0	0	1	0	0	1	0	0	2	0	0	1	0
k__Bacteria	p__Actinobacteria	c__Coriobacteriia	o__Coriobacteriales	f__Coriobacteriaceae	g__Adlercreutzia	Unclassified	1	1	0	0	1	0	0	0	0	1	0	0	0	0	0	0	0	0	0
k__Bacteria	p__Firmicutes	c__Clostridia	o__Clostridiales	f__Lachnospiraceae	Unclassified	Unclassified	6	3	0	3	0	1	1	0	0	0	1	0	0	1	0	0	0	2	0
k__Bacteria	Unclassified	Unclassified	Unclassified	Unclassified	Unclassified	Unclassified	1	1	38	24	0	0	4	5	0	1	1	24	0	9	1	1	0	0	2
k__Bacteria	p__Firmicutes	c__Clostridia	o__Clostridiales	Unclassified	Unclassified	Unclassified	5	1	0	4	5	4	0	0	11	3	0	5	0	4	0	0	8	19	3
k__Bacteria	p__Firmicutes	c__Clostridia	o__Clostridiales	Unclassified	Unclassified	Unclassified	3	37	52	9	2	41	94	124	27	324	18	44	228	156	21	45	59	88	65
k__Bacteria	p__Firmicutes	c__Clostridia	o__Clostridiales	Unclassified	Unclassified	Unclassified	9	4	33	48	9	26	15	0	4	12	47	2	8	3	7	8	6	3	17
k__Bacteria	p__Verrucomicrobia	c__Verruco-5	o__WCHB1-41	f__RFP12	Unclassified	Unclassified	1	0	0	0	10	0	0	0	2	0	0	0	4	0	0	0	0	8	0
k__Bacteria	p__Firmicutes	c__Clostridia	o__Clostridiales	Unclassified	Unclassified	Unclassified	3	0	0	5	2	2	1	4	0	0	0	4	1	1	1	1	0	1	1
k__Bacteria	p__Firmicutes	c__Clostridia	o__Clostridiales	f__Ruminococcaceae	Unclassified	Unclassified	3	0	0	0	0	0	0	0	0	0	0	0	0	0	0	0	6	17	0
k__Bacteria	p__Bacteroidetes	c__Bacteroidia	o__Bacteroidales	f__Prevotellaceae	g__Prevotella	s__copri	4	0	0	0	1	2	8	5	5	6	0	2	4	0	0	4	3	0	1
k__Bacteria	p__Actinobacteria	c__Coriobacteriia	o__Coriobacteriales	f__Coriobacteriaceae	Unclassified	Unclassified	1	0	1	2	0	0	0	0	1	0	0	0	4	0	0	0	1	0	0
k__Bacteria	p__Firmicutes	c__Clostridia	o__Clostridiales	f__Lachnospiraceae	g__[Ruminococcus]	s__gnavus	1	0	0	0	0	0	0	0	0	0	0	27	0	0	0	0	9	0	0

Copyright © 2020, Sociedade Brasileira de Genética

Level_1	Level_2	Level_3	Level_4	Level_5	Level_6	Level_7	ss10	sn15	sn16	ss19	sn20	ss21	ss25	sn26	sn27	ss28	ss2	ss30	sn31	sn35	sn36	sn5	ss6	ss8	sn9
k__Bacteria	p__Firmicutes	c__Clostridia	o__Clostridiales	Unclassified	Unclassified	Unclassified	7	0	21	16	11	1	0	4	7	3	3	39	5	19	6	3	4	5	3
k__Archaea	p__Euryarchaeota	c__Thermoplasmata	o__E2	f__[Methanomassiliicoccaceae]	g__vadinCA11	Unclassified	19	7	35	22	20	32	3	5	14	36	18	105	10	24	43	9	25	59	11
k__Bacteria	p__Firmicutes	c__Clostridia	o__Clostridiales	Unclassified	Unclassified	Unclassified	1	0	31	0	2	1	9	9	8	0	27	35	2	28	6	0	1	38	4
k__Bacteria	p__Firmicutes	c__Clostridia	o__Clostridiales	f__Ruminococcaceae	Unclassified	Unclassified	1	1	3	4	1	0	2	2	2	2	0	11	0	0	0	2	2	1	1
k__Bacteria	p__Firmicutes	c__Clostridia	o__Clostridiales	Unclassified	Unclassified	Unclassified	1	0	3	0	0	1	0	0	2	0	0	6	0	1	0	0	0	1	0
k__Bacteria	p__Bacteroidetes	c__Bacteroidia	o__Bacteroidales	f__Bacteroidaceae	g__Bacteroides	Unclassified	1	0	1	118	0	0	0	0	1	0	0	0	0	0	50	0	0	0	0
k__Bacteria	p__Firmicutes	c__Clostridia	o__Clostridiales	f__Ruminococcaceae	g__Oscillospira	Unclassified	2	1	9	2	1	4	6	3	0	0	1	14	1	27	4	0	1	1	2
k__Bacteria	Unclassified	Unclassified	Unclassified	Unclassified	Unclassified	Unclassified	1	0	0	0	1	0	0	0	0	0	0	0	0	0	0	0	0	0	1
k__Bacteria	p__Firmicutes	c__Clostridia	o__Clostridiales	f__Ruminococcaceae	g__Oscillospira	Unclassified	2	1	9	29	3	7	14	1	11	2	2	29	8	12	1	2	8	3	1
k__Bacteria	p__Firmicutes	c__Clostridia	o__Clostridiales	f__Ruminococcaceae	g__Oscillospira	Unclassified	1	0	0	0	0	0	0	0	0	0	2	0	0	5	4	4	0	2	0
k__Bacteria	p__Firmicutes	c__Clostridia	o__Clostridiales	Unclassified	Unclassified	Unclassified	1	0	0	1	0	2	4	8	6	0	3	5	0	0	0	1	6	3	0
k__Bacteria	p__Bacteroidetes	c__Bacteroidia	o__Bacteroidales	f__Prevotellaceae	g__Prevotella	s__copri	5	2	1	62	10	5	19	12	4	12	5	2	8	13	27	13	21	30	13
k__Bacteria	p__Firmicutes	c__Clostridia	o__Clostridiales	f__Ruminococcaceae	Unclassified	Unclassified	1	3	4	2	1	0	0	1178	0	0	1	0	2	0	7	0	2	4	9
k__Bacteria	p__Firmicutes	c__Clostridia	o__Clostridiales	Unclassified	Unclassified	Unclassified	1	0	6	11	0	0	0	11	0	0	3	944	0	1	8	13	0	20	9

Genetics and Molecular Biology 43, 1 (2020)
Copyright © 2020, Sociedade Brasileira de Genética

**Level_1**	**Level_2**	**Level_3**	**Level_4**	**Level_5**	**Level_6**	**Level_7**	**ss10**	**sn15**	**sn16**	**ss19**	**sn20**	**ss21**	**ss25**	**sn26**	**sn27**	**ss28**	**ss2**	**ss30**	**sn31**	**sn35**	**sn36**	**sn5**	**ss6**	**ss8**	**sn9**
k__Bacteria	p__Spirochaetes	c__Spirochaetes	o__Spirochaetales	f__Spirochaetaceae	g__Treponema	Unclassified	3	0	0	23	0	1	1	0	0	1	0	5	0	0	0	0	0	0	0
k__Bacteria	p__Firmicutes	c__Clostridia	o__Clostridiales	f__Lachnospiraceae	g__Lachnospira	Unclassified	7	0	36	0	104	0	0	82	51	69	33	1	4	1	0	1	0	1	0
k__Bacteria	p__Firmicutes	c__Clostridia	o__Clostridiales	f__Ruminococcaceae	g__Oscillospira	Unclassified	3	0	66	13	0	13	72	0	62	10	39	55	10	201	20	19	64	0	9
k__Bacteria	p__Firmicutes	c__Clostridia	o__Clostridiales	f__Lachnospiraceae	g__Blautia	s__obeum	4	8	4	2	33	25	12	16	13	28	3	6	5	8	6	1	2	6	10
k__Bacteria	p__Firmicutes	c__Clostridia	o__Clostridiales	Unclassified	Unclassified	Unclassified	2	0	99	3	0	24	0	282	0	0	0	1	0	0	3	1	0	0	12
k__Bacteria	p__Firmicutes	c__Clostridia	o__Clostridiales	f__Ruminococcaceae	Unclassified	Unclassified	1	0	0	0	9	12	5	0	12	0	1	3	1	5	0	5	4	4	4
k__Bacteria	p__Firmicutes	c__Clostridia	o__Clostridiales	Unclassified	Unclassified	Unclassified	2	24	0	13	24	0	0	0	0	0	42	14	31	8	0	6	3	15	6
k__Bacteria	p__Firmicutes	c__Clostridia	o__Clostridiales	f__Ruminococcaceae	g__Oscillospira	Unclassified	9	7	21	38	6	10	39	30	8	5	6	46	2	43	37	3	23	55	23
k__Bacteria	p__Firmicutes	c__Clostridia	o__Clostridiales	f__Ruminococcaceae	Unclassified	Unclassified	6	6	15	5	1	8	14	11	30	9	0	36	7	20	6	11	14	16	13
k__Bacteria	p__Bacteroidetes	c__Bacteroidia	o__Bacteroidales	f__S24-7	Unclassified	Unclassified	1	0	9	0	0	0	1	22	9	2	0	0	1	0	14	0	2	6	9
k__Bacteria	p__Bacteroidetes	c__Bacteroidia	o__Bacteroidales	f__S24-7	Unclassified	Unclassified	2	0	16	0	1	16	7	7	8	4	1	3	5	1	2	8	16	1	10
k__Bacteria	p__Bacteroidetes	c__Bacteroidia	o__Bacteroidales	f__[Paraprevotellaceae]	g__CF231	Unclassified	2	2	1	2	0	0	2	0	2	1	0	1	0	0	0	2	0	3	0
k__Bacteria	p__Firmicutes	c__Clostridia	o__Clostridiales	f__Lachnospiraceae	Unclassified	Unclassified	3	22	22	0	0	8	48	1	65	16	16	2	4	0	12	4	1	0	0
k__Bacteria	p__Bacteroidetes	c__Bacteroidia	o__Bacteroidales	f__Prevotellaceae	g__Prevotella	Unclassified	1	0	0	0	1	2	0	1	2	3	0	0	2	1	0	0	4	0	0

Copyright © 2020, Sociedade Brasileira de Genética

**Level_1**	**Level_2**	**Level_3**	**Level_4**	**Level_5**	**Level_6**	**Level_7**	**ss10**	**sn15**	**sn16**	**ss19**	**sn20**	**ss21**	**ss25**	**sn26**	**sn27**	**ss28**	**ss2**	**ss30**	**sn31**	**sn35**	**sn36**	**sn5**	**ss6**	**ss8**	**sn9**
k__Bacteria	p__Firmicutes	c__Clostridia	o__Clostridiales	f__Lachnospiraceae	Unclassified	Unclassified	5	1	1	0	2	1	0	0	1	0	1	0	0	2	0	1	0	0	1
k__Bacteria	p__Firmicutes	Unclassified	Unclassified	Unclassified	Unclassified	Unclassified	3	0	0	1	0	0	2	0	0	1	0	0	0	0	0	1	0	1	1
k__Bacteria	p__Firmicutes	c__Clostridia	o__Clostridiales	f__Ruminococcaceae	g__Ruminococcus	Unclassified	2	0	0	0	0	0	0	0	2	0	0	3	0	0	0	1	0	0	1
k__Bacteria	p__Bacteroidetes	c__Bacteroidia	o__Bacteroidales	f__Prevotellaceae	g__Prevotella	s__copri	1	1	0	0	1	1	0	0	1	0	1	1	0	0	3	0	0	0	1
k__Bacteria	p__Firmicutes	c__Clostridia	o__Clostridiales	Unclassified	Unclassified	Unclassified	2	0	9	1	0	0	23	15	27	3	13	3	1	0	18	39	0	49	9
k__Bacteria	p__Firmicutes	c__Clostridia	o__Clostridiales	Unclassified	Unclassified	Unclassified	1	1	5	4	4	0	4	2	0	0	1	7	1	1	0	1	3	1	0
k__Bacteria	p__Firmicutes	c__Clostridia	o__Clostridiales	f__Lachnospiraceae	g__Dorea	Unclassified	4	0	0	0	1	8	0	0	2	0	5	1	0	0	0	7	0	0	0
k__Bacteria	p__Bacteroidetes	c__Bacteroidia	o__Bacteroidales	f__S24-7	Unclassified	Unclassified	2	6	4	0	0	11	0	15	0	0	0	0	0	0	0	0	6	3	42
k__Bacteria	p__Firmicutes	c__Bacilli	o__Lactobacillales	f__Lactobacillaceae	g__Lactobacillus	Unclassified	1	0	0	1	0	1	3	2	0	1	9	0	0	2	1	1	2	0	1
k__Bacteria	p__Firmicutes	c__Clostridia	o__Clostridiales	f__Lachnospiraceae	Unclassified	Unclassified	1	3	1	1	3	0	3	1	2	1	2	0	0	5	3	3	0	1	1
k__Bacteria	p__Firmicutes	c__Clostridia	o__Clostridiales	f__Ruminococcaceae	Unclassified	Unclassified	1	0	6	14	0	0	9	5	0	0	3	22	0	7	3	1	0	5	1
k__Bacteria	p__Firmicutes	c__Clostridia	o__Clostridiales	f__Lachnospiraceae	Unclassified	Unclassified	1	3	74	9	0	2	0	3	2	3	1	97	6	63	22	2	7	2	1
k__Bacteria	p__Bacteroidetes	c__Bacteroidia	o__Bacteroidales	f__Prevotellaceae	g__Prevotella	s__copri	2	1	1	0	1	1	0	0	1	1	0	0	1	0	0	1	0	0	0
k__Bacteria	p__Firmicutes	c__Erysipelotrichi	o__Erysipelotrichales	f__Erysipelotrichaceae	g__p-75-a5	Unclassified	1	1	1	2	0	2	0	0	0	1	0	1	1	1	5	1	0	2	0

Copyright © 2020, Sociedade Brasileira de Genética

Level_1	Level_2	Level_3	Level_4	Level_5	Level_6	Level_7	ss10	sn15	sn16	ss19	sn20	ss21	ss25	sn26	sn27	ss28	ss2	ss30	sn31	sn35	sn36	sn5	ss6	ss8	sn9
k__Bacteria	p__Firmicutes	c__Clostridia	o__Clostridiales	f__Ruminococcaceae	g__Oscillospira	Unclassified	1	0	6	1	0	25	45	0	0	0	3	34	0	29	1	0	0	0	0
k__Bacteria	p__Firmicutes	c__Clostridia	o__Clostridiales	f__Lachnospiraceae	Unclassified	Unclassified	1	0	1	0	0	0	0	4	0	0	0	0	0	0	0	0	1	1	1
k__Bacteria	p__Proteobacteria	c__Alphaproteobacteria	o__RF32	Unclassified	Unclassified	Unclassified	2	4	88	0	2	0	4	0	0	1	26	0	0	2	23	3	0	37	9
k__Bacteria	p__Bacteroidetes	c__Bacteroidia	o__Bacteroidales	f__S24-7	Unclassified	Unclassified	1	0	1	1	1	0	1	1	0	0	0	0	0	0	1	1	2	0	4
k__Bacteria	p__Firmicutes	c__Clostridia	o__Clostridiales	f__Lachnospiraceae	Unclassified	Unclassified	1	6	0	8	1	0	12	5	83	8	317	8	2	6	175	0	0	34	3
k__Bacteria	p__Firmicutes	c__Clostridia	o__Clostridiales	f__Ruminococcaceae	Unclassified	Unclassified	4	0	5	0	0	3	0	0	6	0	3	16	6	24	15	1	9	8	1
k__Bacteria	p__Cyanobacteria	c__4C0d-2	o__YS2	Unclassified	Unclassified	Unclassified	1	0	1	1	0	15	0	57	18	1	0	32	3	0	8	0	65	0	0
k__Bacteria	p__Lentisphaerae	c__[Lentisphaeria]	o__Victivallales	f__Victivallaceae	Unclassified	Unclassified	1	0	1	0	2	7	0	0	4	0	1	3	0	0	3	2	2	3	18
k__Bacteria	p__Firmicutes	c__Clostridia	o__Clostridiales	Unclassified	Unclassified	Unclassified	4	0	0	0	2	24	0	0	8	0	1	3	5	0	0	7	2	0	11
k__Bacteria	p__Firmicutes	c__Clostridia	o__Clostridiales	Unclassified	Unclassified	Unclassified	4	25	2	200	2	29	0	0	1	0	8	13	20	43	1	19	0	5	35
k__Bacteria	p__Firmicutes	c__Clostridia	o__Clostridiales	f__Ruminococcaceae	g__Butyricicoccus	s__pullicaecorum	1	0	0	9	5	1	22	1	1	2	0	15	0	0	1	0	3	9	0
k__Bacteria	p__Proteobacteria	c__Deltaproteobacteria	o__Desulfovibrionales	f__Desulfovibrionaceae	Unclassified	Unclassified	2	1	2	4	2	0	1	0	0	0	1	13	2	7	3	0	0	2	5
k__Bacteria	p__Firmicutes	c__Clostridia	o__Clostridiales	f__Ruminococcaceae	Unclassified	Unclassified	4	4	34	1	85	21	33	15	478	56	10	44	11	855	252	20	24	15	25
k__Bacteria	p__Firmicutes	c__Bacilli	o__Bacillales	f__Staphylococcaceae	g__Staphylococcus	Unclassified	2	0	2	0	0	6	0	1	1	0	0	2	1	3	2	4	1	3	0

Copyright © 2020, Sociedade Brasileira de Genética

Level_1	Level_2	Level_3	Level_4	Level_5	Level_6	Level_7	ss10	sn15	sn16	ss19	sn20	ss21	ss25	sn26	sn27	ss28	ss2	ss30	sn31	sn35	sn36	sn5	ss6	ss8	sn9
k__Bacteria	p__Firmicutes	c__Clostridia	o__Clostridiales	f__Ruminococcaceae	g__Oscillospira	Unclassified	2	0	0	8	2	0	0	0	1	0	3	4	0	1	2	0	1	1	0
k__Bacteria	p__Firmicutes	c__Clostridia	o__Clostridiales	f__Ruminococcaceae	Unclassified	Unclassified	1	0	0	0	0	0	0	0	0	0	0	0	0	1	0	0	0	0	0
k__Bacteria	p__Firmicutes	c__Clostridia	o__Clostridiales	f__Ruminococcaceae	Unclassified	Unclassified	1	0	0	0	0	0	0	2	0	0	0	0	0	1	2	0	0	1	1
k__Bacteria	p__Firmicutes	c__Clostridia	o__Clostridiales	f__Lachnospiraceae	Unclassified	Unclassified	1	7	81	0	0	0	0	0	0	0	0	3	12	0	43	0	0	0	0
k__Bacteria	p__Firmicutes	c__Clostridia	o__Clostridiales	f__Lachnospiraceae	g__Blautia	Unclassified	2	1	0	2	4	2	0	0	0	0	0	0	0	0	0	0	0	0	0
k__Bacteria	p__Actinobacteria	c__Coriobacteriia	o__Coriobacteriales	f__Coriobacteriaceae	Unclassified	Unclassified	1	1	1	0	0	0	0	1	1	0	0	1	0	0	0	0	0	0	0
k__Bacteria	p__Bacteroidetes	c__Bacteroidia	o__Bacteroidales	f__S24-7	Unclassified	Unclassified	1	0	1	0	0	0	0	0	1	0	1	0	1	0	1	0	1	2	0
k__Bacteria	p__Firmicutes	c__Clostridia	o__Clostridiales	f__Veillonellaceae	g__Veillonella	Unclassified	1	1	0	0	0	0	1	1	0	5	1	0	3	3	2	6	4	4	13
k__Bacteria	p__Firmicutes	c__Clostridia	o__Clostridiales	Unclassified	Unclassified	Unclassified	2	0	0	0	0	2	0	0	0	0	0	0	0	0	0	1	0	0	0
k__Bacteria	p__Firmicutes	c__Clostridia	o__Clostridiales	f__Lachnospiraceae	Unclassified	Unclassified	2	0	0	0	1	1	1	1	3	0	3	1	0	1	0	0	0	0	3
k__Bacteria	p__Fibrobacteres	c__Fibrobacteria	o__Fibrobacterales	f__Fibrobacteraceae	g__Fibrobacter	Unclassified	6	79	73	10	6	137	0	0	14	0	8	48	74	0	0	100	19	514	62
k__Bacteria	p__Firmicutes	c__Clostridia	o__Clostridiales	f__Lachnospiraceae	Unclassified	Unclassified	3	3	43	0	4	1	5	9	0	0	0	1	0	0	1	3	1	2	0
k__Bacteria	p__Firmicutes	c__Clostridia	o__Clostridiales	f__Ruminococcaceae	g__Oscillospira	Unclassified	1	0	0	0	1	1	0	0	0	0	0	0	0	0	0	0	1	0	0
k__Bacteria	p__Firmicutes	c__Bacilli	o__Lactobacillales	f__Lactobacillaceae	g__Lactobacillus	Unclassified	1	0	0	0	0	0	0	0	0	0	0	0	0	0	0	0	0	1	0

Copyright © 2020, Sociedade Brasileira de Genética

**Level_1**	**Level_2**	**Level_3**	**Level_4**	**Level_5**	**Level_6**	**Level_7**	**ss10**	**sn15**	**sn16**	**ss19**	**sn20**	**ss21**	**ss25**	**sn26**	**sn27**	**ss28**	**ss2**	**ss30**	**sn31**	**sn35**	**sn36**	**sn5**	**ss6**	**ss8**	**sn9**
k__Bacteria	p__Bacteroidetes	c__Bacteroidia	o__Bacteroidales	f__S24-7	Unclassified	Unclassified	1	0	0	0	1	0	10	0	0	6	0	0	2	0	0	0	6	0	1
k__Bacteria	p__Firmicutes	c__Clostridia	o__Clostridiales	f__Christensenellaceae	Unclassified	Unclassified	1	1	2	0	1	1	2	0	0	2	0	2	1	0	0	0	3	4	0
k__Bacteria	p__Firmicutes	c__Clostridia	o__Clostridiales	f__Ruminococcaceae	g__Oscillospira	Unclassified	3	4	44	21	40	12	56	11	1	6	13	16	11	81	5	5	3	17	9
k__Bacteria	p__Bacteroidetes	c__Bacteroidia	o__Bacteroidales	f__Prevotellaceae	g__Prevotella	s__copri	2	2	0	2	4	0	0	1	1	6	2	2	5	1	3	1	0	0	0
k__Bacteria	p__Firmicutes	c__Clostridia	o__Clostridiales	Unclassified	Unclassified	Unclassified	1	0	0	0	0	0	0	0	0	0	0	1	0	0	1	0	0	0	0
k__Bacteria	p__Bacteroidetes	c__Bacteroidia	o__Bacteroidales	f__Prevotellaceae	g__Prevotella	Unclassified	1	1	2	1	0	1	0	0	1	2	2	1	0	0	1	0	0	0	0
k__Bacteria	p__Firmicutes	c__Clostridia	o__Clostridiales	f__Dehalobacteriaceae	g__Dehalobacterium	Unclassified	1	0	0	1	0	1	0	0	2	0	0	0	0	0	0	0	0	1	0
k__Bacteria	p__Firmicutes	c__Clostridia	o__Clostridiales	Unclassified	Unclassified	Unclassified	2	12	1	4	11	19	179	43	49	4	11	16	8	139	2	27	35	125	23
k__Bacteria	p__Firmicutes	c__Clostridia	o__Clostridiales	f__Ruminococcaceae	g__Ruminococcus	Unclassified	1	0	0	0	0	0	0	0	0	0	0	0	0	1	1	0	0	0	0
k__Bacteria	p__Bacteroidetes	c__Bacteroidia	o__Bacteroidales	f__Prevotellaceae	g__Prevotella	s__copri	2	12	1	4	1	3	12	47	0	0	3	7	13	2	2	31	0	25	36
k__Bacteria	Unclassified	Unclassified	Unclassified	Unclassified	Unclassified	Unclassified	2	4	2	7	0	0	12	258	0	3	3	58	3	12	0	3	5	2	3
k__Bacteria	p__Bacteroidetes	c__Bacteroidia	o__Bacteroidales	f__Bacteroidaceae	g__Bacteroides	s__eggerthii	1	1	2	13	1	2	0	9	2	0	1	4	0	11	5	0	3	1	4
k__Bacteria	p__Firmicutes	c__Clostridia	o__Clostridiales	f__Ruminococcaceae	Unclassified	Unclassified	1	0	0	0	0	1	0	0	1	0	0	3	0	0	0	0	0	0	0
k__Bacteria	p__Firmicutes	c__Clostridia	o__Clostridiales	f__Ruminococcaceae	g__Oscillospira	Unclassified	1	0	0	0	1	0	0	0	2	0	0	3	3	0	0	1	1	3	0

Copyright © 2020, Sociedade Brasileira de Genética

**Level_1**	**Level_2**	**Level_3**	**Level_4**	**Level_5**	**Level_6**	**Level_7**	**ss10**	**sn15**	**sn16**	**ss19**	**sn20**	**ss21**	**ss25**	**sn26**	**sn27**	**ss28**	**ss2**	**ss30**	**sn31**	**sn35**	**sn36**	**sn5**	**ss6**	**ss8**	**sn9**
k__Bacteria	p__Firmicutes	c__Clostridia	o__Clostridiales	f__Ruminococcaceae	Unclassified	Unclassified	1	1	6	32	2	80	4	0	61	0	71	15	5	25	47	61	4	91	6
k__Bacteria	p__Firmicutes	c__Clostridia	o__Clostridiales	f__Ruminococcaceae	g__Oscillospira	Unclassified	2	0	3	5	0	0	1	0	0	0	0	1	0	0	0	0	1	2	0
k__Bacteria	p__Firmicutes	c__Clostridia	o__Clostridiales	f__Lachnospiraceae	Unclassified	Unclassified	1	1	16	8	3	0	2	6	0	3	1	34	1	15	3	0	2	2	3
k__Bacteria	p__Actinobacteria	c__Coriobacteriia	o__Coriobacteriales	f__Coriobacteriaceae	Unclassified	Unclassified	2	2	0	0	0	2	0	0	5	0	1	1	1	0	1	1	0	2	1
k__Bacteria	p__Firmicutes	c__Clostridia	o__Clostridiales	f__Ruminococcaceae	g__Oscillospira	Unclassified	3	1	1	9	0	3	13	9	8	1	1	0	7	45	0	6	11	0	0
k__Bacteria	p__Firmicutes	c__Clostridia	o__Clostridiales	Unclassified	Unclassified	Unclassified	2	0	2	0	0	2	22	0	26	0	26	0	0	0	6	8	37	87	13
k__Bacteria	p__Firmicutes	c__Bacilli	o__Turicibacterales	f__Turicibacteraceae	g__Turicibacter	Unclassified	2	4	0	4	0	0	0	1	0	0	0	2	2	0	0	0	0	0	0
k__Bacteria	p__Firmicutes	c__Clostridia	o__Clostridiales	Unclassified	Unclassified	Unclassified	1	1	38	0	7	0	22	3	1	0	0	3	0	43	8	1	0	5	0
k__Bacteria	p__Firmicutes	c__Clostridia	o__Clostridiales	Unclassified	Unclassified	Unclassified	1	5	2	0	0	0	5	0	0	0	0	4	0	16	0	2	10	28	0
k__Bacteria	p__Firmicutes	c__Clostridia	o__Clostridiales	f__Lachnospiraceae	Unclassified	Unclassified	2	28	1	14	3	3	3	5	1	7	21	156	1	43	5	7	38	23	2
k__Bacteria	p__TM7	c__TM7-3	o__CW040	f__F16	Unclassified	Unclassified	1	0	4	1	0	2	8	1	1	1	3	0	2	4	6	1	3	5	2
k__Bacteria	Unclassified	Unclassified	Unclassified	Unclassified	Unclassified	Unclassified	1	0	1	2	0	1	1	0	2	0	2	1	3	2	0	0	1	3	0
k__Bacteria	p__Firmicutes	c__Clostridia	o__Clostridiales	f__Ruminococcaceae	Unclassified	Unclassified	1	3	0	0	1	0	1	0	0	0	0	0	0	0	1	0	2	0	2
k__Bacteria	p__Firmicutes	c__Clostridia	o__Clostridiales	f__Lachnospiraceae	g__Dorea	Unclassified	1	0	8	0	2	0	1	3	2	1	1	0	2	1	0	0	2	1	3

Copyright © 2020, Sociedade Brasileira de Genética

Level_1	Level_2	Level_3	Level_4	Level_5	Level_6	Level_7	ss10	sn15	sn16	ss19	sn20	ss21	ss25	sn26	sn27	ss28	ss2	ss30	sn31	sn35	sn36	sn5	ss6	ss8	sn9
k__Bacteria	p__Bacteroidetes	c__Bacteroidia	o__Bacteroidales	f__Prevotellaceae	g__Prevotella	Unclassified	1	0	1	0	0	1	2	1	0	2	1	2	0	2	0	0	1	0	0
k__Bacteria	p__Firmicutes	c__Clostridia	o__Clostridiales	f__Lachnospiraceae	Unclassified	Unclassified	1	1	1	0	0	0	0	1	0	0	0	404	1	56	0	0	0	1	0
k__Bacteria	p__Firmicutes	c__Clostridia	o__Clostridiales	f__Lachnospiraceae	Unclassified	Unclassified	1	108	0	1	0	0	1	0	0	0	17	0	10	1	119	216	0	651	38
k__Bacteria	p__Firmicutes	c__Clostridia	o__Clostridiales	f__Ruminococcaceae	g__Ruminococcus	Unclassified	1	0	0	10	2	3	3	3	0	2	5	24	0	5	0	1	4	4	3
k__Bacteria	p__Bacteroidetes	c__Bacteroidia	o__Bacteroidales	f__Prevotellaceae	g__Prevotella	Unclassified	2	1	1	3	1	0	0	2	3	0	2	0	1	2	2	1	4	1	0
k__Bacteria	p__Firmicutes	c__Clostridia	o__Clostridiales	f__Lachnospiraceae	Unclassified	Unclassified	1	0	32	44	0	10	0	0	0	1	16	426	7	93	1	0	8	0	9
k__Bacteria	p__Bacteroidetes	c__Bacteroidia	o__Bacteroidales	f__[Paraprevotellaceae]	g__CF231	Unclassified	1	1	2	0	958	287	92	0	3	1	0	4	313	0	2	52	1053	10	0
k__Bacteria	p__Firmicutes	c__Erysipelotrichi	o__Erysipelotrichales	f__Erysipelotrichaceae	Unclassified	Unclassified	1	2	3	5	11	1	7	0	0	2	1	0	1	0	0	6	0	0	11
k__Bacteria	p__Firmicutes	c__Clostridia	o__Clostridiales	f__Ruminococcaceae	Unclassified	Unclassified	2	1	10	15	2	5	3	7	1	3	4	37	5	23	2	1	0	5	0
k__Bacteria	p__Firmicutes	c__Clostridia	o__Clostridiales	f__Lachnospiraceae	Unclassified	Unclassified	1	0	0	0	0	0	0	0	1	0	0	0	0	0	0	0	0	0	0
k__Bacteria	p__Firmicutes	c__Clostridia	o__Clostridiales	f__Lachnospiraceae	Unclassified	Unclassified	1	2	1	23	3	2	2	4	0	1	3	24	2	2	2	4	3	10	1
k__Bacteria	p__Firmicutes	c__Clostridia	o__Clostridiales	f__Ruminococcaceae	Unclassified	Unclassified	1	0	3	1	0	0	0	0	1	0	0	2	0	6	0	1	1	0	0
k__Bacteria	p__Bacteroidetes	c__Bacteroidia	o__Bacteroidales	f__Prevotellaceae	g__Prevotella	s__copri	1	2	1	1	2	7	0	0	0	5	0	1	0	1	3	0	0	0	0
k__Bacteria	p__Firmicutes	c__Clostridia	o__Clostridiales	f__Lachnospiraceae	Unclassified	Unclassified	1	4	23	29	1	1	1	0	0	1	1	3	0	5	24	0	1	3	0

Copyright © 2020, Sociedade Brasileira de Genética

Level_1	Level_2	Level_3	Level_4	Level_5	Level_6	Level_7	ss10	sn15	sn16	ss19	sn20	ss21	ss25	sn26	sn27	ss28	ss2	ss30	sn31	sn35	sn36	sn5	ss6	ss8	sn9
k__Bacteria	p__Firmicutes	c__Clostridia	o__Clostridiales	f__Ruminococcaceae	Unclassified	Unclassified	1	0	0	1	1	0	3	0	3	0	0	16	1	3	0	0	0	0	0
k__Bacteria	p__Firmicutes	c__Clostridia	o__Clostridiales	f__Ruminococcaceae	Unclassified	Unclassified	1	0	9	3	2	1	2	0	2	1	5	8	2	3	2	0	1	1	0
k__Bacteria	p__Firmicutes	c__Clostridia	o__Clostridiales	Unclassified	Unclassified	Unclassified	1	23	15	10	1	0	14	19	1	1	0	33	1	24	3	0	1	3	1
k__Bacteria	p__Firmicutes	c__Clostridia	o__Clostridiales	f__Veillonellaceae	Unclassified	Unclassified	1	23	140	0	66	42	33	30	23	76	14	109	13	273	20	64	62	1	0
k__Bacteria	p__Firmicutes	c__Clostridia	o__Clostridiales	f__Lachnospiraceae	g__Anaerostipes	Unclassified	1	3	0	1	4	15	4	0	10	0	1	3	1	2	0	1	11	7	3
k__Bacteria	p__Firmicutes	c__Clostridia	o__Clostridiales	Unclassified	Unclassified	Unclassified	1	3	0	0	1	8	5	0	5	0	0	0	0	0	0	3	2	0	0
k__Bacteria	p__Firmicutes	c__Clostridia	o__Clostridiales	f__Christensenellaceae	Unclassified	Unclassified	1	0	0	1	0	0	0	0	0	0	0	0	0	0	0	0	1	0	0
k__Bacteria	p__Bacteroidetes	c__Bacteroidia	o__Bacteroidales	f__S24-7	Unclassified	Unclassified	1	2	10	0	0	38	3	13	0	2	0	0	7	6	5	0	0	16	33
k__Bacteria	p__Firmicutes	c__Clostridia	o__Clostridiales	f__Ruminococcaceae	Unclassified	Unclassified	1	0	21	71	14	9	11	2	3	8	13	54	4	3	12	1	5	2	1
k__Bacteria	p__Firmicutes	c__Clostridia	o__Clostridiales	f__Ruminococcaceae	g__Oscillospira	Unclassified	1	0	0	8	4	0	3	2	3	0	0	8	1	2	2	0	0	3	0
k__Bacteria	p__Proteobacteria	c__Alphaproteobacteria	o__Rickettsiales	f__mitochondria	Unclassified	Unclassified	1	0	0	0	1	0	0	0	1	2	0	0	0	0	1	1	0	1	2
k__Bacteria	p__Firmicutes	c__Clostridia	o__Clostridiales	f__Ruminococcaceae	Unclassified	Unclassified	2	1	6	0	0	0	0	0	1	1	1	5	0	5	0	0	1	0	0
k__Bacteria	p__Bacteroidetes	c__Bacteroidia	o__Bacteroidales	f__Prevotellaceae	g__Prevotella	s__copri	1	0	191	1	48	37	0	1	41	1233	10	0	13	0	72	0	0	0	0
k__Bacteria	p__Firmicutes	c__Clostridia	o__Clostridiales	Unclassified	Unclassified	Unclassified	1	4	0	3	0	0	2	1	0	0	0	1	0	4	0	0	0	0	0

Copyright © 2020, Sociedade Brasileira de Genética

**Level_1**	**Level_2**	**Level_3**	**Level_4**	**Level_5**	**Level_6**	**Level_7**	**ss10**	**sn15**	**sn16**	**ss19**	**sn20**	**ss21**	**ss25**	**sn26**	**sn27**	**ss28**	**ss2**	**ss30**	**sn31**	**sn35**	**sn36**	**sn5**	**ss6**	**ss8**	**sn9**
k__Bacteria	p__Firmicutes	c__Clostridia	o__Clostridiales	Unclassified	Unclassified	Unclassified	1	5	0	3	0	21	14	0	49	19	313	33	6	1	122	14	6	8	6
k__Bacteria	p__Bacteroidetes	c__Bacteroidia	o__Bacteroidales	f__Prevotellaceae	g__Prevotella	Unclassified	1	0	0	2	4	0	0	3	0	0	2	1	0	0	2	1	2	1	2
k__Bacteria	p__Firmicutes	c__Clostridia	o__Clostridiales	f__Ruminococcaceae	g__Oscillospira	Unclassified	2	1	4	6	2	0	21	7	1	1	0	5	1	10	0	0	6	1	2
k__Bacteria	p__Firmicutes	c__Clostridia	o__Clostridiales	f__Ruminococcaceae	g__Ruminococcus	Unclassified	1	19	1	0	75	2	21	46	48	41	0	0	4	0	18	23	0	0	2
k__Bacteria	p__Tenericutes	c__Mollicutes	o__RF39	Unclassified	Unclassified	Unclassified	1	3	13	1	2	0	62	1	4	0	7	27	0	9	4	0	1	4	0
k__Bacteria	p__Firmicutes	c__Clostridia	o__Clostridiales	f__Ruminococcaceae	Unclassified	Unclassified	1	0	0	3	0	0	0	2	0	0	0	7	0	0	0	0	1	0	0
k__Bacteria	p__Bacteroidetes	c__Bacteroidia	o__Bacteroidales	f__Prevotellaceae	g__Prevotella	s__copri	1	0	0	0	3	1	0	0	1	1	3	0	0	0	0	0	0	0	1
k__Bacteria	p__Firmicutes	c__Clostridia	o__Clostridiales	f__Christensenellaceae	Unclassified	Unclassified	1	0	0	0	0	1	0	0	0	0	0	2	0	0	0	1	0	2	3
k__Bacteria	p__Firmicutes	c__Clostridia	o__Clostridiales	f__Lachnospiraceae	Unclassified	Unclassified	0	145	23	0	0	161	120	23	0	49	3	144	20	377	3	0	2	0	1
k__Bacteria	p__Firmicutes	c__Clostridia	o__Clostridiales	f__Lachnospiraceae	Unclassified	Unclassified	0	88	166	86	0	4	0	0	0	25	222	10	12	24	25	77	1	1	1
k__Bacteria	p__Bacteroidetes	c__Bacteroidia	o__Bacteroidales	f__S24-7	Unclassified	Unclassified	0	16	25	28	0	0	13	89	0	6	6	0	0	0	74	0	0	98	28
k__Bacteria	p__Firmicutes	c__Clostridia	o__Clostridiales	Unclassified	Unclassified	Unclassified	0	24	221	0	0	17	16	0	0	9	0	164	5	15	102	0	35	1	0
k__Bacteria	p__Firmicutes	c__Clostridia	o__Clostridiales	f__Ruminococcaceae	Unclassified	Unclassified	0	7	29	21	342	5	304	199	9	180	6	3	207	0	223	94	8	3	12
k__Bacteria	p__Bacteroidetes	c__Bacteroidia	o__Bacteroidales	f__Prevotellaceae	g__Prevotella	Unclassified	0	651	111	0	0	672	1	0	359	1	568	250	1022	0	2039	3925	0	12	3539

Copyright © 2020, Sociedade Brasileira de Genética

**Level_1**	**Level_2**	**Level_3**	**Level_4**	**Level_5**	**Level_6**	**Level_7**	**ss10**	**sn15**	**sn16**	**ss19**	**sn20**	**ss21**	**ss25**	**sn26**	**sn27**	**ss28**	**ss2**	**ss30**	**sn31**	**sn35**	**sn36**	**sn5**	**ss6**	**ss8**	**sn9**
k__Bacteria	p__Firmicutes	c__Clostridia	o__Clostridiales	Unclassified	Unclassified	Unclassified	0	21	0	26	0	0	0	0	0	0	0	0	0	0	0	0	0	0	0
k__Bacteria	p__Firmicutes	c__Clostridia	o__Clostridiales	f__Ruminococcaceae	g__Oscillospira	Unclassified	0	19	73	3	0	76	0	1	2	1	0	14	47	11	43	58	94	34	8
k__Bacteria	p__Firmicutes	c__Clostridia	o__Clostridiales	f__Ruminococcaceae	g__Faecalibacterium	s__prausnitzii	0	19	18	0	0	7	0	0	34	4	28	0	0	0	0	378	0	0	0
k__Bacteria	p__Firmicutes	c__Clostridia	o__Clostridiales	f__Lachnospiraceae	g__Oribacterium	Unclassified	0	60	45	0	6	161	0	0	85	72	42	0	3	0	0	100	0	0	32
k__Bacteria	p__Firmicutes	c__Clostridia	o__Clostridiales	f__Lachnospiraceae	Unclassified	Unclassified	0	133	0	0	0	0	0	0	0	0	0	0	37	23	13	4	161	2	222
k__Bacteria	Unclassified	Unclassified	Unclassified	Unclassified	Unclassified	Unclassified	0	57	3	0	0	0	0	0	4	0	2	0	0	0	0	0	0	11	0
k__Bacteria	p__Bacteroidetes	c__Bacteroidia	o__Bacteroidales	f__S24-7	Unclassified	Unclassified	0	22	35	0	0	0	1	30	71	0	3	1	10	0	0	0	0	0	0
k__Bacteria	p__Firmicutes	c__Clostridia	o__Clostridiales	Unclassified	Unclassified	Unclassified	0	8	3	1	6	1	15	1	2	2	2	2	2	4	4	1	2	5	3
k__Bacteria	p__Spirochaetes	c__Spirochaetes	o__M2PT2-76	Unclassified	Unclassified	Unclassified	0	2	0	0	0	5	0	0	0	0	1	7	0	0	0	0	0	1	1
k__Bacteria	p__Firmicutes	c__Clostridia	o__Clostridiales	Unclassified	Unclassified	Unclassified	0	21	47	35	0	0	0	1	0	1	0	3	5	0	27	2	1	0	2
k__Bacteria	p__Firmicutes	c__Clostridia	o__Clostridiales	Unclassified	Unclassified	Unclassified	0	21	1	220	0	0	0	0	0	0	0	44	0	2	0	118	0	0	0
k__Bacteria	p__Firmicutes	c__Clostridia	o__Clostridiales	f__Lachnospiraceae	Unclassified	Unclassified	0	1	3	6	1	1	0	2	0	0	2	7	1	19	0	0	2	0	0
k__Bacteria	p__Firmicutes	c__Clostridia	o__Clostridiales	Unclassified	Unclassified	Unclassified	0	62	0	0	32	0	7	7	92	39	1	16	0	108	8	1	0	0	0
k__Bacteria	p__Firmicutes	c__Clostridia	o__Clostridiales	Unclassified	Unclassified	Unclassified	0	76	0	0	0	29	0	0	5	0	0	0	0	0	0	141	0	0	9

Copyright © 2020, Sociedade Brasileira de Genética

Level_1	Level_2	Level_3	Level_4	Level_5	Level_6	Level_7	ss10	sn15	sn16	ss19	sn20	ss21	ss25	sn26	sn27	ss28	ss2	ss30	sn31	sn35	sn36	sn5	ss6	ss8	sn9
k__Bacteria	p__Bacteroidetes	c__Bacteroidia	o__Bacteroidales	f__S24-7	Unclassified	Unclassified	0	1	7	0	0	0	1	5	6	0	1	1	6	0	9	0	0	1	0
k__Bacteria	p__Firmicutes	c__Clostridia	o__Clostridiales	f__Lachnospiraceae	g__Coprococcus	Unclassified	0	5	0	0	0	0	0	0	0	0	0	0	3	0	0	0	1	0	1
k__Bacteria	p__Firmicutes	c__Clostridia	o__Clostridiales	f__Lachnospiraceae	Unclassified	Unclassified	0	2	0	5	0	6	7	0	0	0	1	1	0	30	3	1	0	3	14
k__Bacteria	p__Firmicutes	c__Clostridia	o__Clostridiales	f__Lachnospiraceae	Unclassified	Unclassified	0	24	0	51	41	0	0	0	0	0	2	0	0	0	0	3	0	0	9
k__Bacteria	p__Proteobacteria	c__Gammaproteobacteria	o__Aeromonadales	f__Succinivibrionaceae	Unclassified	Unclassified	0	50	69	0	0	53	0	1	84	0	72	49	17	503	102	63	0	153	0
k__Bacteria	p__Firmicutes	c__Clostridia	o__Clostridiales	Unclassified	Unclassified	Unclassified	0	9	0	0	0	0	0	3	0	0	0	0	0	0	0	0	7	0	0
k__Bacteria	p__Firmicutes	c__Clostridia	o__Clostridiales	Unclassified	Unclassified	Unclassified	0	1	0	1	0	4	0	0	12	6	10	819	17	352	36	0	2	0	0
k__Bacteria	p__Firmicutes	c__Clostridia	o__Clostridiales	f__Lachnospiraceae	Unclassified	Unclassified	0	14	37	25	0	20	46	2	163	4	13	0	8	34	55	78	0	2	121
k__Bacteria	p__Firmicutes	c__Clostridia	o__Clostridiales	f__Lachnospiraceae	Unclassified	Unclassified	0	6	0	0	0	0	1	0	0	0	1	7	0	0	0	0	0	0	0
k__Bacteria	p__Firmicutes	c__Clostridia	o__Clostridiales	f__Lachnospiraceae	Unclassified	Unclassified	0	11	1	30	0	2	0	0	0	0	0	0	0	0	0	0	4	4	0
k__Bacteria	p__Bacteroidetes	c__Bacteroidia	o__Bacteroidales	f__S24-7	Unclassified	Unclassified	0	40	53	0	39	70	110	621	133	25	16	51	61	0	49	62	0	107	50
k__Bacteria	p__Firmicutes	c__Clostridia	o__Clostridiales	f__Lachnospiraceae	Unclassified	Unclassified	0	10	0	0	0	0	0	0	87	109	53	0	0	34	3	0	0	0	0
k__Bacteria	p__Firmicutes	c__Clostridia	o__Clostridiales	f__Lachnospiraceae	Unclassified	Unclassified	0	5	14	21	5	1	3	0	1	6	5	14	0	16	2	1	2	0	2
k__Bacteria	p__Firmicutes	c__Clostridia	o__Clostridiales	Unclassified	Unclassified	Unclassified	0	5	11	30	0	1	0	0	0	0	1	11	1	0	1	2	0	3	3

Copyright © 2020, Sociedade Brasileira de Genética

**Level_1**	**Level_2**	**Level_3**	**Level_4**	**Level_5**	**Level_6**	**Level_7**	**ss10**	**sn15**	**sn16**	**ss19**	**sn20**	**ss21**	**ss25**	**sn26**	**sn27**	**ss28**	**ss2**	**ss30**	**sn31**	**sn35**	**sn36**	**sn5**	**ss6**	**ss8**	**sn9**
k__Bacteria	p__Firmicutes	c__Erysipelotrichi	o__Erysipelotrichales	f__Erysipelotrichaceae	g__p-75-a5	Unclassified	0	1	1	0	1	3	4	3	6	2	0	6	2	4	4	0	3	1	1
k__Bacteria	p__Bacteroidetes	c__Bacteroidia	o__Bacteroidales	f__S24-7	Unclassified	Unclassified	0	1	0	69	1	0	21	66	0	3	0	0	0	0	0	0	0	0	0
k__Bacteria	p__Firmicutes	c__Clostridia	o__Clostridiales	f__Ruminococcaceae	Unclassified	Unclassified	0	1	3	8	0	0	1	1	2	0	0	16	2	0	0	0	2	2	0
k__Bacteria	p__Proteobacteria	c__Gammaproteobacteria	o__Enterobacteriales	f__Enterobacteriaceae	g__Escherichia	s__coli	0	1	0	7	0	0	0	0	5	0	0	0	0	0	0	0	0	0	1
k__Bacteria	p__Firmicutes	c__Clostridia	o__Clostridiales	f__Lachnospiraceae	g__[Ruminococcus]	s__gnavus	0	5	0	2	0	0	0	0	0	0	0	0	0	0	0	0	0	2	33
k__Bacteria	p__Firmicutes	c__Clostridia	o__Clostridiales	Unclassified	Unclassified	Unclassified	0	4	32	1044	3	0	92	0	4	1	5	2	0	13	23	1	3	166	8
k__Bacteria	p__Firmicutes	c__Clostridia	o__Clostridiales	f__Lachnospiraceae	Unclassified	Unclassified	0	1	2	5	0	1	2	0	0	1	2	2	1	5	0	1	1	0	0
k__Bacteria	p__Firmicutes	c__Clostridia	o__Clostridiales	Unclassified	Unclassified	Unclassified	0	3	18	7	2	0	0	1	5	0	10	0	7	7	3	3	3	0	1
k__Bacteria	p__Firmicutes	c__Erysipelotrichi	o__Erysipelotrichales	f__Erysipelotrichaceae	Unclassified	Unclassified	0	7	0	1	0	1	0	0	4	4	3	0	1	2	0	0	0	0	0
k__Bacteria	p__Firmicutes	c__Clostridia	o__Clostridiales	Unclassified	Unclassified	Unclassified	0	11	10	0	0	0	0	0	0	31	14	0	5	0	13	18	4	7	0
k__Bacteria	p__Bacteroidetes	c__Bacteroidia	o__Bacteroidales	f__Prevotellaceae	g__Prevotella	Unclassified	0	1	0	0	0	0	0	0	0	1	1	1	1	0	0	0	2	0	1
k__Bacteria	p__Proteobacteria	c__Betaproteobacteria	o__Burkholderiales	f__Alcaligenaceae	g__Sutterella	Unclassified	0	11	2	0	0	21	3	5	15	16	1	37	2	31	3	14	4	1	1
k__Bacteria	p__Proteobacteria	c__Betaproteobacteria	o__Burkholderiales	f__Alcaligenaceae	g__Sutterella	Unclassified	0	3	3	0	0	18	3	16	31	12	2	20	21	3	2	25	10	8	2
k__Bacteria	p__Firmicutes	c__Clostridia	o__Clostridiales	Unclassified	Unclassified	Unclassified	0	6	0	0	0	0	15	0	0	0	0	0	0	0	0	0	4	1	1

Copyright © 2020, Sociedade Brasileira de Genética

Level_1	Level_2	Level_3	Level_4	Level_5	Level_6	Level_7	ss10	sn15	sn16	ss19	sn20	ss21	ss25	sn26	sn27	ss28	ss2	ss30	sn31	sn35	sn36	sn5	ss6	ss8	sn9
k__Bacteria	p__Firmicutes	c__Clostridia	o__Clostridiales	f__Ruminococcaceae	Unclassified	Unclassified	0	2	1	1	2	0	2	0	3	0	1	4	0	1	2	0	4	1	0
k__Bacteria	p__Proteobacteria	c__Alphaproteobacteria	o__RF32	Unclassified	Unclassified	Unclassified	0	9	0	0	0	0	0	0	0	0	0	0	0	0	0	0	0	0	0
k__Bacteria	p__Firmicutes	c__Clostridia	o__Clostridiales	Unclassified	Unclassified	Unclassified	0	7	131	851	0	1	22	54	9	5	7	216	8	264	45	2	286	56	2
k__Bacteria	p__Firmicutes	c__Clostridia	o__Clostridiales	Unclassified	Unclassified	Unclassified	0	9	0	40	1	2	0	0	3	0	0	0	3	0	0	3	5	0	0
k__Bacteria	p__Bacteroidetes	c__Bacteroidia	o__Bacteroidales	f__S24-7	Unclassified	Unclassified	0	2	0	0	1	0	0	4	0	0	1	0	0	0	0	0	0	2	1
k__Bacteria	p__Firmicutes	c__Bacilli	o__Lactobacillales	f__Streptococcaceae	g__Streptococcus	Unclassified	0	2	0	1	5	2	1	2	29	3	1	0	1	6	1	5	2	2	9
k__Bacteria	p__Bacteroidetes	c__Bacteroidia	o__Bacteroidales	Unclassified	Unclassified	Unclassified	0	7	12	1	0	0	10	0	6	0	2	11	0	0	2	0	22	0	0
k__Bacteria	Unclassified	Unclassified	Unclassified	Unclassified	Unclassified	Unclassified	0	2	0	0	0	0	0	0	0	0	0	0	0	0	0	0	0	0	0
k__Bacteria	p__Bacteroidetes	c__Bacteroidia	o__Bacteroidales	f__[Paraprevotellaceae]	Unclassified	Unclassified	0	6	14	1	0	1	14	12	8	8	10	28	11	96	13	17	13	5	16
k__Bacteria	p__Bacteroidetes	c__Bacteroidia	o__Bacteroidales	f__[Odoribacteraceae]	g__Butyricimonas	Unclassified	0	3	0	22	7	2	4	0	4	7	2	10	3	18	7	4	2	1	3
k__Bacteria	p__Firmicutes	c__Clostridia	o__Clostridiales	f__Lachnospiraceae	Unclassified	Unclassified	0	6	174	268	0	65	40	65	16	15	3	2282	52	195	163	0	40	1	0
k__Bacteria	p__Firmicutes	c__Clostridia	o__Clostridiales	f__Ruminococcaceae	Unclassified	Unclassified	0	4	1	1	0	0	0	0	3	3	5	1	1	7	3	2	6	2	0
k__Bacteria	p__Firmicutes	c__Clostridia	o__Clostridiales	f__Ruminococcaceae	g__Ruminococcus	Unclassified	0	8	3	0	1	14	0	0	1	0	1	35	3	59	1	4	3	8	5
k__Bacteria	p__Firmicutes	c__Clostridia	o__Clostridiales	Unclassified	Unclassified	Unclassified	0	3	10	0	3	3	0	1	1	0	6	12	0	21	5	1	2	1	2

Copyright © 2020, Sociedade Brasileira de Genética

**Level_1**	**Level_2**	**Level_3**	**Level_4**	**Level_5**	**Level_6**	**Level_7**	**ss10**	**sn15**	**sn16**	**ss19**	**sn20**	**ss21**	**ss25**	**sn26**	**sn27**	**ss28**	**ss2**	**ss30**	**sn31**	**sn35**	**sn36**	**sn5**	**ss6**	**ss8**	**sn9**
k__Bacteria	p__Firmicutes	c__Clostridia	o__Clostridiales	Unclassified	Unclassified	Unclassified	0	2	6	0	0	1	0	0	0	12	0	180	31	0	9	0	0	0	0
k__Bacteria	p__Firmicutes	c__Clostridia	o__Clostridiales	f__Ruminococcaceae	Unclassified	Unclassified	0	7	18	2	0	0	7	24	0	4	1	0	0	0	0	0	2	0	1
k__Bacteria	p__Firmicutes	c__Clostridia	o__Clostridiales	f__Lachnospiraceae	Unclassified	Unclassified	0	3	1	13	0	2	0	0	0	0	0	0	0	1	0	0	1	0	0
k__Bacteria	p__Bacteroidetes	c__Bacteroidia	o__Bacteroidales	f__S24-7	Unclassified	Unclassified	0	4	2	0	0	10	6	87	0	0	3	0	0	1	12	0	0	22	8
k__Bacteria	p__Bacteroidetes	c__Bacteroidia	o__Bacteroidales	Unclassified	Unclassified	Unclassified	0	2	0	0	0	0	1	0	0	0	0	2	1	2	1	0	1	0	2
k__Bacteria	p__Firmicutes	c__Clostridia	o__Clostridiales	f__Ruminococcaceae	Unclassified	Unclassified	0	1	0	0	0	2	0	1	1	1	0	2	1	11	0	0	1	0	0
k__Bacteria	p__Firmicutes	c__Clostridia	o__Clostridiales	Unclassified	Unclassified	Unclassified	0	2	4	0	1	6	9	0	4	0	0	5	3	1	2	0	6	1	1
k__Bacteria	p__Firmicutes	c__Clostridia	o__Clostridiales	f__Lachnospiraceae	Unclassified	Unclassified	0	1	0	1	0	0	0	0	0	0	0	0	0	0	0	0	1	0	0
k__Bacteria	p__Firmicutes	c__Clostridia	o__Clostridiales	f__Ruminococcaceae	g__Ruminococcus	Unclassified	0	6	1	0	0	0	0	0	0	0	3	41	0	47	9	0	0	2	2
k__Bacteria	Unclassified	Unclassified	Unclassified	Unclassified	Unclassified	Unclassified	0	2	0	0	0	0	0	5	0	0	0	0	0	0	0	0	1	0	0
k__Bacteria	p__Firmicutes	c__Clostridia	o__Clostridiales	f__Lachnospiraceae	Unclassified	Unclassified	0	1	2	0	0	1	0	0	0	0	0	7	0	6	0	0	0	1	0
k__Bacteria	p__Firmicutes	c__Clostridia	o__Clostridiales	f__Lachnospiraceae	Unclassified	Unclassified	0	3	0	0	0	0	0	25	0	0	0	28	0	0	0	0	0	2	0
k__Bacteria	p__Firmicutes	c__Clostridia	o__Clostridiales	f__Lachnospiraceae	Unclassified	Unclassified	0	2	0	0	0	0	0	0	0	0	0	0	0	5	0	12	0	0	6
k__Bacteria	p__Firmicutes	c__Clostridia	o__Clostridiales	Unclassified	Unclassified	Unclassified	0	5	0	0	0	0	0	0	6	23	0	0	0	0	0	0	3	0	0

Copyright © 2020, Sociedade Brasileira de Genética

Level_1	Level_2	Level_3	Level_4	Level_5	Level_6	Level_7	ss10	sn15	sn16	ss19	sn20	ss21	ss25	sn26	sn27	ss28	ss2	ss30	sn31	sn35	sn36	sn5	ss6	ss8	sn9
k__Bacteria	p__Firmicutes	c__Clostridia	o__Clostridiales	f__Lachnospiraceae	g__Coprococcus	Unclassified	0	8	2	0	0	4	0	0	7	3	3	0	0	1	0	4	0	0	0
k__Bacteria	p__Firmicutes	c__Clostridia	o__Clostridiales	Unclassified	Unclassified	Unclassified	0	1	0	0	0	0	7	0	0	1	1	2	2	3	0	1	4	8	5
k__Bacteria	p__Firmicutes	c__Clostridia	o__Clostridiales	f__Lachnospiraceae	g__[Ruminococcus]	s__gnavus	0	3	0	1	0	1	0	0	0	0	0	0	0	0	0	1	0	2	0
k__Bacteria	p__Cyanobacteria	c__4C0d-2	o__YS2	Unclassified	Unclassified	Unclassified	0	3	95	0	0	0	0	0	0	0	0	17	0	0	0	0	0	0	0
k__Bacteria	p__Bacteroidetes	c__Bacteroidia	o__Bacteroidales	f__S24-7	Unclassified	Unclassified	0	1	0	1	0	0	1	0	2	0	1	1	3	0	0	0	0	4	3
k__Bacteria	p__Firmicutes	c__Clostridia	o__Clostridiales	f__Lachnospiraceae	g__Dorea	Unclassified	0	1	14	18	0	0	1	2	5	4	5	3	0	3	3	2	6	3	0
k__Bacteria	p__Tenericutes	c__Mollicutes	o__RF39	Unclassified	Unclassified	Unclassified	0	2	0	0	0	0	0	0	0	0	0	0	0	0	0	0	11	47	0
k__Bacteria	p__Deferribacteres	c__Deferribacteres	o__Deferribacterales	f__Deferribacteraceae	g__Mucispirillum	s__schaedleri	0	2	15	14	0	3	4	0	0	1	1	30	6	10	4	0	0	1	1
k__Bacteria	p__Firmicutes	c__Erysipelotrichi	o__Erysipelotrichales	f__Erysipelotrichaceae	g__RFN20	Unclassified	0	2	7	3	4	4	4	6	10	2	0	0	10	13	2	3	4	8	9
k__Bacteria	p__Firmicutes	c__Clostridia	o__Clostridiales	Unclassified	Unclassified	Unclassified	0	2	0	2	2	0	6	5	0	0	0	5	0	17	1	0	1	0	1
k__Bacteria	p__Bacteroidetes	c__Bacteroidia	o__Bacteroidales	Unclassified	Unclassified	Unclassified	0	1	0	0	0	1	0	2	0	6	1	0	1	0	0	2	0	0	1
k__Bacteria	p__Firmicutes	c__Clostridia	o__Clostridiales	f__Ruminococcaceae	g__Ruminococcus	Unclassified	0	1	4	4	0	0	5	8	2	3	1	2	5	0	0	0	1	7	5
k__Bacteria	p__Bacteroidetes	c__Bacteroidia	o__Bacteroidales	Unclassified	Unclassified	Unclassified	0	3	0	2	0	0	0	0	0	0	0	0	0	0	0	0	3	4	0
k__Bacteria	p__Firmicutes	c__Clostridia	o__Clostridiales	f__Lachnospiraceae	Unclassified	Unclassified	0	1	0	16	2	12	53	23	0	1	0	193	5	0	18	0	20	0	11

Copyright © 2020, Sociedade Brasileira de Genética

Level_1	Level_2	Level_3	Level_4	Level_5	Level_6	Level_7	ss10	sn15	sn16	ss19	sn20	ss21	ss25	sn26	sn27	ss28	ss2	ss30	sn31	sn35	sn36	sn5	ss6	ss8	sn9
k__Bacteria	p__Bacteroidetes	c__Bacteroidia	o__Bacteroidales	f__[Paraprevotellaceae]	g__Paraprevotella	Unclassified	0	3	4	1	23	14	19	29	27	0	6	3	0	6	3	27	70	91	15
k__Bacteria	p__Firmicutes	c__Clostridia	o__Clostridiales	Unclassified	Unclassified	Unclassified	0	3	0	145	0	22	0	0	0	0	0	0	5	2	1	44	85	112	399
k__Bacteria	p__Bacteroidetes	c__Bacteroidia	o__Bacteroidales	Unclassified	Unclassified	Unclassified	0	3	94	67	40	18	33	2	30	21	9	5	10	30	40	20	7	57	33
k__Bacteria	p__Firmicutes	c__Clostridia	o__Clostridiales	f__Ruminococcaceae	g__Oscillospira	Unclassified	0	1	1	0	0	0	0	0	0	0	0	0	2	0	2	0	2	0	0
k__Bacteria	Unclassified	Unclassified	Unclassified	Unclassified	Unclassified	Unclassified	0	1	0	3	0	0	12	2	0	9	0	0	0	47	9	0	0	1	0
k__Bacteria	Unclassified	Unclassified	Unclassified	Unclassified	Unclassified	Unclassified	0	1	0	2	0	0	14	3	0	0	0	0	0	0	0	0	8	2	0
k__Bacteria	Unclassified	Unclassified	Unclassified	Unclassified	Unclassified	Unclassified	0	1	0	0	0	0	0	0	0	0	0	0	0	0	0	0	0	3	0
k__Bacteria	p__Firmicutes	c__Clostridia	o__Clostridiales	Unclassified	Unclassified	Unclassified	0	1	0	0	3	1	0	7	3	0	1	19	0	1	3	0	1	0	2
k__Bacteria	Unclassified	Unclassified	Unclassified	Unclassified	Unclassified	Unclassified	0	1	0	0	0	2	0	0	6	0	0	59	24	0	32	0	0	0	2
k__Bacteria	p__Tenericutes	c__Mollicutes	o__RF39	Unclassified	Unclassified	Unclassified	0	1	0	1	0	0	0	0	0	0	3	0	1	8	5	0	8	6	3
k__Bacteria	p__Firmicutes	c__Clostridia	o__Clostridiales	Unclassified	Unclassified	Unclassified	0	3	0	0	0	0	0	0	0	0	0	0	0	0	0	0	0	0	0
k__Bacteria	Unclassified	Unclassified	Unclassified	Unclassified	Unclassified	Unclassified	0	1	0	0	0	0	1	0	0	0	0	0	0	0	0	0	3	0	0
k__Bacteria	p__Bacteroidetes	c__Bacteroidia	o__Bacteroidales	f__[Paraprevotellaceae]	Unclassified	Unclassified	0	1	0	0	0	0	4	0	0	0	2	0	0	0	0	0	0	6	0
k__Bacteria	p__Firmicutes	c__Clostridia	o__Clostridiales	f__Veillonellaceae	Unclassified	Unclassified	0	1	24	0	0	10	1	0	4	1	0	14	0	54	5	1	0	0	0

Copyright © 2020, Sociedade Brasileira de Genética

Level_1	Level_2	Level_3	Level_4	Level_5	Level_6	Level_7	ss10	sn15	sn16	ss19	sn20	ss21	ss25	sn26	sn27	ss28	ss2	ss30	sn31	sn35	sn36	sn5	ss6	ss8	sn9
k__Bacteria	p__Firmicutes	c__Clostridia	o__Clostridiales	f__Ruminococcaceae	Unclassified	Unclassified	0	2	25	11	0	4	2	0	2	5	7	1	3	12	4	1	5	0	1
k__Bacteria	p__Firmicutes	c__Clostridia	o__Clostridiales	Unclassified	Unclassified	Unclassified	0	1	39	5	21	14	27	29	14	0	13	44	27	31	21	4	30	75	25
k__Bacteria	p__Firmicutes	c__Clostridia	o__Clostridiales	Unclassified	Unclassified	Unclassified	0	1	8	0	0	0	0	0	0	0	0	0	0	3	0	0	1	0	0
k__Bacteria	p__Tenericutes	c__Mollicutes	o__RF39	Unclassified	Unclassified	Unclassified	0	1	0	2	0	0	0	0	45	0	81	0	4	0	29	5	0	2	7
k__Bacteria	p__Cyanobacteria	c__4C0d-2	o__YS2	Unclassified	Unclassified	Unclassified	0	2	63	0	0	0	0	3	22	96	5	0	0	6	61	16	0	0	2
k__Bacteria	p__Firmicutes	c__Clostridia	o__Clostridiales	f__[Mogibacteriaceae]	Unclassified	Unclassified	0	1	0	5	0	1	1	2	4	3	1	6	1	3	0	1	2	2	0
k__Bacteria	p__Firmicutes	c__Clostridia	o__Clostridiales	Unclassified	Unclassified	Unclassified	0	1	3	0	2	0	19	2	0	4	1	3	1	6	4	0	45	100	6
k__Bacteria	p__Firmicutes	c__Clostridia	o__Clostridiales	f__Lachnospiraceae	Unclassified	Unclassified	0	1	0	19	9	0	25	0	12	0	115	25	1	152	117	0	2	2	5
k__Bacteria	p__Firmicutes	c__Clostridia	o__Clostridiales	f__Ruminococcaceae	Unclassified	Unclassified	0	1	1	0	0	2	3	2	1	1	1	0	0	34	0	1	1	0	2
k__Bacteria	p__Actinobacteria	c__Coriobacteriia	o__Coriobacteriales	f__Coriobacteriaceae	Unclassified	Unclassified	0	1	0	0	0	0	0	0	0	1	1	0	0	0	0	0	1	0	0
k__Bacteria	p__Firmicutes	c__Clostridia	o__Clostridiales	Unclassified	Unclassified	Unclassified	0	1	0	6	0	0	0	0	0	49	0	1	0	0	0	0	0	0	0
k__Bacteria	p__Firmicutes	c__Clostridia	o__Clostridiales	Unclassified	Unclassified	Unclassified	0	1	0	0	0	1	0	1	0	0	0	0	0	1	0	0	0	0	0
k__Bacteria	p__Firmicutes	c__Clostridia	o__Clostridiales	f__Ruminococcaceae	Unclassified	Unclassified	0	1	0	1	1	1	0	2	3	0	1	0	0	0	0	0	0	0	0
k__Bacteria	p__Firmicutes	c__Clostridia	o__Clostridiales	f__Ruminococcaceae	Unclassified	Unclassified	0	1	0	4	0	0	0	0	1	0	0	4	0	0	0	0	0	4	0

Copyright © 2020, Sociedade Brasileira de Genética

**Level_1**	**Level_2**	**Level_3**	**Level_4**	**Level_5**	**Level_6**	**Level_7**	**ss10**	**sn15**	**sn16**	**ss19**	**sn20**	**ss21**	**ss25**	**sn26**	**sn27**	**ss28**	**ss2**	**ss30**	**sn31**	**sn35**	**sn36**	**sn5**	**ss6**	**ss8**	**sn9**
k__Bacteria	p__Firmicutes	c__Clostridia	o__Clostridiales	f__Lachnospiraceae	Unclassified	Unclassified	0	1	0	0	0	0	1	1	2	0	0	1	0	1	2	1	0	0	1
k__Bacteria	p__Firmicutes	c__Clostridia	o__Clostridiales	Unclassified	Unclassified	Unclassified	0	2	2	8	0	5	9	3	4	36	6	5	7	1	51	0	2	2	6
k__Bacteria	p__Bacteroidetes	c__Bacteroidia	o__Bacteroidales	f__Prevotellaceae	g__Prevotella	Unclassified	0	1	0	0	1	0	4	3	3	0	0	1	2	2	2	1	3	3	5
k__Bacteria	p__Firmicutes	c__Clostridia	o__Clostridiales	Unclassified	Unclassified	Unclassified	0	1	0	4	0	0	2	0	0	0	0	0	0	0	0	0	0	0	0
k__Bacteria	p__Proteobacteria	c__Deltaproteobacteria	o__Desulfovibrionales	f__Desulfovibrionaceae	Unclassified	Unclassified	0	1	3	0	0	0	0	0	0	0	0	1	0	2	0	0	0	0	0
k__Bacteria	p__Firmicutes	c__Clostridia	o__Clostridiales	Unclassified	Unclassified	Unclassified	0	2	4	0	1	0	0	2	0	7	0	0	0	4	0	0	0	1	0
k__Bacteria	p__Tenericutes	c__Mollicutes	o__RF39	Unclassified	Unclassified	Unclassified	0	1	0	1	0	5	0	0	0	0	0	0	2	0	5	0	0	6	0
k__Bacteria	p__Firmicutes	c__Clostridia	o__Clostridiales	Unclassified	Unclassified	Unclassified	0	1	45	0	0	0	0	0	0	0	0	0	0	0	0	0	0	0	0
k__Bacteria	p__Firmicutes	c__Clostridia	o__Clostridiales	Unclassified	Unclassified	Unclassified	0	1	0	0	0	0	0	0	3	0	0	2	0	4	3	0	0	0	0
k__Bacteria	p__Firmicutes	c__Clostridia	o__Clostridiales	Unclassified	Unclassified	Unclassified	0	1	0	0	0	0	0	6	0	0	0	0	2	0	0	0	0	0	0
k__Bacteria	p__Firmicutes	c__Clostridia	o__Clostridiales	Unclassified	Unclassified	Unclassified	0	1	0	8	0	2	3	0	0	0	1	26	9	3	4	0	2	1	1
k__Bacteria	p__Firmicutes	c__Clostridia	o__Clostridiales	f__Lachnospiraceae	Unclassified	Unclassified	0	1	0	0	0	1	0	0	0	0	0	0	0	1	0	0	0	0	6
k__Bacteria	p__Firmicutes	c__Clostridia	o__Clostridiales	Unclassified	Unclassified	Unclassified	0	1	7	0	1	3	2	5	3	0	1	0	6	14	2	0	1	44	9
k__Bacteria	p__Bacteroidetes	c__Bacteroidia	o__Bacteroidales	f__[Odoribacteraceae]	g__Odoribacter	Unclassified	0	2	4	11	1	0	1	5	2	1	5	11	0	25	0	0	0	0	0

Copyright © 2020, Sociedade Brasileira de Genética

Level_1	Level_2	Level_3	Level_4	Level_5	Level_6	Level_7	ss10	sn15	sn16	ss19	sn20	ss21	ss25	sn26	sn27	ss28	ss2	ss30	sn31	sn35	sn36	sn5	ss6	ss8	sn9
k__Bacteria	p__Firmicutes	c__Clostridia	o__Clostridiales	f__Ruminococcaceae	g__Oscillospira	Unclassified	0	1	1	0	1	0	0	0	2	0	0	0	0	7	2	0	0	1	0
k__Bacteria	p__Firmicutes	c__Clostridia	o__Clostridiales	Unclassified	Unclassified	Unclassified	0	1	0	0	1	0	0	0	0	0	1	0	2	0	0	2	1	4	0
k__Bacteria	p__Proteobacteria	c__Deltaproteobacteria	o__Desulfovibrionales	f__Desulfovibrionaceae	Unclassified	Unclassified	0	1	12	6	6	0	0	0	0	0	0	0	0	2	0	0	8	2	0
k__Bacteria	p__Firmicutes	c__Clostridia	o__Clostridiales	f__Lachnospiraceae	Unclassified	Unclassified	0	0	1163	12	0	0	0	0	0	0	1	1	0	3	30	1	0	0	399
k__Bacteria	p__Firmicutes	c__Clostridia	o__Clostridiales	Unclassified	Unclassified	Unclassified	0	0	34	10	0	0	0	0	0	0	1	0	0	0	0	1	0	36	9
k__Bacteria	p__Firmicutes	c__Clostridia	o__Clostridiales	Unclassified	Unclassified	Unclassified	0	0	10	5	3	0	14	22	9	0	2	10	0	4	6	4	0	4	9
k__Bacteria	p__Firmicutes	c__Clostridia	o__Clostridiales	f__Ruminococcaceae	Unclassified	Unclassified	0	0	53	7	0	0	10	5	0	0	12	2	0	24	1	4	45	7	3
k__Bacteria	p__Firmicutes	c__Clostridia	o__Clostridiales	Unclassified	Unclassified	Unclassified	0	0	4	1	2	0	0	1	1	1	1	12	1	6	0	1	0	1	1
k__Bacteria	p__Bacteroidetes	c__Bacteroidia	o__Bacteroidales	f__S24-7	Unclassified	Unclassified	0	0	29	0	0	0	9	0	0	3	32	0	0	0	0	0	0	100	99
k__Bacteria	p__Cyanobacteria	c__4C0d-2	o__YS2	Unclassified	Unclassified	Unclassified	0	0	20	0	0	0	0	1	0	0	1	0	0	134	29	0	0	0	0
k__Bacteria	Unclassified	Unclassified	Unclassified	Unclassified	Unclassified	Unclassified	0	0	12	1	1	0	5	6	1	0	0	6	1	0	0	0	0	0	4
k__Bacteria	p__Firmicutes	c__Clostridia	o__Clostridiales	f__Ruminococcaceae	Unclassified	Unclassified	0	0	4	1	0	0	0	0	0	0	0	4	1	1	1	0	1	0	2
k__Bacteria	p__Bacteroidetes	c__Bacteroidia	o__Bacteroidales	f__Prevotellaceae	g__Prevotella	Unclassified	0	0	22	0	0	0	0	0	23	53	0	0	0	0	106	0	0	0	0
k__Bacteria	p__Proteobacteria	c__Deltaproteobacteria	o__Desulfovibrionales	f__Desulfovibrionaceae	Unclassified	Unclassified	0	0	4	12	1	1	0	0	0	0	0	27	1	5	1	1	1	0	2

Copyright © 2020, Sociedade Brasileira de Genética

**Level_1**	**Level_2**	**Level_3**	**Level_4**	**Level_5**	**Level_6**	**Level_7**	**ss10**	**sn15**	**sn16**	**ss19**	**sn20**	**ss21**	**ss25**	**sn26**	**sn27**	**ss28**	**ss2**	**ss30**	**sn31**	**sn35**	**sn36**	**sn5**	**ss6**	**ss8**	**sn9**
k__Bacteria	p__Verrucomicrobia	c__Verruco-5	o__WCHB1-41	f__RFP12	Unclassified	Unclassified	0	0	15	0	0	1	0	0	1	0	0	0	1	0	0	0	0	1	0
k__Bacteria	p__Proteobacteria	c__Alphaproteobacteria	o__RF32	Unclassified	Unclassified	Unclassified	0	0	14	0	0	0	0	0	0	0	0	5	0	0	0	0	0	0	0
k__Bacteria	p__Tenericutes	c__Mollicutes	o__RF39	Unclassified	Unclassified	Unclassified	0	0	6	0	0	0	0	0	0	0	0	0	0	0	0	0	0	28	0
k__Bacteria	p__Bacteroidetes	c__Bacteroidia	o__Bacteroidales	f__S24-7	Unclassified	Unclassified	0	0	10	1	0	3	0	14	1	0	0	1	0	0	5	0	0	4	6
k__Bacteria	p__Bacteroidetes	c__Bacteroidia	o__Bacteroidales	f__Prevotellaceae	g__Prevotella	Unclassified	0	0	2	2	1	0	0	0	0	1	1	1	0	0	0	0	0	0	3
k__Bacteria	p__Proteobacteria	c__Gammaproteobacteria	o__Aeromonadales	f__Succinivibrionaceae	g__Anaerobiospirillum	Unclassified	0	0	14	0	0	9	2	0	2	0	0	6	5	1	2	4	0	2	0
k__Bacteria	p__Firmicutes	c__Clostridia	o__Clostridiales	Unclassified	Unclassified	Unclassified	0	0	5	4	0	0	0	0	0	1	0	7	1	5	0	0	1	2	3
k__Bacteria	p__Firmicutes	c__Clostridia	o__Clostridiales	f__Ruminococcaceae	g__Faecalibacterium	s__prausnitzii	0	0	2	0	0	0	2	0	0	3	1	0	0	1	0	17	0	0	0
k__Bacteria	p__Bacteroidetes	c__Bacteroidia	o__Bacteroidales	f__S24-7	Unclassified	Unclassified	0	0	20	47	22	1	30	98	0	14	0	0	2	2	14	26	0	0	24
k__Bacteria	p__Firmicutes	c__Clostridia	o__Clostridiales	f__Lachnospiraceae	Unclassified	Unclassified	0	0	3	1	0	0	0	0	0	0	0	2	0	0	1	0	0	0	0
k__Bacteria	p__Firmicutes	c__Clostridia	o__Clostridiales	f__Ruminococcaceae	Unclassified	Unclassified	0	0	3	4	0	0	1	3	0	0	0	1	1	0	0	0	0	2	0
k__Bacteria	p__Bacteroidetes	c__Bacteroidia	o__Bacteroidales	f__S24-7	Unclassified	Unclassified	0	0	40	0	0	0	4	1528	0	3	27	0	0	0	23	14	0	184	20
k__Bacteria	p__Bacteroidetes	c__Bacteroidia	o__Bacteroidales	f__Prevotellaceae	g__Prevotella	Unclassified	0	0	8	0	0	0	0	0	0	0	0	4	6	0	0	2	0	0	0
k__Bacteria	Unclassified	Unclassified	Unclassified	Unclassified	Unclassified	Unclassified	0	0	7	19	0	1	0	17	1	0	0	4	0	13	0	0	0	0	1

Copyright © 2020, Sociedade Brasileira de Genética

Level_1	Level_2	Level_3	Level_4	Level_5	Level_6	Level_7	ss10	sn15	sn16	ss19	sn20	ss21	ss25	sn26	sn27	ss28	ss2	ss30	sn31	sn35	sn36	sn5	ss6	ss8	sn9
k__Bacteria	p__Bacteroidetes	c__Bacteroidia	o__Bacteroidales	Unclassified	Unclassified	Unclassified	0	0	2	0	0	0	0	0	0	0	0	0	0	0	0	0	0	0	0
k__Bacteria	p__Firmicutes	c__Clostridia	o__Clostridiales	Unclassified	Unclassified	Unclassified	0	0	3	0	0	0	0	0	0	0	0	31	0	1	0	0	0	0	0
k__Bacteria	p__Firmicutes	c__Erysipelotrichi	o__Erysipelotrichales	f__Erysipelotrichaceae	g__p-75-a5	Unclassified	0	0	8	0	2	0	1	11	10	0	2	2	1	5	1	0	7	0	1
k__Bacteria	p__Bacteroidetes	c__Bacteroidia	o__Bacteroidales	f__S24-7	Unclassified	Unclassified	0	0	4	5	0	3	3	0	20	1	0	8	2	0	0	0	3	0	1
k__Bacteria	p__Firmicutes	c__Clostridia	o__Clostridiales	f__Lachnospiraceae	Unclassified	Unclassified	0	0	8	1	1	0	1	4	1	0	0	3	0	0	1	1	0	0	0
k__Bacteria	p__Firmicutes	c__Clostridia	o__Clostridiales	f__Ruminococcaceae	g__Oscillospira	Unclassified	0	0	3	10	2	2	16	8	5	1	0	7	4	1	3	0	10	2	6
k__Bacteria	p__Firmicutes	c__Clostridia	o__Clostridiales	Unclassified	Unclassified	Unclassified	0	0	2	1	0	1	0	1	0	1	0	7	1	1	0	1	0	0	0
k__Bacteria	p__Firmicutes	c__Clostridia	o__Clostridiales	f__Ruminococcaceae	Unclassified	Unclassified	0	0	12	5	26	12	0	34	0	3	14	0	0	102	6	0	1	0	0
k__Bacteria	p__Firmicutes	c__Clostridia	o__Clostridiales	Unclassified	Unclassified	Unclassified	0	0	7	0	0	0	1	2	0	2	0	0	0	1	0	0	0	0	0
k__Bacteria	p__Firmicutes	c__Clostridia	o__Clostridiales	Unclassified	Unclassified	Unclassified	0	0	5	26	3	0	3	1	0	0	21	237	2	36	49	0	0	80	134
k__Bacteria	p__Firmicutes	c__Clostridia	o__Clostridiales	f__Ruminococcaceae	Unclassified	Unclassified	0	0	2	4	1	2	0	0	3	0	1	0	0	0	1	2	1	2	0
k__Bacteria	p__Proteobacteria	c__Betaproteobacteria	o__Burkholderiales	f__Alcaligenaceae	g__Sutterella	Unclassified	0	0	5	0	0	0	6	2	0	1	0	0	0	14	6	0	0	0	0
k__Bacteria	Unclassified	Unclassified	Unclassified	Unclassified	Unclassified	Unclassified	0	0	6	0	0	6	0	6	0	2	3	4	0	0	22	0	0	0	0
k__Bacteria	p__Firmicutes	c__Clostridia	o__Clostridiales	f__Lachnospiraceae	Unclassified	Unclassified	0	0	2	0	5	1	2	2	1	0	0	1	0	0	0	2	3	0	0

Genetics and Molecular Biology 43, 1 (2020)
Copyright © 2020, Sociedade Brasileira de Genética

**Level_1**	**Level_2**	**Level_3**	**Level_4**	**Level_5**	**Level_6**	**Level_7**	**ss10**	**sn15**	**sn16**	**ss19**	**sn20**	**ss21**	**ss25**	**sn26**	**sn27**	**ss28**	**ss2**	**ss30**	**sn31**	**sn35**	**sn36**	**sn5**	**ss6**	**ss8**	**sn9**
k__Bacteria	p__Proteobacteria	c__Betaproteobacteria	o__Burkholderiales	f__Comamonadaceae	Unclassified	Unclassified	0	0	2	0	1	0	0	0	0	0	0	2	0	0	1	1	0	1	0
k__Bacteria	p__Firmicutes	c__Clostridia	o__Clostridiales	f__Ruminococcaceae	g__Ruminococcus	s__callidus	0	0	6	0	0	0	0	0	17	0	1	0	0	0	16	0	36	0	0
k__Bacteria	p__Actinobacteria	c__Coriobacteriia	o__Coriobacteriales	f__Coriobacteriaceae	g__Collinsella	Unclassified	0	0	1	1	0	1	0	0	2	1	0	0	0	0	0	0	0	1	0
k__Bacteria	p__Firmicutes	c__Clostridia	o__Clostridiales	f__Lachnospiraceae	Unclassified	Unclassified	0	0	2	7	0	2	0	0	1	0	0	1	0	3	1	0	0	0	0
k__Bacteria	p__Firmicutes	c__Clostridia	o__Clostridiales	f__Ruminococcaceae	g__Ruminococcus	Unclassified	0	0	1	0	0	0	2	0	0	1	0	0	0	0	0	0	0	0	0
k__Bacteria	p__Firmicutes	c__Clostridia	o__Clostridiales	f__Lachnospiraceae	g__[Ruminococcus]	s__gnavus	0	0	10	0	1	0	0	1	1	0	1	14	2	3	0	0	0	0	0
k__Bacteria	Unclassified	Unclassified	Unclassified	Unclassified	Unclassified	Unclassified	0	0	5	0	0	0	0	0	7	0	7	1	5	0	0	0	0	3	0
k__Bacteria	p__Tenericutes	c__Mollicutes	o__RF39	Unclassified	Unclassified	Unclassified	0	0	1	0	0	0	0	0	0	0	3	0	0	1	0	0	0	0	1
k__Bacteria	p__Bacteroidetes	c__Bacteroidia	o__Bacteroidales	Unclassified	Unclassified	Unclassified	0	0	4	0	0	0	1	1	0	5	0	0	0	0	4	0	0	0	0
k__Bacteria	p__Firmicutes	c__Clostridia	o__Clostridiales	f__Ruminococcaceae	Unclassified	Unclassified	0	0	4	5	0	0	0	0	0	0	1	5	1	2	0	1	2	2	0
k__Bacteria	p__Firmicutes	c__Clostridia	o__Clostridiales	Unclassified	Unclassified	Unclassified	0	0	4	0	0	0	0	0	0	0	0	0	0	2	0	0	0	23	1
k__Bacteria	p__Bacteroidetes	c__Bacteroidia	o__Bacteroidales	f__S24-7	Unclassified	Unclassified	0	0	3	0	0	12	21	53	2	8	0	3	1	0	2	27	0	0	33
k__Bacteria	p__Firmicutes	c__Erysipelotrichi	o__Erysipelotrichales	f__Erysipelotrichaceae	g__Allobaculum	Unclassified	0	0	5	15	5	8	1	6	4	1	0	0	1	0	9	0	0	0	12
k__Bacteria	p__Firmicutes	c__Clostridia	o__Clostridiales	f__Lachnospiraceae	Unclassified	Unclassified	0	0	1	2	0	0	0	0	0	1	1	5	0	10	0	0	0	0	0

Copyright © 2020, Sociedade Brasileira de Genética

Level_1	Level_2	Level_3	Level_4	Level_5	Level_6	Level_7	ss10	sn15	sn16	ss19	sn20	ss21	ss25	sn26	sn27	ss28	ss2	ss30	sn31	sn35	sn36	sn5	ss6	ss8	sn9
k__Bacteria	Unclassified	Unclassified	Unclassified	Unclassified	Unclassified	Unclassified	0	0	8	0	3	0	2	0	1	0	0	0	0	12	1	0	0	3	0
k__Bacteria	p__Proteobacteria	c__Deltaproteobacteria	o__Desulfovibrionales	f__Desulfovibrionaceae	Unclassified	Unclassified	0	0	1	0	1	1	0	0	0	0	0	1	0	2	2	0	0	3	0
k__Bacteria	p__Firmicutes	c__Clostridia	o__Clostridiales	f__Lachnospiraceae	Unclassified	Unclassified	0	0	2	1	3	1	2	2	1	0	0	3	0	0	0	1	1	0	0
k__Bacteria	p__Firmicutes	Unclassified	Unclassified	Unclassified	Unclassified	Unclassified	0	0	4	0	0	0	0	13	0	5	0	20	2	0	0	3	1	0	0
k__Bacteria	p__Firmicutes	c__Clostridia	o__Clostridiales	f__Lachnospiraceae	Unclassified	Unclassified	0	0	7	3	0	0	0	1	0	0	0	1	0	0	0	0	0	0	0
k__Bacteria	p__Firmicutes	c__Clostridia	o__Clostridiales	f__Lachnospiraceae	Unclassified	Unclassified	0	0	2	0	0	0	0	8	0	0	0	0	0	1	0	0	1	0	0
k__Bacteria	p__Bacteroidetes	c__Bacteroidia	o__Bacteroidales	f__Rikenellaceae	Unclassified	Unclassified	0	0	3	0	1	0	1	6	3	0	0	1	0	15	2	0	0	2	0
k__Bacteria	p__Bacteroidetes	c__Bacteroidia	o__Bacteroidales	f__S24-7	Unclassified	Unclassified	0	0	2	2	0	5	1	5	3	2	2	1	2	0	2	1	3	5	2
k__Bacteria	Unclassified	Unclassified	Unclassified	Unclassified	Unclassified	Unclassified	0	0	1	0	0	0	0	0	0	0	0	1	1	1	0	0	0	0	0
k__Bacteria	p__Firmicutes	c__Clostridia	o__Clostridiales	f__Ruminococcaceae	Unclassified	Unclassified	0	0	3	3	0	1	0	0	0	0	0	0	0	0	0	0	0	0	0
k__Bacteria	p__Firmicutes	c__Clostridia	o__Clostridiales	f__Ruminococcaceae	Unclassified	Unclassified	0	0	2	3	0	1	1	0	1	0	0	3	0	1	1	0	1	2	1
k__Bacteria	p__Bacteroidetes	c__Bacteroidia	o__Bacteroidales	f__S24-7	Unclassified	Unclassified	0	0	4	0	5	0	7	38	7	2	0	0	0	0	1	0	0	0	0
k__Bacteria	p__Firmicutes	c__Erysipelotrichi	o__Erysipelotrichales	f__Erysipelotrichaceae	g__RFN20	Unclassified	0	0	2	2	0	2	0	0	3	2	0	2	0	0	0	0	0	0	1
k__Bacteria	Unclassified	Unclassified	Unclassified	Unclassified	Unclassified	Unclassified	0	0	1	0	3	0	0	0	5	0	0	0	0	0	1	0	0	3	2

Copyright © 2020, Sociedade Brasileira de Genética

Level_1	Level_2	Level_3	Level_4	Level_5	Level_6	Level_7	ss10	sn15	sn16	ss19	sn20	ss21	ss25	sn26	sn27	ss28	ss2	ss30	sn31	sn35	sn36	sn5	ss6	ss8	sn9
k__Bacteria	p__Firmicutes	c__Clostridia	o__Clostridiales	f__Lachnospiraceae	Unclassified	Unclassified	0	0	2	3	0	0	1	3	0	0	1	17	8	1	6	0	0	0	1
k__Bacteria	p__Firmicutes	c__Clostridia	o__Clostridiales	f__Ruminococcaceae	Unclassified	Unclassified	0	0	3	0	0	1	0	0	0	0	0	0	11	3	0	0	0	25	0
k__Bacteria	p__Firmicutes	c__Clostridia	o__Clostridiales	f__Ruminococcaceae	Unclassified	Unclassified	0	0	1	0	1	0	0	0	0	0	1	0	0	0	3	0	0	0	0
k__Bacteria	p__Firmicutes	c__Clostridia	o__Clostridiales	f__Lachnospiraceae	Unclassified	Unclassified	0	0	2	4	1	1	2	1	0	0	1	4	1	3	2	1	0	0	0
k__Bacteria	p__Bacteroidetes	c__Bacteroidia	o__Bacteroidales	f__S24-7	Unclassified	Unclassified	0	0	2	0	1	9	1	6	1	1	0	1	0	0	2	0	0	0	0
k__Bacteria	p__Firmicutes	c__Clostridia	o__Clostridiales	Unclassified	Unclassified	Unclassified	0	0	1	3	0	0	0	1	0	0	0	3	0	0	0	0	0	1	0
k__Bacteria	p__Firmicutes	c__Clostridia	o__Clostridiales	f__Lachnospiraceae	Unclassified	Unclassified	0	0	1	242	19	9	0	0	0	0	0	0	0	3	1	0	0	1	0
k__Bacteria	p__Firmicutes	c__Clostridia	o__Clostridiales	f__Ruminococcaceae	Unclassified	Unclassified	0	0	2	9	0	2	1	0	10	3	2	9	3	9	8	3	2	5	1
k__Bacteria	p__Bacteroidetes	c__Bacteroidia	o__Bacteroidales	f__Prevotellaceae	g__Prevotella	Unclassified	0	0	2	0	0	0	0	0	0	0	0	0	0	0	0	0	0	1	0
k__Bacteria	p__Firmicutes	c__Clostridia	o__Clostridiales	f__Lachnospiraceae	Unclassified	Unclassified	0	0	1	0	0	0	0	0	0	0	0	0	0	10	0	0	0	0	0
k__Bacteria	p__Spirochaetes	c__Spirochaetes	o__Spirochaetales	f__Spirochaetaceae	Unclassified	Unclassified	0	0	2	18	0	3	3	0	2	2	2	76	0	0	0	0	0	0	0
k__Bacteria	p__Firmicutes	c__Clostridia	o__Clostridiales	f__Ruminococcaceae	g__Ruminococcus	Unclassified	0	0	1	0	0	0	0	0	0	2	0	0	0	0	0	0	0	0	0
k__Bacteria	p__Firmicutes	c__Erysipelotrichi	o__Erysipelotrichales	f__Erysipelotrichaceae	g__Allobaculum	Unclassified	0	0	3	8	0	2	0	0	5	0	0	0	1	0	1	0	0	1	7
k__Bacteria	p__Firmicutes	c__Clostridia	o__Clostridiales	f__Ruminococcaceae	g__Ruminococcus	Unclassified	0	0	1	3	31	0	0	15	0	0	0	0	0	1	0	0	0	0	26

Copyright © 2020, Sociedade Brasileira de Genética

Level_1	Level_2	Level_3	Level_4	Level_5	Level_6	Level_7	ss10	sn15	sn16	ss19	sn20	ss21	ss25	sn26	sn27	ss28	ss2	ss30	sn31	sn35	sn36	sn5	ss6	ss8	sn9
k__Bacteria	p__Firmicutes	c__Clostridia	o__Clostridiales	Unclassified	Unclassified	Unclassified	0	0	2	8	0	0	0	0	0	0	0	0	0	0	1	0	0	22	10
k__Bacteria	p__Firmicutes	c__Clostridia	o__Clostridiales	f__Ruminococcaceae	g__Oscillospira	Unclassified	0	0	1	2	0	1	0	3	2	2	3	8	1	6	3	1	2	0	1
k__Bacteria	p__Firmicutes	c__Clostridia	o__Clostridiales	f__Lachnospiraceae	Unclassified	Unclassified	0	0	2	1	0	0	0	0	0	0	0	1	0	0	0	0	0	0	0
k__Bacteria	p__Firmicutes	c__Clostridia	o__Clostridiales	Unclassified	Unclassified	Unclassified	0	0	1	0	0	0	0	0	0	0	0	1	0	3	0	0	1	7	0
k__Bacteria	p__Firmicutes	c__Erysipelotrichi	o__Erysipelotrichales	f__Erysipelotrichaceae	g__Coprobacillus	Unclassified	0	0	1	0	5	0	4	0	0	3	0	0	0	0	1	0	0	0	2
k__Bacteria	p__Firmicutes	c__Clostridia	o__Clostridiales	f__Ruminococcaceae	Unclassified	Unclassified	0	0	1	8	0	0	0	0	0	0	1	2	0	0	0	0	0	0	0
k__Bacteria	p__Firmicutes	c__Clostridia	o__Clostridiales	f__Lachnospiraceae	g__Coprococcus	Unclassified	0	0	1	2	0	0	0	0	0	0	0	0	0	0	0	0	0	22	29
k__Bacteria	p__Firmicutes	c__Clostridia	o__Clostridiales	f__Lachnospiraceae	Unclassified	Unclassified	0	0	1	0	0	1	2	0	0	0	0	1	0	2	0	0	0	0	0
k__Bacteria	p__Firmicutes	c__Clostridia	o__Clostridiales	f__Ruminococcaceae	Unclassified	Unclassified	0	0	1	0	0	1	0	0	0	0	0	0	0	3	0	0	0	0	0
k__Bacteria	Unclassified	Unclassified	Unclassified	Unclassified	Unclassified	Unclassified	0	0	1	0	0	0	5	0	0	0	0	0	0	0	0	0	0	0	0
k__Bacteria	p__Firmicutes	c__Clostridia	o__Clostridiales	f__Ruminococcaceae	g__Oscillospira	Unclassified	0	0	1	1	1	0	2	0	1	0	0	2	0	6	0	0	0	0	0
k__Bacteria	p__Firmicutes	c__Clostridia	o__Clostridiales	f__Ruminococcaceae	g__Oscillospira	Unclassified	0	0	1	0	2	0	4	9	0	0	0	5	2	0	0	0	1	5	4
k__Bacteria	Unclassified	Unclassified	Unclassified	Unclassified	Unclassified	Unclassified	0	0	1	0	0	0	0	0	0	3	0	10	3	4	0	0	1	0	0
k__Bacteria	p__Firmicutes	c__Clostridia	o__Clostridiales	f__Ruminococcaceae	g__Ruminococcus	Unclassified	0	0	1	1	1	0	0	0	0	0	0	0	1	0	0	1	1	0	4

Copyright © 2020, Sociedade Brasileira de Genética

Level_1	Level_2	Level_3	Level_4	Level_5	Level_6	Level_7	ss10	sn15	sn16	ss19	sn20	ss21	ss25	sn26	sn27	ss28	ss2	ss30	sn31	sn35	sn36	sn5	ss6	ss8	sn9
k__Bacteria	p__Bacteroidetes	c__Bacteroidia	o__Bacteroidales	f__[Paraprevotellaceae]	g__[Prevotella]	Unclassified	0	0	0	1677	16	55	0	133	0	0	0	76	11	0	72	3	25	47	0
k__Bacteria	p__Firmicutes	c__Clostridia	o__Clostridiales	f__[Mogibacteriaceae]	Unclassified	Unclassified	0	0	0	26	11	9	22	12	31	0	13	45	14	0	22	4	51	10	24
k__Bacteria	p__Firmicutes	c__Clostridia	o__Clostridiales	Unclassified	Unclassified	Unclassified	0	0	0	53	0	50	0	0	0	0	0	627	143	0	0	0	0	153	156
k__Bacteria	p__Bacteroidetes	c__Bacteroidia	o__Bacteroidales	f__Bacteroidaceae	g__Bacteroides	Unclassified	0	0	0	416	88	4	943	0	0	7	0	0	1	0	0	0	0	0	1
k__Bacteria	p__Bacteroidetes	Unclassified	Unclassified	Unclassified	Unclassified	Unclassified	0	0	0	28	4	21	25	0	23	5	0	61	3	0	0	10	19	22	13
k__Bacteria	p__Firmicutes	c__Clostridia	o__Clostridiales	f__[Mogibacteriaceae]	Unclassified	Unclassified	0	0	0	1	2	0	2	0	0	0	1	0	0	0	0	0	0	1	0
k__Bacteria	p__Firmicutes	c__Clostridia	o__Clostridiales	f__Lachnospiraceae	Unclassified	Unclassified	0	0	0	52	0	6	0	26	0	0	0	45	0	0	0	0	2	0	0
k__Bacteria	p__Tenericutes	c__Mollicutes	o__RF39	Unclassified	Unclassified	Unclassified	0	0	0	4	0	0	0	0	0	0	0	0	0	0	0	0	0	0	0
k__Bacteria	p__Firmicutes	c__Clostridia	o__Clostridiales	f__Lachnospiraceae	g__Coprococcus	Unclassified	0	0	0	119	0	0	0	0	2	0	0	0	37	0	0	32	0	4	45
k__Bacteria	p__Bacteroidetes	c__Bacteroidia	o__Bacteroidales	f__[Paraprevotellaceae]	g__[Prevotella]	Unclassified	0	0	0	3	0	0	0	2	0	0	1	0	1	0	0	3	0	7	7
k__Bacteria	p__Firmicutes	c__Clostridia	o__Clostridiales	f__Ruminococcaceae	Unclassified	Unclassified	0	0	0	2	0	0	0	1	0	0	0	0	3	0	0	0	0	3	1
k__Bacteria	p__Bacteroidetes	c__Bacteroidia	o__Bacteroidales	f__Bacteroidaceae	g__Bacteroides	Unclassified	0	0	0	4	0	0	0	0	0	0	0	0	0	0	0	0	1	0	1
k__Bacteria	p__Firmicutes	c__Clostridia	o__Clostridiales	Unclassified	Unclassified	Unclassified	0	0	0	2	0	0	0	0	0	0	0	0	0	0	0	0	0	0	0
k__Bacteria	p__Spirochaetes	c__Spirochaetes	o__Spirochaetales	f__Spirochaetaceae	Unclassified	Unclassified	0	0	0	14	0	0	1	0	0	0	1	0	0	0	0	0	0	0	0

Copyright © 2020, Sociedade Brasileira de Genética

Level_1	Level_2	Level_3	Level_4	Level_5	Level_6	Level_7	ss10	sn15	sn16	ss19	sn20	ss21	ss25	sn26	sn27	ss28	ss2	ss30	sn31	sn35	sn36	sn5	ss6	ss8	sn9
k__Bacteria	p__Firmicutes	c__Clostridia	o__Clostridiales	f__Lachnospiraceae	g__[Ruminococcus]	s__gnavus	0	0	0	7	1	0	3	19	0	0	0	0	0	0	0	0	0	0	0
k__Bacteria	p__Proteobacteria	c__Alphaproteobacteria	o__RF32	Unclassified	Unclassified	Unclassified	0	0	0	6	22	48	48	36	26	36	0	0	88	294	309	0	22	0	0
k__Bacteria	p__Firmicutes	c__Clostridia	o__Clostridiales	Unclassified	Unclassified	Unclassified	0	0	0	1	0	0	0	0	0	0	1	0	0	0	0	0	0	2	1
k__Bacteria	p__Firmicutes	c__Clostridia	o__Clostridiales	f__Lachnospiraceae	Unclassified	Unclassified	0	0	0	3	0	0	0	2	0	0	0	0	0	0	1	0	0	0	0
k__Bacteria	p__Firmicutes	c__Clostridia	o__Clostridiales	f__Ruminococcaceae	Unclassified	Unclassified	0	0	0	2	44	0	0	0	18	0	14	0	0	0	0	0	0	0	62
k__Bacteria	p__Firmicutes	c__Clostridia	o__Clostridiales	Unclassified	Unclassified	Unclassified	0	0	0	13	0	0	0	0	0	0	0	0	0	0	0	0	0	0	0
k__Bacteria	p__Firmicutes	c__Clostridia	o__Clostridiales	f__Lachnospiraceae	Unclassified	Unclassified	0	0	0	1	0	0	0	0	0	1	2	0	0	0	0	0	2	4	1
k__Bacteria	p__Firmicutes	c__Clostridia	o__Clostridiales	f__Ruminococcaceae	g__Ruminococcus	Unclassified	0	0	0	8	1	0	0	0	0	1	0	0	0	8	1	0	0	0	0
k__Bacteria	p__Bacteroidetes	c__Bacteroidia	o__Bacteroidales	f__S24-7	Unclassified	Unclassified	0	0	0	25	3	0	20	100	0	15	53	0	0	33	93	8	0	70	58
k__Bacteria	p__Firmicutes	c__Clostridia	o__Clostridiales	Unclassified	Unclassified	Unclassified	0	0	0	2	12	0	0	0	606	0	0	67	0	78	0	0	0	1	3
k__Bacteria	p__Firmicutes	c__Clostridia	o__Clostridiales	f__Lachnospiraceae	Unclassified	Unclassified	0	0	0	4	0	0	3	1	0	0	0	1	0	0	0	0	1	3	0
k__Bacteria	p__Firmicutes	c__Clostridia	o__Clostridiales	Unclassified	Unclassified	Unclassified	0	0	0	3	0	0	20	0	0	0	1	0	0	0	1	0	0	0	0
k__Bacteria	p__Proteobacteria	c__Alphaproteobacteria	o__RF32	Unclassified	Unclassified	Unclassified	0	0	0	10	0	0	0	0	0	0	0	0	0	0	0	0	0	0	0
k__Bacteria	p__Tenericutes	c__Mollicutes	o__RF39	Unclassified	Unclassified	Unclassified	0	0	0	2	0	0	0	0	0	0	2	7	0	0	1	1	2	1	0

Copyright © 2020, Sociedade Brasileira de Genética

**Level_1**	**Level_2**	**Level_3**	**Level_4**	**Level_5**	**Level_6**	**Level_7**	**ss10**	**sn15**	**sn16**	**ss19**	**sn20**	**ss21**	**ss25**	**sn26**	**sn27**	**ss28**	**ss2**	**ss30**	**sn31**	**sn35**	**sn36**	**sn5**	**ss6**	**ss8**	**sn9**
k__Bacteria	p__Firmicutes	c__Clostridia	o__Clostridiales	Unclassified	Unclassified	Unclassified	0	0	0	5	0	0	0	0	0	0	0	0	25	0	0	0	0	7	0
k__Bacteria	p__Firmicutes	c__Clostridia	o__Clostridiales	f__Dehalobacteriaceae	g__Dehalobacterium	Unclassified	0	0	0	2	0	0	0	0	0	0	1	0	0	0	0	0	1	0	0
k__Bacteria	p__Firmicutes	c__Clostridia	o__Clostridiales	Unclassified	Unclassified	Unclassified	0	0	0	20	0	12	0	5	0	0	0	791	20	1	0	3	254	2	1
k__Bacteria	p__Bacteroidetes	c__Bacteroidia	o__Bacteroidales	f__Prevotellaceae	g__Prevotella	Unclassified	0	0	0	1	3	3	3	0	2	0	0	2	1	3	0	0	0	0	1
k__Bacteria	p__Firmicutes	c__Clostridia	o__Clostridiales	f__Lachnospiraceae	Unclassified	Unclassified	0	0	0	1	0	0	0	0	1	3	1	45	0	6	0	0	0	0	0
k__Bacteria	p__Bacteroidetes	c__Bacteroidia	o__Bacteroidales	Unclassified	Unclassified	Unclassified	0	0	0	8	1	35	0	0	0	0	0	0	25	2	0	0	3	8	0
k__Bacteria	p__Firmicutes	c__Clostridia	o__Clostridiales	f__Lachnospiraceae	Unclassified	Unclassified	0	0	0	3	0	0	0	0	0	0	0	1	3	0	0	3	0	9	1
k__Bacteria	p__Firmicutes	c__Clostridia	o__Clostridiales	f__Lachnospiraceae	Unclassified	Unclassified	0	0	0	1	0	0	0	2	0	0	0	0	0	0	0	0	0	0	0
k__Bacteria	p__Firmicutes	c__Clostridia	o__Clostridiales	f__Lachnospiraceae	Unclassified	Unclassified	0	0	0	6	0	0	0	0	0	0	0	2	3	0	0	2	3	5	2
k__Bacteria	p__Firmicutes	c__Clostridia	o__Clostridiales	Unclassified	Unclassified	Unclassified	0	0	0	2	0	0	0	7	0	0	1	0	0	0	0	0	0	7	0
k__Bacteria	p__Bacteroidetes	c__Bacteroidia	o__Bacteroidales	f__S24-7	Unclassified	Unclassified	0	0	0	1	0	1	0	0	0	0	0	3	0	0	0	0	1	0	0
k__Bacteria	p__Firmicutes	c__Clostridia	o__Clostridiales	f__Lachnospiraceae	Unclassified	Unclassified	0	0	0	2	2	0	6	4	0	0	0	3	0	0	1	0	0	0	0
k__Bacteria	Unclassified	Unclassified	Unclassified	Unclassified	Unclassified	Unclassified	0	0	0	2	0	0	0	0	0	0	0	1	0	1	0	0	0	0	0
k__Bacteria	p__Firmicutes	c__Clostridia	o__Clostridiales	f__Ruminococcaceae	Unclassified	Unclassified	0	0	0	6	0	0	0	0	0	0	0	0	0	0	0	0	0	1	0

Copyright © 2020, Sociedade Brasileira de Genética

**Level_1**	**Level_2**	**Level_3**	**Level_4**	**Level_5**	**Level_6**	**Level_7**	**ss10**	**sn15**	**sn16**	**ss19**	**sn20**	**ss21**	**ss25**	**sn26**	**sn27**	**ss28**	**ss2**	**ss30**	**sn31**	**sn35**	**sn36**	**sn5**	**ss6**	**ss8**	**sn9**
k__Bacteria	p__Firmicutes	c__Clostridia	o__Clostridiales	f__Ruminococcaceae	g__Oscillospira	Unclassified	0	0	0	5	0	0	5	0	0	0	0	0	0	0	0	0	0	0	0
k__Bacteria	p__Firmicutes	c__Clostridia	o__Clostridiales	Unclassified	Unclassified	Unclassified	0	0	0	5	0	0	46	0	0	0	2	0	12	0	0	0	3	17	82
k__Bacteria	Unclassified	Unclassified	Unclassified	Unclassified	Unclassified	Unclassified	0	0	0	1	0	0	0	0	2	0	0	2	0	2	4	0	0	1	0
k__Bacteria	p__Firmicutes	Unclassified	Unclassified	Unclassified	Unclassified	Unclassified	0	0	0	4	0	0	0	1	1	1	2	5	0	0	1	1	1	1	1
k__Bacteria	p__Firmicutes	c__Clostridia	o__Clostridiales	f__Ruminococcaceae	Unclassified	Unclassified	0	0	0	3	0	0	0	0	0	0	0	0	0	0	0	0	0	0	0
k__Bacteria	p__Firmicutes	c__Clostridia	o__Clostridiales	f__Lachnospiraceae	Unclassified	Unclassified	0	0	0	1	0	0	0	0	1	0	0	11	0	0	0	0	1	3	0
k__Bacteria	p__Bacteroidetes	c__Bacteroidia	o__Bacteroidales	f__Prevotellaceae	g__Prevotella	s__copri	0	0	0	1	1	0	0	4	0	0	0	0	0	0	0	0	0	0	0
k__Bacteria	p__Firmicutes	c__Clostridia	o__Clostridiales	f__[Mogibacteriaceae]	Unclassified	Unclassified	0	0	0	2	1	0	0	0	0	0	0	0	0	1	0	0	1	0	0
k__Bacteria	Unclassified	Unclassified	Unclassified	Unclassified	Unclassified	Unclassified	0	0	0	1	0	0	0	0	1	0	0	0	0	0	0	0	0	0	0
k__Bacteria	p__Firmicutes	c__Clostridia	o__Clostridiales	f__Ruminococcaceae	Unclassified	Unclassified	0	0	0	2	1	0	2	4	0	2	1	0	0	2	1	0	0	0	0
k__Bacteria	p__Firmicutes	c__Clostridia	o__Clostridiales	f__Lachnospiraceae	Unclassified	Unclassified	0	0	0	1	0	0	0	3	0	0	0	66	0	0	0	8	1	0	0
k__Bacteria	p__Firmicutes	c__Clostridia	o__Clostridiales	f__Ruminococcaceae	Unclassified	Unclassified	0	0	0	2	0	1	1	0	0	0	0	3	0	1	0	1	1	1	0
k__Bacteria	p__Tenericutes	c__Mollicutes	o__RF39	Unclassified	Unclassified	Unclassified	0	0	0	3	0	0	1	2	0	0	3	0	0	0	0	0	0	0	0
k__Bacteria	p__Firmicutes	c__Clostridia	o__Clostridiales	Unclassified	Unclassified	Unclassified	0	0	0	1	0	0	2	0	0	1	0	0	0	0	0	0	0	0	0

Copyright © 2020, Sociedade Brasileira de Genética

Level_1	Level_2	Level_3	Level_4	Level_5	Level_6	Level_7	ss10	sn15	sn16	ss19	sn20	ss21	ss25	sn26	sn27	ss28	ss2	ss30	sn31	sn35	sn36	sn5	ss6	ss8	sn9
k__Bacteria	p__Firmicutes	c__Clostridia	o__Clostridiales	Unclassified	Unclassified	Unclassified	0	0	0	3	0	0	0	2	0	0	0	1	0	0	0	0	0	0	0
k__Bacteria	p__Firmicutes	c__Clostridia	o__Clostridiales	f__Lachnospiraceae	Unclassified	Unclassified	0	0	0	2	9	0	2	0	0	0	0	2	0	0	0	0	0	0	0
k__Bacteria	p__Firmicutes	c__Clostridia	o__Clostridiales	Unclassified	Unclassified	Unclassified	0	0	0	1	0	0	0	0	4	0	0	1	0	3	0	6	0	0	1
k__Bacteria	p__Bacteroidetes	c__Bacteroidia	o__Bacteroidales	f__Prevotellaceae	g__Prevotella	s__copri	0	0	0	1	2	0	6	5	0	0	2	0	4	1	0	2	0	3	1
k__Bacteria	p__Firmicutes	c__Clostridia	o__Clostridiales	Unclassified	Unclassified	Unclassified	0	0	0	2	0	67	0	0	0	0	0	609	130	0	0	0	0	212	113
k__Bacteria	p__Firmicutes	c__Clostridia	o__Clostridiales	f__Lachnospiraceae	g__Coprococcus	s__catus	0	0	0	1	0	5	0	0	0	0	0	0	0	0	0	0	0	0	0
k__Bacteria	p__Proteobacteria	c__Epsilonproteobacteria	o__Campylobacterales	f__Helicobacteraceae	g__Flexispira	Unclassified	0	0	0	1	0	0	0	1	0	0	0	2	0	6	1	0	2	0	0
k__Bacteria	p__Firmicutes	c__Clostridia	o__Clostridiales	f__Ruminococcaceae	g__Ruminococcus	Unclassified	0	0	0	1	0	0	0	0	0	0	0	0	0	0	0	0	1	1	0
k__Bacteria	p__Firmicutes	c__Clostridia	o__Clostridiales	f__Lachnospiraceae	Unclassified	Unclassified	0	0	0	2	0	0	0	0	0	0	0	2	0	0	0	0	0	0	0
k__Bacteria	p__Firmicutes	c__Clostridia	o__Clostridiales	f__Ruminococcaceae	Unclassified	Unclassified	0	0	0	4	0	0	0	0	0	0	0	0	0	0	0	2	0	0	0
k__Bacteria	p__Firmicutes	c__Clostridia	o__Clostridiales	Unclassified	Unclassified	Unclassified	0	0	0	2	0	0	0	0	0	0	0	0	0	0	0	0	0	0	5
k__Bacteria	p__Firmicutes	c__Clostridia	o__Clostridiales	f__Ruminococcaceae	Unclassified	Unclassified	0	0	0	1	0	0	0	1	0	0	0	0	0	0	0	0	0	0	0
k__Bacteria	p__Proteobacteria	c__Deltaproteobacteria	o__Desulfovibrionales	f__Desulfovibrionaceae	Unclassified	Unclassified	0	0	0	1	0	0	1	0	0	0	0	0	0	0	0	0	2	0	0
k__Bacteria	p__Actinobacteria	c__Coriobacteriia	o__Coriobacteriales	f__Coriobacteriaceae	g__Adlercreutzia	Unclassified	0	0	0	1	0	0	0	0	0	1	1	0	0	0	0	0	0	0	0

Genetics and Molecular Biology 43, 1 (2020)
Copyright © 2020, Sociedade Brasileira de Genética

Level_1	Level_2	Level_3	Level_4	Level_5	Level_6	Level_7	ss10	sn15	sn16	ss19	sn20	ss21	ss25	sn26	sn27	ss28	ss2	ss30	sn31	sn35	sn36	sn5	ss6	ss8	sn9
k__Bacteria	p__Bacteroidetes	c__Bacteroidia	o__Bacteroidales	f__Rikenellaceae	Unclassified	Unclassified	0	0	0	1	1	0	0	0	0	0	0	0	0	0	0	0	0	0	4
k__Bacteria	p__Bacteroidetes	c__Bacteroidia	o__Bacteroidales	f__Prevotellaceae	g__Prevotella	Unclassified	0	0	0	1	1	2	4	1	3	0	0	0	0	2	1	4	2	3	1
k__Bacteria	Unclassified	Unclassified	Unclassified	Unclassified	Unclassified	Unclassified	0	0	0	1	0	0	0	0	0	0	0	0	0	0	0	0	0	0	2
k__Bacteria	p__Firmicutes	Unclassified	Unclassified	Unclassified	Unclassified	Unclassified	0	0	0	1	0	0	0	0	0	0	0	0	0	4	1	0	1	0	1
k__Bacteria	p__Bacteroidetes	c__Bacteroidia	o__Bacteroidales	f__Rikenellaceae	Unclassified	Unclassified	0	0	0	1	0	0	0	17	6	0	0	0	0	0	0	0	1	2	4
k__Bacteria	p__Spirochaetes	c__Spirochaetes	o__Spirochaetales	f__Spirochaetaceae	g__Treponema	Unclassified	0	0	0	1	2	1	2	28	0	7	2	4	0	7	3	2	1	1	1
k__Bacteria	p__Firmicutes	c__Clostridia	o__Clostridiales	f__Ruminococcaceae	g__Oscillospira	Unclassified	0	0	0	2	3	1	2	0	0	0	0	1	0	2	0	0	0	0	4
k__Bacteria	p__Firmicutes	c__Clostridia	o__Clostridiales	f__Lachnospiraceae	Unclassified	Unclassified	0	0	0	1	0	0	0	0	0	0	0	0	0	0	5	0	0	1	0
k__Bacteria	p__Tenericutes	c__Mollicutes	o__RF39	Unclassified	Unclassified	Unclassified	0	0	0	1	0	0	0	0	0	0	0	0	0	0	29	4	10	2	24
k__Bacteria	p__Proteobacteria	c__Betaproteobacteria	o__Burkholderiales	f__Alcaligenaceae	g__Sutterella	Unclassified	0	0	0	1	1	0	0	0	0	0	0	1	0	0	0	0	0	0	3
k__Bacteria	p__Firmicutes	c__Clostridia	o__Clostridiales	f__Ruminococcaceae	g__Ruminococcus	Unclassified	0	0	0	0	63	26	0	0	4	635	0	0	7	4	36	0	0	36	0
k__Bacteria	p__Bacteroidetes	c__Bacteroidia	o__Bacteroidales	f__[Paraprevotellaceae]	g__[Prevotella]	Unclassified	0	0	0	0	563	139	1	74	1286	1826	147	64	573	37	68	5	276	150	0
k__Bacteria	p__Bacteroidetes	c__Bacteroidia	o__Bacteroidales	f__S24-7	Unclassified	Unclassified	0	0	0	0	29	0	6	171	0	0	0	1	8	0	37	0	0	16	25
k__Bacteria	p__Firmicutes	c__Clostridia	o__Clostridiales	f__Ruminococcaceae	g__Butyricicoccus	s__pullicaecorum	0	0	0	0	20	0	0	0	0	0	0	0	0	0	0	0	0	4	9

Copyright © 2020, Sociedade Brasileira de Genética

**Level_1**	**Level_2**	**Level_3**	**Level_4**	**Level_5**	**Level_6**	**Level_7**	**ss10**	**sn15**	**sn16**	**ss19**	**sn20**	**ss21**	**ss25**	**sn26**	**sn27**	**ss28**	**ss2**	**ss30**	**sn31**	**sn35**	**sn36**	**sn5**	**ss6**	**ss8**	**sn9**
k__Bacteria	p__Bacteroidetes	c__Bacteroidia	o__Bacteroidales	f__S24-7	Unclassified	Unclassified	0	0	0	0	8	63	35	51	3	1	0	4	12	0	43	0	43	19	15
k__Bacteria	p__Firmicutes	c__Erysipelotrichi	o__Erysipelotrichales	f__Erysipelotrichaceae	g__[Eubacterium]	s__cylindroides	0	0	0	0	10	0	0	0	0	0	0	0	0	0	0	0	0	0	0
k__Bacteria	p__Tenericutes	c__Mollicutes	o__RF39	Unclassified	Unclassified	Unclassified	0	0	0	0	7	0	1	0	0	0	0	0	3	0	4	0	2	0	11
k__Bacteria	p__Proteobacteria	c__Alphaproteobacteria	o__RF32	Unclassified	Unclassified	Unclassified	0	0	0	0	2	0	0	0	0	0	0	0	0	0	0	0	0	0	0
k__Bacteria	p__Bacteroidetes	c__Bacteroidia	o__Bacteroidales	f__S24-7	Unclassified	Unclassified	0	0	0	0	6	0	4	7	0	0	2	0	0	0	0	0	0	0	27
k__Bacteria	p__Proteobacteria	Unclassified	Unclassified	Unclassified	Unclassified	Unclassified	0	0	0	0	2	12	0	1	0	0	0	28	2	8	0	1	0	2	0
k__Bacteria	p__Tenericutes	c__Mollicutes	o__RF39	Unclassified	Unclassified	Unclassified	0	0	0	0	3	0	0	0	0	0	0	0	0	0	0	0	0	0	0
k__Bacteria	p__Firmicutes	c__Clostridia	o__Clostridiales	Unclassified	Unclassified	Unclassified	0	0	0	0	1	2	1	0	1	2	0	3	0	0	0	0	0	0	0
k__Archaea	p__Euryarchaeota	c__Methanobacteria	o__Methanobacteriales	f__Methanobacteriaceae	g__Methanosphaera	Unclassified	0	0	0	0	2	0	2	2	0	2	0	0	0	2	0	0	0	0	0
k__Bacteria	p__Proteobacteria	c__Alphaproteobacteria	o__RF32	Unclassified	Unclassified	Unclassified	0	0	0	0	1	0	0	0	0	0	5	0	0	0	0	0	12	0	0
k__Bacteria	p__Bacteroidetes	c__Bacteroidia	o__Bacteroidales	f__S24-7	Unclassified	Unclassified	0	0	0	0	2	0	0	100	0	2	12	0	0	0	0	0	0	0	0
k__Bacteria	p__Bacteroidetes	c__Bacteroidia	o__Bacteroidales	f__Bacteroidaceae	g__Bacteroides	Unclassified	0	0	0	0	2	0	3	2	1	1	0	2	0	19	2	0	4	3	1
k__Bacteria	p__Firmicutes	c__Clostridia	o__Clostridiales	f__Ruminococcaceae	Unclassified	Unclassified	0	0	0	0	3	1	0	0	0	0	0	1	2	0	0	0	0	6	3
k__Bacteria	Unclassified	Unclassified	Unclassified	Unclassified	Unclassified	Unclassified	0	0	0	0	2	2	0	0	2	0	2	0	0	5	1	1	3	4	0

Copyright © 2020, Sociedade Brasileira de Genética

Level_1	Level_2	Level_3	Level_4	Level_5	Level_6	Level_7	ss10	sn15	sn16	ss19	sn20	ss21	ss25	sn26	sn27	ss28	ss2	ss30	sn31	sn35	sn36	sn5	ss6	ss8	sn9
k__Bacteria	p__Firmicutes	c__Clostridia	o__Clostridiales	f__Ruminococcaceae	g__Faecalibacterium	Unclassified	0	0	0	0	3	0	2	0	0	4	0	0	0	0	0	0	0	0	0
k__Bacteria	p__Firmicutes	c__Clostridia	o__Clostridiales	Unclassified	Unclassified	Unclassified	0	0	0	0	1	0	0	0	1	0	0	0	0	1	1	0	0	1	0
k__Bacteria	p__Firmicutes	c__Clostridia	o__Clostridiales	f__Ruminococcaceae	Unclassified	Unclassified	0	0	0	0	2	1	0	0	2	3	1	2	1	6	2	3	3	2	1
k__Bacteria	p__Bacteroidetes	c__Bacteroidia	o__Bacteroidales	Unclassified	Unclassified	Unclassified	0	0	0	0	1	2	0	0	0	0	1	0	0	0	0	1	0	0	3
k__Bacteria	p__Firmicutes	c__Clostridia	o__Clostridiales	f__Lachnospiraceae	Unclassified	Unclassified	0	0	0	0	2	1	0	1	5	0	0	0	0	11	3	8	0	0	0
k__Bacteria	p__Firmicutes	c__Clostridia	o__Clostridiales	f__Lachnospiraceae	Unclassified	Unclassified	0	0	0	0	1	5	9	0	0	0	0	0	0	0	2	0	0	0	0
k__Bacteria	Unclassified	Unclassified	Unclassified	Unclassified	Unclassified	Unclassified	0	0	0	0	1	0	2	0	0	0	0	0	0	0	0	0	0	6	0
k__Bacteria	p__Lentisphaerae	c__[Lentisphaeria]	o__Victivallales	f__Victivallaceae	g__Victivallis	s__vadensis	0	0	0	0	1	0	0	0	0	0	0	5	0	0	0	1	1	3	0
k__Bacteria	Unclassified	Unclassified	Unclassified	Unclassified	Unclassified	Unclassified	0	0	0	0	1	0	0	0	0	3	0	0	0	0	0	0	0	0	0
k__Bacteria	Unclassified	Unclassified	Unclassified	Unclassified	Unclassified	Unclassified	0	0	0	0	2	2	0	0	1	0	0	13	0	0	0	2	2	2	5
k__Bacteria	Unclassified	Unclassified	Unclassified	Unclassified	Unclassified	Unclassified	0	0	0	0	1	0	0	0	0	0	0	0	0	0	0	0	0	3	1
k__Bacteria	p__Firmicutes	c__Clostridia	o__Clostridiales	f__Ruminococcaceae	g__Ruminococcus	Unclassified	0	0	0	0	2	3	2	2	2	1	3	4	0	1	1	1	0	0	0
k__Bacteria	Unclassified	Unclassified	Unclassified	Unclassified	Unclassified	Unclassified	0	0	0	0	3	0	2	0	0	0	0	0	0	0	0	0	0	1	0
k__Bacteria	p__Firmicutes	c__Clostridia	o__Clostridiales	f__Lachnospiraceae	Unclassified	Unclassified	0	0	0	0	1	1	0	1	2	1	0	7	0	1	0	1	0	5	4

Copyright © 2020, Sociedade Brasileira de Genética

Level_1	Level_2	Level_3	Level_4	Level_5	Level_6	Level_7	ss10	sn15	sn16	ss19	sn20	ss21	ss25	sn26	sn27	ss28	ss2	ss30	sn31	sn35	sn36	sn5	ss6	ss8	sn9
k__Bacteria	p__Firmicutes	c__Bacilli	o__Lactobacillales	f__Aerococcaceae	g__Facklamia	Unclassified	0	0	0	0	1	0	0	0	0	1	0	0	1	0	0	1	0	0	1
k__Bacteria	p__Bacteroidetes	c__Bacteroidia	o__Bacteroidales	f__Prevotellaceae	g__Prevotella	Unclassified	0	0	0	0	1	1	0	1	0	2	0	0	1	1	1	1	0	1	1
k__Bacteria	p__Firmicutes	c__Clostridia	o__Clostridiales	f__Christensenellaceae	Unclassified	Unclassified	0	0	0	0	1	0	0	0	0	0	0	0	0	0	2	0	1	1	0
k__Bacteria	p__Firmicutes	c__Bacilli	o__Lactobacillales	f__Aerococcaceae	Unclassified	Unclassified	0	0	0	0	1	0	0	0	0	0	0	0	0	0	0	0	0	0	1
k__Bacteria	p__Firmicutes	c__Clostridia	o__Clostridiales	Unclassified	Unclassified	Unclassified	0	0	0	0	2	0	0	0	0	0	0	42	0	0	1	0	0	1	0
k__Bacteria	p__Bacteroidetes	c__Bacteroidia	o__Bacteroidales	f__[Paraprevotellaceae]	g__[Prevotella]	Unclassified	0	0	0	0	1	0	0	0	0	0	0	1	0	0	0	0	0	1	0
k__Bacteria	p__Firmicutes	c__Clostridia	o__Clostridiales	f__Veillonellaceae	Unclassified	Unclassified	0	0	0	0	1	0	0	0	0	0	0	0	0	4	0	0	0	0	0
k__Bacteria	p__Actinobacteria	c__Coriobacteriia	o__Coriobacteriales	f__Coriobacteriaceae	g__Adlercreutzia	Unclassified	0	0	0	0	1	0	0	1	0	0	0	1	0	0	0	0	0	0	0
k__Bacteria	p__Bacteroidetes	c__Bacteroidia	o__Bacteroidales	Unclassified	Unclassified	Unclassified	0	0	0	0	0	72	0	0	4	0	0	123	24	0	0	0	0	0	0
k__Bacteria	p__Firmicutes	c__Clostridia	o__Clostridiales	f__Lachnospiraceae	g__Coprococcus	Unclassified	0	0	0	0	0	11	0	0	0	0	0	0	0	0	0	0	0	0	0
k__Bacteria	p__Spirochaetes	c__Spirochaetes	o__Spirochaetales	f__Spirochaetaceae	g__Treponema	Unclassified	0	0	0	0	0	170	0	0	183	0	152	114	57	0	0	185	0	1	0
k__Bacteria	p__Firmicutes	c__Clostridia	o__Clostridiales	f__Lachnospiraceae	g__Coprococcus	s__eutactus	0	0	0	0	0	138	0	0	0	0	0	0	0	0	0	0	0	0	0
k__Bacteria	p__Bacteroidetes	c__Bacteroidia	o__Bacteroidales	f__Prevotellaceae	Unclassified	Unclassified	0	0	0	0	0	58	0	0	0	0	0	0	3	0	0	338	0	933	858
k__Bacteria	p__Bacteroidetes	Unclassified	Unclassified	Unclassified	Unclassified	Unclassified	0	0	0	0	0	21	0	0	267	0	0	6	81	0	0	0	0	0	0

Genetics and Molecular Biology 43, 1 (2020)
Copyright © 2020, Sociedade Brasileira de Genética

Level_1	Level_2	Level_3	Level_4	Level_5	Level_6	Level_7	ss10	sn15	sn16	ss19	sn20	ss21	ss25	sn26	sn27	ss28	ss2	ss30	sn31	sn35	sn36	sn5	ss6	ss8	sn9
k__Bacteria	p__Firmicutes	c__Clostridia	o__Clostridiales	f__Lachnospiraceae	Unclassified	Unclassified	0	0	0	0	0	14	0	0	0	0	0	0	0	0	1	8	0	0	0
k__Bacteria	p__Elusimicrobia	c__Elusimicrobia	o__Elusimicrobiales	f__Elusimicrobiaceae	g__Elusimicrobium	Unclassified	0	0	0	0	0	47	55	24	0	25	0	0	0	0	0	109	109	527	61
k__Bacteria	p__Firmicutes	c__Clostridia	o__Clostridiales	f__Lachnospiraceae	Unclassified	Unclassified	0	0	0	0	0	1	102	8	1	0	24	4	0	0	1	0	0	2	0
k__Bacteria	p__Firmicutes	c__Clostridia	o__Clostridiales	f__Lachnospiraceae	Unclassified	Unclassified	0	0	0	0	0	7	0	0	2	0	0	0	0	0	0	0	0	0	0
k__Bacteria	p__Bacteroidetes	c__Bacteroidia	o__Bacteroidales	f__Prevotellaceae	g__Prevotella	Unclassified	0	0	0	0	0	21	0	0	0	0	0	0	0	0	0	0	0	0	0
k__Bacteria	p__Firmicutes	c__Clostridia	o__Clostridiales	f__Lachnospiraceae	Unclassified	Unclassified	0	0	0	0	0	5	0	0	0	0	30	0	0	0	8	1	0	9	0
k__Bacteria	p__Tenericutes	c__Mollicutes	o__RF39	Unclassified	Unclassified	Unclassified	0	0	0	0	0	4	0	0	0	13	1	0	7	0	6	1	0	0	0
k__Bacteria	p__Firmicutes	c__Clostridia	o__Clostridiales	f__Ruminococcaceae	g__Oscillospira	Unclassified	0	0	0	0	0	4	0	0	0	0	0	17	0	0	0	0	0	0	0
k__Bacteria	p__Firmicutes	Unclassified	Unclassified	Unclassified	Unclassified	Unclassified	0	0	0	0	0	3	0	0	2	0	0	0	0	0	0	0	0	0	0
k__Bacteria	p__Firmicutes	c__Clostridia	o__Clostridiales	Unclassified	Unclassified	Unclassified	0	0	0	0	0	1	0	2	3	3	0	1	9	0	1	4	2	2	0
k__Bacteria	p__Firmicutes	c__Clostridia	o__Clostridiales	f__Ruminococcaceae	Unclassified	Unclassified	0	0	0	0	0	1	0	0	8	0	0	4	1	1	1	2	4	0	0
k__Bacteria	p__Tenericutes	c__Mollicutes	o__RF39	Unclassified	Unclassified	Unclassified	0	0	0	0	0	22	0	0	0	0	0	0	0	0	0	0	0	0	0
k__Bacteria	p__Firmicutes	c__Clostridia	o__Clostridiales	f__Lachnospiraceae	Unclassified	Unclassified	0	0	0	0	0	7	40	0	21	3	0	0	0	3	11	0	0	0	0
k__Bacteria	p__Proteobacteria	c__Alphaproteobacteria	o__RF32	Unclassified	Unclassified	Unclassified	0	0	0	0	0	8	0	0	0	0	0	0	0	0	0	0	0	0	0

Copyright © 2020, Sociedade Brasileira de Genética

Level_1	Level_2	Level_3	Level_4	Level_5	Level_6	Level_7	ss10	sn15	sn16	ss19	sn20	ss21	ss25	sn26	sn27	ss28	ss2	ss30	sn31	sn35	sn36	sn5	ss6	ss8	sn9
k__Bacteria	p__Firmicutes	c__Clostridia	o__Clostridiales	f__Ruminococcaceae	Unclassified	Unclassified	0	0	0	0	0	3	1	0	4	0	0	5	0	0	0	0	0	0	0
k__Bacteria	p__Firmicutes	c__Clostridia	o__Clostridiales	f__Ruminococcaceae	Unclassified	Unclassified	0	0	0	0	0	3	0	0	9	0	0	2	0	0	0	0	0	0	0
k__Bacteria	Unclassified	Unclassified	Unclassified	Unclassified	Unclassified	Unclassified	0	0	0	0	0	2	0	0	4	0	0	20	2	0	0	0	0	0	0
k__Bacteria	p__Bacteroidetes	c__Bacteroidia	o__Bacteroidales	f__RF16	Unclassified	Unclassified	0	0	0	0	0	1	2	0	2	0	0	0	2	0	2	0	0	1	0
k__Bacteria	p__Firmicutes	c__Clostridia	o__Clostridiales	f__[Mogibacteriaceae]	Unclassified	Unclassified	0	0	0	0	0	4	0	0	2	0	0	0	0	0	0	3	0	0	0
k__Bacteria	p__Firmicutes	c__Clostridia	o__Clostridiales	f__Ruminococcaceae	Unclassified	Unclassified	0	0	0	0	0	2	10	1	0	0	0	0	0	0	0	0	3	0	14
k__Bacteria	p__Bacteroidetes	c__Bacteroidia	o__Bacteroidales	f__S24-7	Unclassified	Unclassified	0	0	0	0	0	2	2	1	0	0	0	0	0	0	2	0	0	2	0
k__Bacteria	p__Firmicutes	c__Clostridia	o__Clostridiales	f__Lachnospiraceae	Unclassified	Unclassified	0	0	0	0	0	1	0	0	4	0	0	0	0	0	0	2	0	0	6
k__Bacteria	p__Bacteroidetes	Unclassified	Unclassified	Unclassified	Unclassified	Unclassified	0	0	0	0	0	2	0	0	2	0	0	0	7	0	0	0	0	0	0
k__Bacteria	p__Bacteroidetes	c__Bacteroidia	o__Bacteroidales	Unclassified	Unclassified	Unclassified	0	0	0	0	0	1	3	0	0	0	0	0	4	1	1	0	1	0	0
k__Bacteria	p__Bacteroidetes	c__Bacteroidia	o__Bacteroidales	Unclassified	Unclassified	Unclassified	0	0	0	0	0	2	0	1	1	0	0	0	0	8	3	2	0	1	1
k__Bacteria	p__Firmicutes	c__Clostridia	o__Clostridiales	f__Ruminococcaceae	Unclassified	Unclassified	0	0	0	0	0	1	0	0	1	0	0	0	0	0	0	0	0	0	1
k__Bacteria	p__Firmicutes	c__Clostridia	o__Clostridiales	f__Lachnospiraceae	Unclassified	Unclassified	0	0	0	0	0	2	0	0	0	0	0	4	0	0	0	0	0	0	0
k__Bacteria	p__Bacteroidetes	c__Bacteroidia	o__Bacteroidales	Unclassified	Unclassified	Unclassified	0	0	0	0	0	1	0	0	0	1	0	0	0	0	0	0	0	0	0

Copyright © 2020, Sociedade Brasileira de Genética

**Level_1**	**Level_2**	**Level_3**	**Level_4**	**Level_5**	**Level_6**	**Level_7**	**ss10**	**sn15**	**sn16**	**ss19**	**sn20**	**ss21**	**ss25**	**sn26**	**sn27**	**ss28**	**ss2**	**ss30**	**sn31**	**sn35**	**sn36**	**sn5**	**ss6**	**ss8**	**sn9**
k__Bacteria	p__Firmicutes	c__Clostridia	o__Clostridiales	f__Peptococcaceae	Unclassified	Unclassified	0	0	0	0	0	1	0	0	0	0	0	0	1	0	0	1	0	0	0
k__Bacteria	p__Bacteroidetes	c__Bacteroidia	o__Bacteroidales	f__S24-7	Unclassified	Unclassified	0	0	0	0	0	1	0	0	0	0	0	1	0	0	0	1	0	0	1
k__Bacteria	p__Bacteroidetes	c__Bacteroidia	o__Bacteroidales	f__S24-7	Unclassified	Unclassified	0	0	0	0	0	1	0	0	1	0	0	0	0	4	1	3	0	1	0
k__Bacteria	p__Bacteroidetes	c__Bacteroidia	o__Bacteroidales	f__S24-7	Unclassified	Unclassified	0	0	0	0	0	0	25	39	0	0	0	0	0	28	0	0	0	0	0
k__Bacteria	p__Firmicutes	c__Clostridia	o__Clostridiales	f__Ruminococcaceae	g__Ruminococcus	s__flavefaciens	0	0	0	0	0	0	79	0	2	7	0	0	0	17	0	0	0	5	1
k__Bacteria	p__Firmicutes	c__Clostridia	o__Clostridiales	Unclassified	Unclassified	Unclassified	0	0	0	0	0	0	42	0	0	0	0	0	0	0	0	0	0	0	0
k__Bacteria	p__Cyanobacteria	c__4C0d-2	o__YS2	Unclassified	Unclassified	Unclassified	0	0	0	0	0	0	8	0	0	0	0	0	0	0	0	0	0	0	0
k__Bacteria	p__Tenericutes	c__Mollicutes	o__RF39	Unclassified	Unclassified	Unclassified	0	0	0	0	0	0	2	0	0	3	2	0	0	0	0	0	0	0	0
k__Bacteria	p__Cyanobacteria	c__4C0d-2	o__YS2	Unclassified	Unclassified	Unclassified	0	0	0	0	0	0	6	0	0	0	0	0	0	0	0	0	0	0	0
k__Bacteria	p__Firmicutes	c__Bacilli	o__Lactobacillales	f__Lactobacillaceae	g__Lactobacillus	Unclassified	0	0	0	0	0	0	7	0	0	2	4	0	0	0	0	0	0	0	0
k__Bacteria	p__Firmicutes	c__Clostridia	o__Clostridiales	f__Ruminococcaceae	Unclassified	Unclassified	0	0	0	0	0	0	1	4	0	0	0	4	0	0	0	0	2	0	0
k__Bacteria	Unclassified	Unclassified	Unclassified	Unclassified	Unclassified	Unclassified	0	0	0	0	0	0	2	0	0	0	0	0	0	1	0	0	0	1	0
k__Bacteria	p__Tenericutes	c__Mollicutes	o__RF39	Unclassified	Unclassified	Unclassified	0	0	0	0	0	0	3	4	0	0	0	0	0	0	0	2	0	0	0
k__Bacteria	p__Firmicutes	c__Clostridia	o__Clostridiales	Unclassified	Unclassified	Unclassified	0	0	0	0	0	0	7	0	2	0	3	0	0	0	0	0	0	16	0

Copyright © 2020, Sociedade Brasileira de Genética

Level_1	Level_2	Level_3	Level_4	Level_5	Level_6	Level_7	ss10	sn15	sn16	ss19	sn20	ss21	ss25	sn26	sn27	ss28	ss2	ss30	sn31	sn35	sn36	sn5	ss6	ss8	sn9
k__Bacteria	p__Firmicutes	c__Bacilli	o__Lactobacillales	Unclassified	Unclassified	Unclassified	0	0	0	0	0	0	1	0	0	0	0	0	0	3	0	0	0	0	0
k__Bacteria	p__Firmicutes	c__Clostridia	o__Clostridiales	f__Lachnospiraceae	Unclassified	Unclassified	0	0	0	0	0	0	2	0	0	0	0	0	0	0	0	0	0	0	0
k__Bacteria	Unclassified	Unclassified	Unclassified	Unclassified	Unclassified	Unclassified	0	0	0	0	0	0	2	0	0	0	0	0	0	0	0	0	0	0	0
k__Bacteria	p__Firmicutes	c__Clostridia	o__Clostridiales	f__Dehalobacteriaceae	g__Dehalobacterium	Unclassified	0	0	0	0	0	0	3	0	0	0	0	0	0	0	0	0	2	3	0
k__Bacteria	p__Bacteroidetes	c__Bacteroidia	o__Bacteroidales	f__S24-7	Unclassified	Unclassified	0	0	0	0	0	0	1	3	0	1	0	0	0	0	0	0	0	0	0
k__Bacteria	p__Firmicutes	c__Clostridia	o__Clostridiales	Unclassified	Unclassified	Unclassified	0	0	0	0	0	0	2	0	0	0	0	1	0	1	0	0	0	0	0
k__Bacteria	p__Firmicutes	c__Clostridia	o__Clostridiales	f__Ruminococcaceae	g__Oscillospira	Unclassified	0	0	0	0	0	0	1	0	0	1	0	0	0	0	2	0	0	1	0
k__Bacteria	p__Bacteroidetes	c__Bacteroidia	o__Bacteroidales	Unclassified	Unclassified	Unclassified	0	0	0	0	0	0	1	0	1	0	0	0	0	0	0	0	0	0	1
k__Bacteria	p__Bacteroidetes	c__Bacteroidia	o__Bacteroidales	f__S24-7	Unclassified	Unclassified	0	0	0	0	0	0	1	1	0	1	1	0	0	0	0	1	0	1	0
k__Bacteria	p__Firmicutes	c__Clostridia	o__Clostridiales	Unclassified	Unclassified	Unclassified	0	0	0	0	0	0	2	0	0	16	0	1	12	3	0	0	0	15	4
k__Bacteria	p__Firmicutes	c__Erysipelotrichi	o__Erysipelotrichales	f__Erysipelotrichaceae	Unclassified	Unclassified	0	0	0	0	0	0	1	5	0	0	0	0	0	0	0	0	0	0	0
k__Bacteria	p__Firmicutes	c__Erysipelotrichi	o__Erysipelotrichales	f__Erysipelotrichaceae	g__Coprobacillus	Unclassified	0	0	0	0	0	0	1	4	0	0	0	0	0	0	0	0	0	0	0
k__Bacteria	p__TM7	c__TM7-3	o__CW040	f__F16	Unclassified	Unclassified	0	0	0	0	0	0	1	0	1	0	0	0	1	2	1	0	0	0	1
k__Bacteria	p__Firmicutes	c__Clostridia	o__Clostridiales	f__Ruminococcaceae	Unclassified	Unclassified	0	0	0	0	0	0	2	0	9	0	0	0	1	0	0	0	0	0	0

Copyright © 2020, Sociedade Brasileira de Genética

**Level_1**	**Level_2**	**Level_3**	**Level_4**	**Level_5**	**Level_6**	**Level_7**	**ss10**	**sn15**	**sn16**	**ss19**	**sn20**	**ss21**	**ss25**	**sn26**	**sn27**	**ss28**	**ss2**	**ss30**	**sn31**	**sn35**	**sn36**	**sn5**	**ss6**	**ss8**	**sn9**
k__Bacteria	Unclassified	Unclassified	Unclassified	Unclassified	Unclassified	Unclassified	0	0	0	0	0	0	1	0	0	0	0	0	0	0	1	0	0	0	0
k__Bacteria	p__Bacteroidetes	c__Bacteroidia	o__Bacteroidales	f__S24-7	Unclassified	Unclassified	0	0	0	0	0	0	0	138	0	0	0	0	0	6	0	0	0	0	0
k__Bacteria	p__Bacteroidetes	c__Bacteroidia	o__Bacteroidales	f__S24-7	Unclassified	Unclassified	0	0	0	0	0	0	0	66	0	0	0	0	0	0	0	0	0	0	0
k__Bacteria	p__Proteobacteria	c__Alphaproteobacteria	o__RF32	Unclassified	Unclassified	Unclassified	0	0	0	0	0	0	0	46	0	0	0	0	0	0	0	0	0	0	0
k__Bacteria	p__Firmicutes	c__Clostridia	o__Clostridiales	f__Lachnospiraceae	Unclassified	Unclassified	0	0	0	0	0	0	0	55	0	0	14	0	0	177	0	0	0	0	5
k__Bacteria	p__Proteobacteria	c__Alphaproteobacteria	o__RF32	Unclassified	Unclassified	Unclassified	0	0	0	0	0	0	0	11	0	0	0	0	0	0	0	0	1	0	0
k__Bacteria	p__Bacteroidetes	c__Bacteroidia	o__Bacteroidales	Unclassified	Unclassified	Unclassified	0	0	0	0	0	0	0	1	0	0	0	0	0	2	0	0	0	0	0
k__Bacteria	p__Firmicutes	c__Clostridia	o__Clostridiales	f__Ruminococcaceae	Unclassified	Unclassified	0	0	0	0	0	0	0	10	0	0	0	0	0	0	0	0	21	2	0
k__Bacteria	Unclassified	Unclassified	Unclassified	Unclassified	Unclassified	Unclassified	0	0	0	0	0	0	0	7	0	0	0	0	0	0	0	29	18	0	0
k__Bacteria	p__Firmicutes	c__Clostridia	o__Clostridiales	f__Ruminococcaceae	Unclassified	Unclassified	0	0	0	0	0	0	0	2	0	0	1	0	0	0	0	1	0	0	0
k__Bacteria	p__Firmicutes	c__Clostridia	o__Clostridiales	f__Lachnospiraceae	Unclassified	Unclassified	0	0	0	0	0	0	0	4	0	0	0	0	0	1	0	0	0	0	0
k__Bacteria	p__Bacteroidetes	c__Bacteroidia	o__Bacteroidales	f__[Paraprevotellaceae]	g__[Prevotella]	Unclassified	0	0	0	0	0	0	0	1	0	0	0	0	1	1	0	0	0	0	1
k__Bacteria	p__Firmicutes	c__Clostridia	o__Clostridiales	f__Ruminococcaceae	g__Ruminococcus	Unclassified	0	0	0	0	0	0	0	8	0	0	0	0	0	0	0	0	0	0	0
k__Bacteria	p__Cyanobacteria	c__4C0d-2	o__YS2	Unclassified	Unclassified	Unclassified	0	0	0	0	0	0	0	3	0	0	0	0	0	0	0	0	0	0	0

Copyright © 2020, Sociedade Brasileira de Genética

Level_1	Level_2	Level_3	Level_4	Level_5	Level_6	Level_7	ss10	sn15	sn16	ss19	sn20	ss21	ss25	sn26	sn27	ss28	ss2	ss30	sn31	sn35	sn36	sn5	ss6	ss8	sn9
k__Bacteria	p__Firmicutes	c__Clostridia	o__Clostridiales	f__Ruminococcaceae	Unclassified	Unclassified	0	0	0	0	0	0	0	1	0	0	0	0	0	0	0	0	1	0	0
k__Bacteria	p__Bacteroidetes	c__Bacteroidia	o__Bacteroidales	f__Prevotellaceae	g__Prevotella	s__copri	0	0	0	0	0	0	0	2	0	0	0	0	0	0	0	0	0	0	0
k__Bacteria	p__Firmicutes	c__Clostridia	o__Clostridiales	f__Lachnospiraceae	g__Coprococcus	Unclassified	0	0	0	0	0	0	0	0	120	0	0	0	0	0	0	0	0	0	0
k__Bacteria	p__TM7	c__TM7-3	o__CW040	f__F16	Unclassified	Unclassified	0	0	0	0	0	0	0	0	2	0	3	0	1	0	6	0	2	0	0
k__Bacteria	p__Firmicutes	c__Clostridia	o__Clostridiales	f__Ruminococcaceae	g__Oscillospira	Unclassified	0	0	0	0	0	0	0	0	4	0	0	0	0	5	0	0	0	0	0
k__Bacteria	p__Firmicutes	c__Clostridia	o__Clostridiales	f__Clostridiaceae	g__Candidatus Arthromitus	Unclassified	0	0	0	0	0	0	0	0	10	0	0	0	0	0	0	0	0	0	0
k__Bacteria	p__Firmicutes	c__Clostridia	o__Clostridiales	Unclassified	Unclassified	Unclassified	0	0	0	0	0	0	0	0	1	0	1	0	1	5	0	0	0	0	0
k__Bacteria	p__Proteobacteria	c__Alphaproteobacteria	o__RF32	Unclassified	Unclassified	Unclassified	0	0	0	0	0	0	0	0	7	4	0	0	0	0	12	0	8	0	0
k__Bacteria	p__Bacteroidetes	c__Bacteroidia	o__Bacteroidales	f__Prevotellaceae	g__Prevotella	Unclassified	0	0	0	0	0	0	0	0	1	0	0	0	0	1	3	0	0	332	1
k__Bacteria	p__Proteobacteria	c__Alphaproteobacteria	Unclassified	Unclassified	Unclassified	Unclassified	0	0	0	0	0	0	0	0	1	0	0	0	0	0	8	1	8	2	0
k__Bacteria	p__Firmicutes	c__Clostridia	o__Clostridiales	f__Dehalobacteriaceae	g__Dehalobacterium	Unclassified	0	0	0	0	0	0	0	0	7	0	3	0	0	0	0	0	2	2	0
k__Bacteria	p__Firmicutes	c__Erysipelotrichi	o__Erysipelotrichales	f__Erysipelotrichaceae	g__Allobaculum	Unclassified	0	0	0	0	0	0	0	0	7	0	0	0	3	0	0	0	14	2	3
k__Bacteria	p__Tenericutes	c__Mollicutes	o__RF39	Unclassified	Unclassified	Unclassified	0	0	0	0	0	0	0	0	1	0	0	0	0	2	0	0	0	0	0
k__Bacteria	p__Bacteroidetes	c__Bacteroidia	o__Bacteroidales	f__Prevotellaceae	g__Prevotella	Unclassified	0	0	0	0	0	0	0	0	3	0	0	1	0	0	0	0	0	0	0

Copyright © 2020, Sociedade Brasileira de Genética

Level_1	Level_2	Level_3	Level_4	Level_5	Level_6	Level_7	ss10	sn15	sn16	ss19	sn20	ss21	ss25	sn26	sn27	ss28	ss2	ss30	sn31	sn35	sn36	sn5	ss6	ss8	sn9
k__Bacteria	p__Firmicutes	c__Clostridia	Unclassified	Unclassified	Unclassified	Unclassified	0	0	0	0	0	0	0	0	1	0	0	1	2	0	0	0	0	0	0
k__Bacteria	p__Firmicutes	c__Clostridia	o__Clostridiales	f__Ruminococcaceae	Unclassified	Unclassified	0	0	0	0	0	0	0	0	1	0	0	0	0	0	5	3	0	18	12
k__Bacteria	p__Tenericutes	c__Mollicutes	o__RF39	Unclassified	Unclassified	Unclassified	0	0	0	0	0	0	0	0	1	2	3	0	0	0	3	0	0	0	0
k__Bacteria	p__Firmicutes	Unclassified	Unclassified	Unclassified	Unclassified	Unclassified	0	0	0	0	0	0	0	0	1	0	0	1	0	0	0	1	0	0	0
k__Bacteria	p__Firmicutes	c__Clostridia	o__Clostridiales	f__Ruminococcaceae	Unclassified	Unclassified	0	0	0	0	0	0	0	0	1	1	0	1	0	1	1	0	0	0	0
k__Bacteria	p__Bacteroidetes	c__Bacteroidia	o__Bacteroidales	f__Prevotellaceae	g__Prevotella	Unclassified	0	0	0	0	0	0	0	0	1	0	0	0	0	0	0	1	0	0	0
k__Bacteria	p__Tenericutes	c__Mollicutes	o__RF39	Unclassified	Unclassified	Unclassified	0	0	0	0	0	0	0	0	2	0	0	0	2	0	0	0	0	0	0
k__Bacteria	p__Tenericutes	c__Mollicutes	o__RF39	Unclassified	Unclassified	Unclassified	0	0	0	0	0	0	0	0	1	0	0	0	0	2	6	1	0	2	1
k__Bacteria	p__Firmicutes	c__Clostridia	o__Clostridiales	Unclassified	Unclassified	Unclassified	0	0	0	0	0	0	0	0	1	1	0	1	0	0	0	0	0	0	1
k__Bacteria	p__Firmicutes	c__Clostridia	o__Clostridiales	f__Lachnospiraceae	Unclassified	Unclassified	0	0	0	0	0	0	0	0	1	0	0	69	6	0	0	0	0	0	0
k__Bacteria	p__Bacteroidetes	c__Bacteroidia	o__Bacteroidales	f__Rikenellaceae	Unclassified	Unclassified	0	0	0	0	0	0	0	0	1	0	0	3	0	0	0	0	2	1	0
k__Bacteria	p__Firmicutes	c__Clostridia	o__Clostridiales	f__Ruminococcaceae	Unclassified	Unclassified	0	0	0	0	0	0	0	0	1	0	0	1	0	1	0	0	0	0	0
k__Bacteria	p__Actinobacteria	c__Coriobacteriia	o__Coriobacteriales	f__Coriobacteriaceae	g__Adlercreutzia	Unclassified	0	0	0	0	0	0	0	0	1	0	0	1	0	1	0	0	0	0	0
k__Bacteria	p__Tenericutes	c__Mollicutes	o__RF39	Unclassified	Unclassified	Unclassified	0	0	0	0	0	0	0	0	0	3	0	0	0	0	0	0	0	0	1

Copyright © 2020, Sociedade Brasileira de Genética

**Level_1**	**Level_2**	**Level_3**	**Level_4**	**Level_5**	**Level_6**	**Level_7**	**ss10**	**sn15**	**sn16**	**ss19**	**sn20**	**ss21**	**ss25**	**sn26**	**sn27**	**ss28**	**ss2**	**ss30**	**sn31**	**sn35**	**sn36**	**sn5**	**ss6**	**ss8**	**sn9**
k__Bacteria	p__Firmicutes	c__Erysipelotrichi	o__Erysipelotrichales	f__Erysipelotrichaceae	g__Allobaculum	Unclassified	0	0	0	0	0	0	0	0	0	2	0	0	0	0	0	0	0	0	0
k__Bacteria	p__Firmicutes	c__Clostridia	o__Clostridiales	f__[Mogibacteriaceae]	g__Mogibacterium	Unclassified	0	0	0	0	0	0	0	0	0	1	0	0	0	0	3	0	0	0	0
k__Bacteria	p__Firmicutes	c__Clostridia	o__Clostridiales	Unclassified	Unclassified	Unclassified	0	0	0	0	0	0	0	0	0	0	21	0	0	0	0	0	0	17	0
k__Bacteria	p__Firmicutes	c__Clostridia	o__Clostridiales	f__Ruminococcaceae	g__Ruminococcus	Unclassified	0	0	0	0	0	0	0	0	0	0	2	0	1	0	0	0	0	0	0
k__Bacteria	p__Cyanobacteria	c__4C0d-2	o__YS2	Unclassified	Unclassified	Unclassified	0	0	0	0	0	0	0	0	0	0	9	19	0	0	0	0	0	0	0
k__Bacteria	p__Firmicutes	c__Clostridia	o__Clostridiales	Unclassified	Unclassified	Unclassified	0	0	0	0	0	0	0	0	0	0	1	0	0	567	0	0	0	0	0
k__Bacteria	p__Firmicutes	c__Clostridia	o__Clostridiales	f__Ruminococcaceae	g__Oscillospira	Unclassified	0	0	0	0	0	0	0	0	0	0	3	0	0	0	0	2	0	0	0
k__Bacteria	p__Firmicutes	c__Clostridia	o__Clostridiales	f__Lachnospiraceae	Unclassified	Unclassified	0	0	0	0	0	0	0	0	0	0	1	0	0	0	1	0	0	0	0
k__Bacteria	p__Firmicutes	Unclassified	Unclassified	Unclassified	Unclassified	Unclassified	0	0	0	0	0	0	0	0	0	0	0	3	0	0	0	0	0	1	0
k__Bacteria	p__Firmicutes	c__Clostridia	o__Clostridiales	f__Ruminococcaceae	g__Ruminococcus	Unclassified	0	0	0	0	0	0	0	0	0	0	0	11	1	1	0	0	0	0	1
k__Bacteria	p__Firmicutes	c__Clostridia	o__Clostridiales	f__Ruminococcaceae	g__Oscillospira	Unclassified	0	0	0	0	0	0	0	0	0	0	0	8	0	0	0	5	1	1	0
k__Bacteria	p__Firmicutes	c__Clostridia	o__Clostridiales	f__Lachnospiraceae	Unclassified	Unclassified	0	0	0	0	0	0	0	0	0	0	0	16	0	0	0	0	0	4	0
k__Bacteria	p__Firmicutes	c__Clostridia	o__Clostridiales	f__Ruminococcaceae	Unclassified	Unclassified	0	0	0	0	0	0	0	0	0	0	0	2	0	0	0	0	0	0	0
k__Bacteria	p__Firmicutes	Unclassified	Unclassified	Unclassified	Unclassified	Unclassified	0	0	0	0	0	0	0	0	0	0	0	7	2	1	0	0	0	0	0

Copyright © 2020, Sociedade Brasileira de Genética

**Level_1**	**Level_2**	**Level_3**	**Level_4**	**Level_5**	**Level_6**	**Level_7**	**ss10**	**sn15**	**sn16**	**ss19**	**sn20**	**ss21**	**ss25**	**sn26**	**sn27**	**ss28**	**ss2**	**ss30**	**sn31**	**sn35**	**sn36**	**sn5**	**ss6**	**ss8**	**sn9**
k__Bacteria	p__Tenericutes	c__Mollicutes	o__RF39	Unclassified	Unclassified	Unclassified	0	0	0	0	0	0	0	0	0	0	0	2	0	0	0	0	0	0	2
k__Bacteria	p__Firmicutes	c__Clostridia	o__Clostridiales	Unclassified	Unclassified	Unclassified	0	0	0	0	0	0	0	0	0	0	0	2	2	0	0	0	1	0	0
k__Bacteria	p__Bacteroidetes	c__Bacteroidia	o__Bacteroidales	Unclassified	Unclassified	Unclassified	0	0	0	0	0	0	0	0	0	0	0	1	0	1	0	1	0	0	1
k__Bacteria	p__Firmicutes	c__Erysipelotrichi	o__Erysipelotrichales	f__Erysipelotrichaceae	Unclassified	Unclassified	0	0	0	0	0	0	0	0	0	0	0	1	0	0	0	1	0	0	0
k__Bacteria	p__Firmicutes	c__Erysipelotrichi	o__Erysipelotrichales	f__Erysipelotrichaceae	g__RFN20	Unclassified	0	0	0	0	0	0	0	0	0	0	0	1	0	1	0	0	0	0	0
k__Bacteria	p__Proteobacteria	c__Betaproteobacteria	Unclassified	Unclassified	Unclassified	Unclassified	0	0	0	0	0	0	0	0	0	0	0	1	0	0	0	0	1	0	0
k__Bacteria	p__Actinobacteria	c__Coriobacteriia	o__Coriobacteriales	f__Coriobacteriaceae	Unclassified	Unclassified	0	0	0	0	0	0	0	0	0	0	0	4	0	0	0	0	0	0	0
k__Bacteria	Unclassified	Unclassified	Unclassified	Unclassified	Unclassified	Unclassified	0	0	0	0	0	0	0	0	0	0	0	4	0	0	0	0	0	0	0
k__Bacteria	p__Firmicutes	c__Clostridia	o__Clostridiales	f__Lachnospiraceae	g__[Ruminococcus]	Unclassified	0	0	0	0	0	0	0	0	0	0	0	1	0	0	0	0	2	0	0
k__Bacteria	p__Firmicutes	c__Clostridia	o__Clostridiales	Unclassified	Unclassified	Unclassified	0	0	0	0	0	0	0	0	0	0	0	4	1	0	0	0	0	0	0
k__Bacteria	p__Bacteroidetes	c__Bacteroidia	o__Bacteroidales	f__[Paraprevotellaceae]	g__[Prevotella]	Unclassified	0	0	0	0	0	0	0	0	0	0	0	4	0	1	0	0	1	0	1
k__Bacteria	p__Firmicutes	c__Clostridia	o__Clostridiales	f__Ruminococcaceae	Unclassified	Unclassified	0	0	0	0	0	0	0	0	0	0	0	1	0	0	0	3	0	1	3
k__Bacteria	p__Firmicutes	c__Clostridia	o__Clostridiales	f__Ruminococcaceae	g__Ruminococcus	Unclassified	0	0	0	0	0	0	0	0	0	0	0	2	2	0	2	0	2	2	1
k__Bacteria	p__Firmicutes	c__Clostridia	o__Clostridiales	f__Ruminococcaceae	Unclassified	Unclassified	0	0	0	0	0	0	0	0	0	0	0	4	1	1	0	0	0	2	3

Copyright © 2020, Sociedade Brasileira de Genética

Level_1	Level_2	Level_3	Level_4	Level_5	Level_6	Level_7	ss10	sn15	sn16	ss19	sn20	ss21	ss25	sn26	sn27	ss28	ss2	ss30	sn31	sn35	sn36	sn5	ss6	ss8	sn9
k__Bacteria	p__Firmicutes	c__Clostridia	o__Clostridiales	Unclassified	Unclassified	Unclassified	0	0	0	0	0	0	0	0	0	0	0	3	0	0	0	0	0	0	0
k__Bacteria	p__Firmicutes	c__Clostridia	o__Clostridiales	f__Ruminococcaceae	Unclassified	Unclassified	0	0	0	0	0	0	0	0	0	0	0	1	1	0	0	0	3	0	0
k__Bacteria	Unclassified	Unclassified	Unclassified	Unclassified	Unclassified	Unclassified	0	0	0	0	0	0	0	0	0	0	0	1	0	0	0	0	1	0	0
k__Bacteria	p__Firmicutes	c__Clostridia	o__Clostridiales	Unclassified	Unclassified	Unclassified	0	0	0	0	0	0	0	0	0	0	0	1	1	1	0	0	1	0	0
k__Bacteria	p__Firmicutes	c__Clostridia	o__Clostridiales	f__Ruminococcaceae	Unclassified	Unclassified	0	0	0	0	0	0	0	0	0	0	0	1	0	1	1	0	0	0	0
k__Bacteria	p__Firmicutes	c__Clostridia	o__Clostridiales	Unclassified	Unclassified	Unclassified	0	0	0	0	0	0	0	0	0	0	0	0	2	0	0	0	0	0	0
k__Bacteria	p__Proteobacteria	c__Alphaproteobacteria	o__Rickettsiales	Unclassified	Unclassified	Unclassified	0	0	0	0	0	0	0	0	0	0	0	0	6	12	3	0	0	0	0
k__Bacteria	p__Firmicutes	c__Clostridia	o__Clostridiales	Unclassified	Unclassified	Unclassified	0	0	0	0	0	0	0	0	0	0	0	0	2	0	0	0	0	0	0
k__Bacteria	p__Actinobacteria	c__Actinobacteria	o__Actinomycetales	f__Actinomycetaceae	g__Actinomyces	Unclassified	0	0	0	0	0	0	0	0	0	0	0	0	1	0	0	1	0	0	2
k__Bacteria	p__Tenericutes	c__Mollicutes	o__RF39	Unclassified	Unclassified	Unclassified	0	0	0	0	0	0	0	0	0	0	0	0	2	0	0	0	0	0	0
k__Bacteria	p__Firmicutes	c__Clostridia	o__Clostridiales	Unclassified	Unclassified	Unclassified	0	0	0	0	0	0	0	0	0	0	0	0	1	0	0	4	0	7	0
k__Bacteria	Unclassified	Unclassified	Unclassified	Unclassified	Unclassified	Unclassified	0	0	0	0	0	0	0	0	0	0	0	0	1	0	0	0	0	2	0
k__Bacteria	p__Firmicutes	c__Clostridia	o__Clostridiales	Unclassified	Unclassified	Unclassified	0	0	0	0	0	0	0	0	0	0	0	0	1	0	0	0	0	5	0
k__Bacteria	p__Firmicutes	c__Clostridia	o__Clostridiales	f__Ruminococcaceae	Unclassified	Unclassified	0	0	0	0	0	0	0	0	0	0	0	0	1	0	0	1	0	0	0

Copyright © 2020, Sociedade Brasileira de Genética

**Level_1**	**Level_2**	**Level_3**	**Level_4**	**Level_5**	**Level_6**	**Level_7**	**ss10**	**sn15**	**sn16**	**ss19**	**sn20**	**ss21**	**ss25**	**sn26**	**sn27**	**ss28**	**ss2**	**ss30**	**sn31**	**sn35**	**sn36**	**sn5**	**ss6**	**ss8**	**sn9**
k__Bacteria	p__Fusobacteria	c__Fusobacteriia	o__Fusobacteriales	f__Fusobacteriaceae	Unclassified	Unclassified	0	0	0	0	0	0	0	0	0	0	0	0	1	3	0	3	0	0	0
k__Bacteria	p__Firmicutes	c__Clostridia	o__Clostridiales	f__Lachnospiraceae	g__Coprococcus	Unclassified	0	0	0	0	0	0	0	0	0	0	0	0	0	448	4	0	0	0	0
k__Bacteria	p__Firmicutes	Unclassified	Unclassified	Unclassified	Unclassified	Unclassified	0	0	0	0	0	0	0	0	0	0	0	0	0	10	0	0	0	0	0
k__Bacteria	p__Firmicutes	c__Clostridia	o__Clostridiales	Unclassified	Unclassified	Unclassified	0	0	0	0	0	0	0	0	0	0	0	0	0	37	0	0	0	0	8
k__Bacteria	p__Firmicutes	c__Clostridia	o__Clostridiales	f__Lachnospiraceae	Unclassified	Unclassified	0	0	0	0	0	0	0	0	0	0	0	0	0	2	0	0	0	0	0
k__Bacteria	p__Tenericutes	c__Mollicutes	o__RF39	Unclassified	Unclassified	Unclassified	0	0	0	0	0	0	0	0	0	0	0	0	0	19	0	0	0	0	0
k__Bacteria	p__Firmicutes	c__Clostridia	o__Clostridiales	f__Ruminococcaceae	g__Ruminococcus	Unclassified	0	0	0	0	0	0	0	0	0	0	0	0	0	1	0	1	0	0	3
k__Bacteria	Unclassified	Unclassified	Unclassified	Unclassified	Unclassified	Unclassified	0	0	0	0	0	0	0	0	0	0	0	0	0	6	0	1	0	0	0
k__Bacteria	Unclassified	Unclassified	Unclassified	Unclassified	Unclassified	Unclassified	0	0	0	0	0	0	0	0	0	0	0	0	0	2	0	0	0	0	0
k__Bacteria	p__Firmicutes	c__Clostridia	o__Clostridiales	f__Lachnospiraceae	Unclassified	Unclassified	0	0	0	0	0	0	0	0	0	0	0	0	0	6	0	0	0	0	0
k__Bacteria	p__Firmicutes	c__Clostridia	o__Clostridiales	f__[Tissierellaceae]	g__Anaerococcus	Unclassified	0	0	0	0	0	0	0	0	0	0	0	0	0	2	0	0	0	0	0
k__Bacteria	p__Firmicutes	c__Clostridia	o__Clostridiales	f__Eubacteriaceae	g__Anaerofustis	Unclassified	0	0	0	0	0	0	0	0	0	0	0	0	0	1	0	0	1	0	0
k__Bacteria	p__Firmicutes	c__Bacilli	o__Lactobacillales	f__Lactobacillaceae	g__Lactobacillus	Unclassified	0	0	0	0	0	0	0	0	0	0	0	0	0	3	0	0	0	0	1
k__Bacteria	p__Firmicutes	c__Bacilli	o__Lactobacillales	f__Lactobacillaceae	g__Lactobacillus	Unclassified	0	0	0	0	0	0	0	0	0	0	0	0	0	2	0	0	1	0	0

Copyright © 2020, Sociedade Brasileira de Genética

Level_1	Level_2	Level_3	Level_4	Level_5	Level_6	Level_7	ss10	sn15	sn16	ss19	sn20	ss21	ss25	sn26	sn27	ss28	ss2	ss30	sn31	sn35	sn36	sn5	ss6	ss8	sn9
k__Bacteria	p__Tenericutes	c__Mollicutes	o__RF39	Unclassified	Unclassified	Unclassified	0	0	0	0	0	0	0	0	0	0	0	0	0	0	3	0	0	0	0
k__Bacteria	Unclassified	Unclassified	Unclassified	Unclassified	Unclassified	Unclassified	0	0	0	0	0	0	0	0	0	0	0	0	0	0	5	0	0	0	0
k__Bacteria	p__Firmicutes	c__Clostridia	o__Clostridiales	f__Ruminococcaceae	Unclassified	Unclassified	0	0	0	0	0	0	0	0	0	0	0	0	0	0	51	0	0	0	0
k__Bacteria	p__Bacteroidetes	c__Bacteroidia	o__Bacteroidales	f__Porphyromonadaceae	g__Parabacteroides	Unclassified	0	0	0	0	0	0	0	0	0	0	0	0	0	0	2	0	0	0	0
k__Bacteria	p__Firmicutes	c__Clostridia	o__Clostridiales	f__Lachnospiraceae	Unclassified	Unclassified	0	0	0	0	0	0	0	0	0	0	0	0	0	0	6	0	0	0	0
k__Bacteria	Unclassified	Unclassified	Unclassified	Unclassified	Unclassified	Unclassified	0	0	0	0	0	0	0	0	0	0	0	0	0	0	7	0	0	0	0
k__Bacteria	p__Firmicutes	c__Clostridia	o__Clostridiales	f__Peptostreptococcaceae	Unclassified	Unclassified	0	0	0	0	0	0	0	0	0	0	0	0	0	0	1	0	1	2	0
k__Bacteria	p__Firmicutes	c__Clostridia	o__Clostridiales	Unclassified	Unclassified	Unclassified	0	0	0	0	0	0	0	0	0	0	0	0	0	0	3	0	0	1	0
k__Bacteria	Unclassified	Unclassified	Unclassified	Unclassified	Unclassified	Unclassified	0	0	0	0	0	0	0	0	0	0	0	0	0	0	2	0	1	0	0
k__Bacteria	p__Tenericutes	c__Mollicutes	o__RF39	Unclassified	Unclassified	Unclassified	0	0	0	0	0	0	0	0	0	0	0	0	0	0	1	0	3	2	0
k__Bacteria	p__Firmicutes	c__Clostridia	o__Clostridiales	Unclassified	Unclassified	Unclassified	0	0	0	0	0	0	0	0	0	0	0	0	0	0	1	1	1	4	0
k__Bacteria	p__Firmicutes	c__Clostridia	o__Clostridiales	f__Ruminococcaceae	g__Ruminococcus	Unclassified	0	0	0	0	0	0	0	0	0	0	0	0	0	0	0	86	0	0	0
k__Bacteria	p__Firmicutes	c__Clostridia	o__Clostridiales	Unclassified	Unclassified	Unclassified	0	0	0	0	0	0	0	0	0	0	0	0	0	0	0	2	0	0	0
k__Bacteria	Unclassified	Unclassified	Unclassified	Unclassified	Unclassified	Unclassified	0	0	0	0	0	0	0	0	0	0	0	0	0	0	0	1	0	3	4

Copyright © 2020, Sociedade Brasileira de Genética

**Level_1**	**Level_2**	**Level_3**	**Level_4**	**Level_5**	**Level_6**	**Level_7**	**ss10**	**sn15**	**sn16**	**ss19**	**sn20**	**ss21**	**ss25**	**sn26**	**sn27**	**ss28**	**ss2**	**ss30**	**sn31**	**sn35**	**sn36**	**sn5**	**ss6**	**ss8**	**sn9**
k__Bacteria	p__Bacteroidetes	c__Bacteroidia	o__Bacteroidales	f__[Paraprevotellaceae]	g__CF231	Unclassified	0	0	0	0	0	0	0	0	0	0	0	0	0	0	0	1	2	0	0
k__Bacteria	p__Firmicutes	c__Clostridia	o__Clostridiales	f__Peptostreptococcaceae	g__Peptostreptococcus	s__anaerobius	0	0	0	0	0	0	0	0	0	0	0	0	0	0	0	0	2	0	0
k__Bacteria	p__Firmicutes	c__Erysipelotrichi	o__Erysipelotrichales	f__Erysipelotrichaceae	Unclassified	Unclassified	0	0	0	0	0	0	0	0	0	0	0	0	0	0	0	0	5	2	0
k__Bacteria	Unclassified	Unclassified	Unclassified	Unclassified	Unclassified	Unclassified	0	0	0	0	0	0	0	0	0	0	0	0	0	0	0	0	24	18	2
k__Bacteria	p__Firmicutes	c__Clostridia	o__Clostridiales	Unclassified	Unclassified	Unclassified	0	0	0	0	0	0	0	0	0	0	0	0	0	0	0	0	19	3	0
k__Bacteria	p__Firmicutes	c__Clostridia	o__Clostridiales	f__Clostridiaceae	g__Clostridium	Unclassified	0	0	0	0	0	0	0	0	0	0	0	0	0	0	0	0	2	0	0
k__Bacteria	p__Actinobacteria	c__Coriobacteriia	o__Coriobacteriales	f__Coriobacteriaceae	g__Adlercreutzia	Unclassified	0	0	0	0	0	0	0	0	0	0	0	0	0	0	0	0	2	0	0
k__Bacteria	p__Bacteroidetes	c__Bacteroidia	o__Bacteroidales	f__S24-7	Unclassified	Unclassified	0	0	0	0	0	0	0	0	0	0	0	0	0	0	0	0	2	0	0
k__Bacteria	p__Firmicutes	c__Clostridia	o__Clostridiales	f__Lachnospiraceae	Unclassified	Unclassified	0	0	0	0	0	0	0	0	0	0	0	0	0	0	0	0	3	0	0
k__Bacteria	p__Firmicutes	c__Clostridia	o__Clostridiales	Unclassified	Unclassified	Unclassified	0	0	0	0	0	0	0	0	0	0	0	0	0	0	0	0	2	6	0
k__Bacteria	p__Bacteroidetes	c__Bacteroidia	o__Bacteroidales	f__S24-7	Unclassified	Unclassified	0	0	0	0	0	0	0	0	0	0	0	0	0	0	0	0	0	1484	0
k__Bacteria	p__Bacteroidetes	c__Bacteroidia	o__Bacteroidales	f__Prevotellaceae	g__Prevotella	Unclassified	0	0	0	0	0	0	0	0	0	0	0	0	0	0	0	0	0	169	2
k__Bacteria	p__Cyanobacteria	c__4C0d-2	o__YS2	Unclassified	Unclassified	Unclassified	0	0	0	0	0	0	0	0	0	0	0	0	0	0	0	0	0	36	0
k__Bacteria	p__Firmicutes	c__Clostridia	o__Clostridiales	f__Lachnospiraceae	Unclassified	Unclassified	0	0	0	0	0	0	0	0	0	0	0	0	0	0	0	0	0	23	0

Copyright © 2020, Sociedade Brasileira de Genética

**Level_1**	**Level_2**	**Level_3**	**Level_4**	**Level_5**	**Level_6**	**Level_7**	**ss10**	**sn15**	**sn16**	**ss19**	**sn20**	**ss21**	**ss25**	**sn26**	**sn27**	**ss28**	**ss2**	**ss30**	**sn31**	**sn35**	**sn36**	**sn5**	**ss6**	**ss8**	**sn9**
k__Bacteria	p__Tenericutes	c__Mollicutes	o__RF39	Unclassified	Unclassified	Unclassified	0	0	0	0	0	0	0	0	0	0	0	0	0	0	0	0	0	8	0
k__Bacteria	p__Bacteroidetes	c__Bacteroidia	o__Bacteroidales	f__Prevotellaceae	g__Prevotella	Unclassified	0	0	0	0	0	0	0	0	0	0	0	0	0	0	0	0	0	21	28
k__Bacteria	p__Bacteroidetes	c__Bacteroidia	o__Bacteroidales	f__Rikenellaceae	Unclassified	Unclassified	0	0	0	0	0	0	0	0	0	0	0	0	0	0	0	0	0	22	0
k__Bacteria	p__Firmicutes	Unclassified	Unclassified	Unclassified	Unclassified	Unclassified	0	0	0	0	0	0	0	0	0	0	0	0	0	0	0	0	0	19	0
k__Bacteria	p__Firmicutes	c__Clostridia	o__Clostridiales	f__Ruminococcaceae	Unclassified	Unclassified	0	0	0	0	0	0	0	0	0	0	0	0	0	0	0	0	0	22	0
k__Bacteria	p__Firmicutes	c__Clostridia	o__Clostridiales	Unclassified	Unclassified	Unclassified	0	0	0	0	0	0	0	0	0	0	0	0	0	0	0	0	0	13	0
k__Bacteria	Unclassified	Unclassified	Unclassified	Unclassified	Unclassified	Unclassified	0	0	0	0	0	0	0	0	0	0	0	0	0	0	0	0	0	6	0
k__Bacteria	p__Firmicutes	c__Clostridia	o__Clostridiales	Unclassified	Unclassified	Unclassified	0	0	0	0	0	0	0	0	0	0	0	0	0	0	0	0	0	2	7
k__Bacteria	p__Firmicutes	c__Clostridia	o__Clostridiales	Unclassified	Unclassified	Unclassified	0	0	0	0	0	0	0	0	0	0	0	0	0	0	0	0	0	17	0
k__Bacteria	p__Firmicutes	c__Clostridia	o__Clostridiales	f__Lachnospiraceae	Unclassified	Unclassified	0	0	0	0	0	0	0	0	0	0	0	0	0	0	0	0	0	2	3
k__Bacteria	Unclassified	Unclassified	Unclassified	Unclassified	Unclassified	Unclassified	0	0	0	0	0	0	0	0	0	0	0	0	0	0	0	0	0	2	0
k__Bacteria	p__Bacteroidetes	c__Bacteroidia	o__Bacteroidales	Unclassified	Unclassified	Unclassified	0	0	0	0	0	0	0	0	0	0	0	0	0	0	0	0	0	2	0
k__Bacteria	p__Proteobacteria	c__Alphaproteobacteria	o__RF32	Unclassified	Unclassified	Unclassified	0	0	0	0	0	0	0	0	0	0	0	0	0	0	0	0	0	0	38
k__Bacteria	p__Firmicutes	c__Clostridia	o__Clostridiales	Unclassified	Unclassified	Unclassified	0	0	0	0	0	0	0	0	0	0	0	0	0	0	0	0	0	0	9

Copyright © 2020, Sociedade Brasileira de Genética

Level_1	Level_2	Level_3	Level_4	Level_5	Level_6	Level_7	ss10	sn15	sn16	ss19	sn20	ss21	ss25	sn26	sn27	ss28	ss2	ss30	sn31	sn35	sn36	sn5	ss6	ss8	sn9
k__Bacteria	p__Firmicutes	c__Clostridia	o__Clostridiales	f__Lachnospiraceae	g__Coprococcus	Unclassified	0	0	0	0	0	0	0	0	0	0	0	0	0	0	0	0	0	0	8
k__Bacteria	p__Firmicutes	c__Clostridia	o__Clostridiales	Unclassified	Unclassified	Unclassified	0	0	0	0	0	0	0	0	0	0	0	0	0	0	0	0	0	0	5
k__Bacteria	Unclassified	Unclassified	Unclassified	Unclassified	Unclassified	Unclassified	0	0	0	0	0	0	0	0	0	0	0	0	0	0	0	0	0	0	5
k__Bacteria	p__Firmicutes	c__Clostridia	o__Clostridiales	Unclassified	Unclassified	Unclassified	0	0	0	0	0	0	0	0	0	0	0	0	0	0	0	0	0	0	4
k__Bacteria	p__Firmicutes	c__Clostridia	o__Clostridiales	Unclassified	Unclassified	Unclassified	0	0	0	0	0	0	0	0	0	0	0	0	0	0	0	0	0	0	3
