# Supplementary material for: Metabarcoding reveals that a non-nutritive sweetener and sucrose yield similar gut microbiota patterns in Wistar rats
Source: Genet Mol Biol. 2020 Mar 16;43(1):e20190028. doi: 10.1590/1678-4685-GMB-2019-0028 (PMC7197999; doi:10.1590/1678-4685-GMB-2019-0028)
Supplement: Supplementary file 2 [file 1415-4757-GMB-43-1-e20190028-s1.pdf]

Genetics and Molecular Biology 43, 1 (2020)
Copyright © 2020, Sociedade Brasileira de Genética.

Supplementary material to "Metabarcoding reveals that a non-nutritive sweetener and sucrose yield similar gut microbiota patterns in Wistar rats"

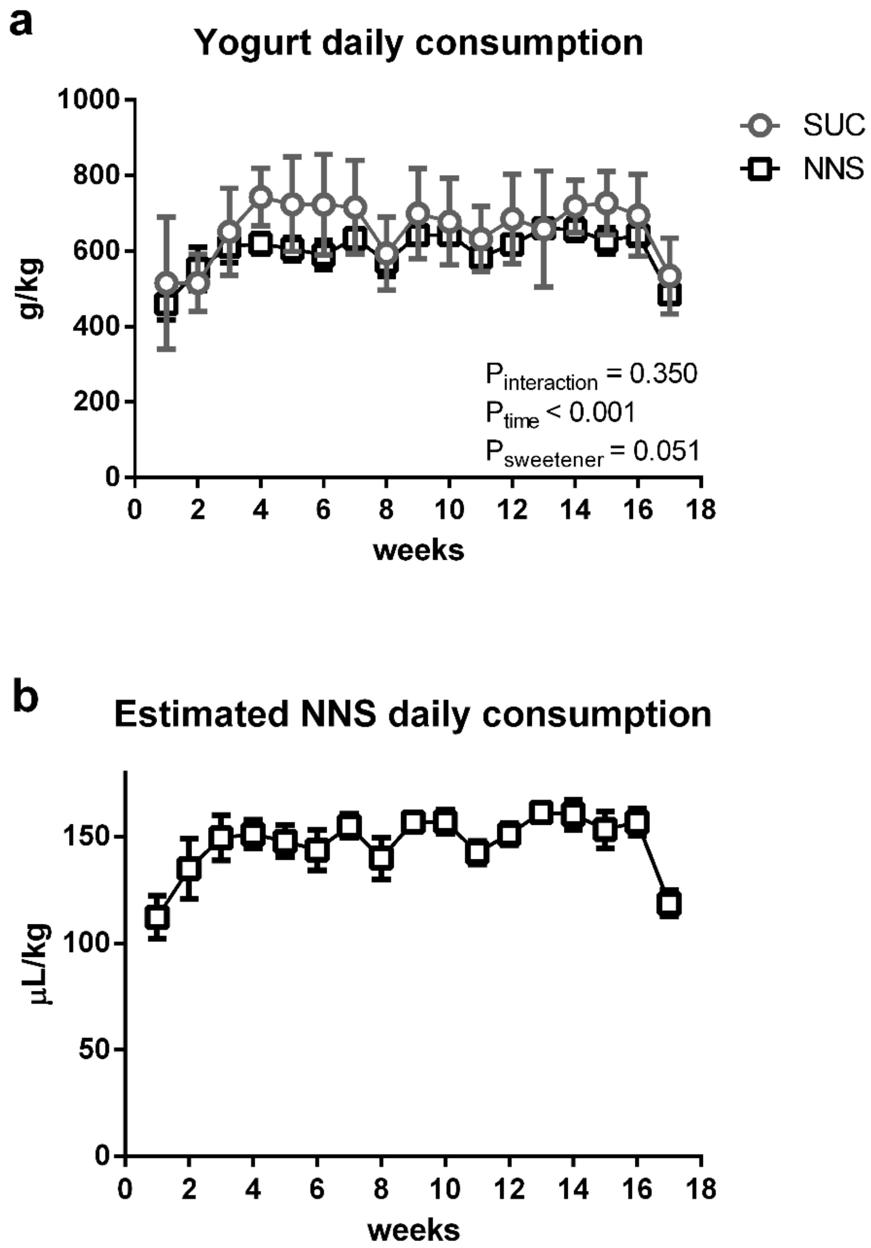

Figure S1 - Yogurt (a) and NNS intake (b) over time. Yogurt intake was evaluated weekly and corrected for body weight. NNS intake was estimated out of the final concentration of sweetener present in the yogurt solution (0.17%) and corrected for body weight. NNS, non-nutritive sweetener (n = 10); SUC, sucrose (n = 9)
