# Supplementary material for: Metabarcoding reveals that a non-nutritive sweetener and sucrose yield similar gut microbiota patterns in Wistar rats
Source: Genet Mol Biol. 2020 Mar 16;43(1):e20190028. doi: 10.1590/1678-4685-GMB-2019-0028 (PMC7197999; doi:10.1590/1678-4685-GMB-2019-0028)
Supplement: Supplementary file 3 [file 1415-4757-GMB-43-1-e20190028-s2.pdf]

Genetics and Molecular Biology 41, 1 (2020)
Copyright © 2020, Sociedade Brasileira de Genética.

# Supplementary material to "Metabarcoding reveals that a non-nutritive sweetener and sucrose yield similar gut microbiota patterns in Wistar rats"

**File S1** - Shannon and Simpson indices of diversity. The name of each sheet indicates the respective index.

**File S1 -** Shannon index of diversity.

| Sample | Shannon_diversity_index |
|---|---|
| ss10 | 3.538667822 |
| sn15 | 3.799902343 |
| sn16 | 4.340066193 |
| ss19 | 3.905047448 |
| sn20 | 3.410054225 |
| ss21 | 3.773343807 |
| ss25 | 3.710766156 |
| sn26 | 3.959871778 |
| sn27 | 4.055083982 |
| ss28 | 3.227322384 |
| ss2 | 3.545241855 |
| ss30 | 4.573818065 |
| sn31 | 4.014366528 |
| sn35 | 3.869647747 |
| sn36 | 3.803644328 |
| sn5 | 4.001957683 |
| ss6 | 4.063701449 |
| ss8 | 4.278634983 |
| sn9 | 4.177561885 |

**File S1 -** Simpson index of diversity.

| Sample | Simpson_diversity_index |
|---|---|
| ss10 | 0.923476728 |
| sn15 | 0.951974565 |
| sn16 | 0.969864542 |
| ss19 | 0.944695805 |
| sn20 | 0.907142672 |
| ss21 | 0.937280839 |
| ss25 | 0.935652157 |
| sn26 | 0.960596451 |
| sn27 | 0.954117589 |
| ss28 | 0.890961389 |
| ss2 | 0.902288008 |
| ss30 | 0.97520263 |
| sn31 | 0.960698678 |
| sn35 | 0.950667893 |
| sn36 | 0.94102147 |
| sn5 | 0.961838062 |
| ss6 | 0.955972074 |
| ss8 | 0.971913728 |
| sn9 | 0.968464731 |
