# Supplementary material for: Metabarcoding reveals that a non-nutritive sweetener and sucrose yield similar gut microbiota patterns in Wistar rats
Source: Genet Mol Biol. 2020 Mar 16;43(1):e20190028. doi: 10.1590/1678-4685-GMB-2019-0028 (PMC7197999; doi:10.1590/1678-4685-GMB-2019-0028)
Supplement: Supplementary file 4 [file 1415-4757-GMB-43-1-e20190028-s3.pdf]

Genetics and Molecular Biology 43, 1 (2020)
Copyright © 2020, Sociedade Brasileira de Genética.

Supplementary material to "Metabarcoding reveals that a non-nutritive sweetener and sucrose yield similar gut microbiota patterns in Wistar rats"

File S2 - One by One comparison of identified taxa (from Domain to Genus) results. Domain. Wilcoxon signed-rank test.

Domain	W value	P-value	FDR
k__Archaea	36	0.496698348	0.4966983
k__Bacteria	54	0.496698348	0.4966983

Copyright © 2020, Sociedade Brasileira de Genética.

File S2 - One by One comparison of identified taxa (from Domain to Genus) results. Plylum. T test.

Phylum	t value	p-value	FDR
p__Actinobacteria	0.393324731	0.6995125	1
p__Bacteroidetes	0.870564995	0.399137182	1
p__Cyanobacteria	0.710381211	0.487237874	1
p__Elusimicrobia	0.555856511	0.585581898	1
p__Euryarchaeota	0.47079656	0.645104591	1
p__Firmicutes	0.535536083	0.600671157	1
p__Proteobacteria	0.975918004	0.347487875	1
p__Spirochaetes	-0.279808357	0.783041979	1
p__Tenericutes	0.502372295	0.622113678	1

Genetics and Molecular Biology 43, 1 (2020)
Copyright © 2020, Sociedade Brasileira de Genética.

File S2 - One by One comparison of identified taxa (from Domain to Genus) results. Phylum. Wilcoxon signed-rank test.

Phylum	W value	p-value	FDR
p__Deferribacteres	43	0.904825824	1
p__Fibrobacteres	48	0.842105263	1
p__Fusobacteria	73	0.022018229	0.3522917
p__Lentisphaerae	46	0.968239191	1
p__TM7	45	1	1
p__Verrucomicrobia	21	0.053475936	0.4278075
p__WPS-2	47	0.904825824	1

Copyright © 2020, Sociedade Brasileira de Genética.

File S2 - One by One comparison of identified taxa (from Domain to Genus) results. Class. T test.

Class	t value	p-value	FDR
c__4C0d-2	0.48246616	0.635637431	0.9684211
c__Alphaproteobacteria	1.626967845	0.122854596	0.899305
c__Bacilli	-0.331900315	0.744021946	0.9684211
c__Bacteroidia	0.694425083	0.49852263	0.9684211
c__Betaproteobacteria	0.905656981	0.381354273	0.9684211
c__Clostridia	0.685592807	0.504518451	0.9684211
c__Deltaproteobacteria	0.701505489	0.49260911	0.9684211
c__Elusimicrobia	0.227214693	0.823314401	0.9684211
c__Epsilonproteobacteria	-0.355029539	0.72701749	0.9684211
c__Erysipelotrichi	1.00302387	0.33143404	0.9684211
c__Methanobacteria	1.670899969	0.113192528	0.899305
c__Mollicutes	0.137629392	0.892150734	0.9725558
c__Spirochaetes	-0.811924689	0.428071943	0.9684211
c__Thermoplasmata	-0.088810774	0.930270771	0.9725558

Copyright © 2020, Sociedade Brasileira de Genética.

File S2 - One by One comparison of identified taxa (from Domain to Genus) results. Class. Wilcoxon signed-rank test.

Class	W value	p-value	FDR
c__[Lentisphaeria]	39	0.660720085	0.9684211
c__Actinobacteria	49	0.780185759	0.9684211
c__Coriobacteriia	48	0.842105263	0.9684211
c__Deferribacteres	38	0.603780121	0.9684211
c__Fibrobacteria	49	0.780185759	0.9684211
c__Fusobacteriia	69	0.053475936	0.899305
c__Gammaproteobacteria	59	0.277511962	0.968421
c__TM7-3	45	1	1
c__Verruco-5	27	0.156400875	0.899305

Copyright © 2020, Sociedade Brasileira de Genética.

File S2 - One by One comparison of identified taxa (from Domain to Genus) results. Order. T test.

Order	t value	p-value	FDR
o__Burkholderiales	0.352714557	0.729105749	1
o__Campylobacterales	-0.790538091	0.440195517	1
o__Clostridiales	-0.040145729	0.968444785	1
o__Desulfovibrionales	0.034738667	0.97269503	1
o__E2	-0.732002	0.474914383	1
o__Elusimicrobiales	-0.249680143	0.806479648	1
o__Erysipelotrichales	0.408323809	0.688222539	1
o__Lactobacillales	-0.479530708	0.638500445	1
o__RF32	0.855817077	0.404168322	1
o__RF39	-0.401272345	0.694428807	1
o__Spirochaetales	-1.079514607	0.295958686	1
o__YS2	-0.017518661	0.986240281	1

Copyright © 2020, Sociedade Brasileira de Genética.

File S2 - One by One comparison of identified taxa (from Domain to Genus) results. Order. Wilcoxon signed-rank test.

Order	W value	p-value	FDR
o__Actinomycetales	44	0.968239191	1
o__Aeromonadales	57	0.356232003	1
o__Bacillales	56	0.400181861	1
o__Bacteroidales	44	0.968239191	1
o__Bifidobacteriales	45	1	1
o__Coriobacteriales	44	0.968239191	1
o__CW040	43	0.904825824	1
o__Deferribacterales	37	0.548961874	1
o__Enterobacteriales	47	0.904825824	1
o__Fibrobacterales	45	1	1
o__Fusobacteriales	64	0.133300136	1
o__Gemellales	41	0.780185759	1
o__M2PT2-76	29	0.21102427	1
o__Methanobacteriales	56	0.400181861	1
o__Pasteurellales	43	0.904825824	1
o__Rickettsiales	67	0.078893243	1
o__Turicibacterales	51	0.660720085	1
o__Victivallales	35	0.446967893	1
o__WCHB1-41	22	0.065253632	1

Copyright © 2020, Sociedade Brasileira de Genética.

File S2 - One by One comparison of identified taxa (from Domain to Genus) results. Family. T test.

Family	t value	p-value	FDR
f__[Methanomassiliicoccaceae]	-1.055730809	0.305873743	1
f__[Mogibacteriaceae]	-0.759577043	0.458028243	1
f__[Odoribacteraceae]	-0.517938102	0.611263458	1
f__[Paraprevotellaceae]	-0.212112836	0.834617762	1
f__Alcaligenaceae	0.327335068	0.748113948	1
f__Bacteroidaceae	-0.689862717	0.500286075	1
f__Clostridiaceae	-0.751614613	0.463818707	1
f__Desulfovibrionaceae	-0.057218338	0.95503844	1
f__Elusimicrobiaceae	-0.384404184	0.706912037	1
f__Erysipelotrichaceae	0.38842546	0.702976358	1
f__Helicobacteraceae	-1.017632937	0.323124231	1
f__Lachnospiraceae	-0.236307829	0.816054494	1
f__Lactobacillaceae	-0.633878904	0.536062989	1
f__Methanobacteriaceae	0.476780807	0.641410106	1
f__Peptococcaceae	0.456252589	0.654204024	1
f__Porphyromonadaceae	-0.393202804	0.699068963	1
f__Prevotellaceae	-0.012150808	0.990448923	1
f__Rikenellaceae	-0.131743352	0.896922452	1
f__Ruminococcaceae	-0.073447181	0.942308307	1
f__Spirochaetaceae	-1.250560839	0.228500465	1
f__Streptococcaceae	0.149741695	0.882984731	1
f__Veillonellaceae	-0.329176903	0.746325616	1

Copyright © 2020, Sociedade Brasileira de Genética.

File S2 - One by One comparison of identified taxa (from Domain to Genus) results. Family. Wilcoxon signed-rank test.

Family	W value	p-value	FDR
f__[Tissierellaceae]	51	0.660720085	1
f__Actinomycetaceae	70	0.043473554	1
f__Aerococcaceae	68	0.065253632	1
f__Bifidobacteriaceae	45	1	1
f__Christensenellaceae	40	0.719695166	1
f__Comamonadaceae	53	0.548961874	1
f__Coriobacteriaceae	45	1	1
f__Deferribacteraceae	37	0.548961874	1
f__Dehalobacteriaceae	31	0.277511962	1
f__Enterobacteriaceae	48	0.842105263	1
f__Eubacteriaceae	45	1	1
f__F16	44	0.968239191	1
f__Fibrobacteraceae	45	1	1
f__Fusobacteriaceae	66	0.094719522	1
f__Gemellaceae	42	0.842105263	1
f__Micrococcaceae	34	0.400181861	1
f__mitochondria	56	0.400181861	1
f__p-2534-18B5	46	0.968239191	1
f__Pasteurellaceae	46	0.968239191	1
f__Peptostreptococcaceae	36	0.496698348	1
f__RF16	36	0.496698348	1
f__RFP12	22	0.065253632	1
f__S24-7	46	0.968239191	1
f__Staphylococcaceae	53	0.548961874	1
f__Succinivibrionaceae	57	0.356232003	1
f__Turicibacteraceae	48	0.842105263	1
f__Victivallaceae	35	0.446967893	1

Genetics and Molecular Biology 43, 1 (2020)
Copyright © 2020, Sociedade Brasileira de Genética.

File S2 - One by One comparison of identified taxa (from Domain to Genus) results. Genus. T test.

Genus	t value	p-value	FDR
g__[Prevotella]	0.70399012	0.491645959	0.9682392
g__Allobaculum	0.200931977	0.843512948	0.9682392
g__Bacteroides	-0.696798624	0.49557718	0.9682392
g__Blautia	-0.63304706	0.538328282	0.9682392
g__Clostridium	1.249903546	0.22961	0.9682392
g__Coprococcus	-0.154580666	0.87903137	0.9682392
g__Desulfovibrio	-0.126196301	0.90106089	0.9682392
g__Dorea	-0.240558426	0.81279291	0.9682392
g__Lactobacillus	-0.640402696	0.533127185	0.9682392
g__Oscillospira	-0.08333813	0.934561034	0.9682392
g__p-75-a5	0.728673532	0.477570499	0.9682392
g__Parabacteroides	-0.298911673	0.768630971	0.9682392
g__Prevotella	0.218167694	0.829914469	0.9682392
g__rc4-4	1.27222699	0.220421465	0.9682392
g__Ruminococcus	-0.463793337	0.648729798	0.9682392
g__Streptococcus	0.339776979	0.739654373	0.9682392
g__Sutterella	0.569768206	0.576355444	0.9682392
g__Treponema	-1.167595573	0.259311687	0.9682392
g__vadinCA11	-0.802979226	0.433583955	0.9682392
g__YRC22	-0.070865961	0.944359496	0.9682392

Copyright © 2020, Sociedade Brasileira de Genética.

File S2 - One by One comparison of identified taxa (from Domain to Genus) results. Genus. Wilcoxon signed-rank test.

Genus	W value	p-value	FDR
g__[Eubacterium]	49	0.780185759	0.9682392
g__[Ruminococcus]	41	0.780185759	0.9682392
g__Actinomyces	62	0.182316136	0.9682392
g__Adlercreutzia	35	0.446967893	0.9682392
g__Aggregatibacter	44	0.968239191	0.9682392
g__Anaerobiospirillum	52	0.603780121	0.9682392
g__Anaerococcus	48	0.842105263	0.9682392
g__Anaerofustis	43	0.904825824	0.9682392
g__Anaerostipes	32	0.31537812	0.9682392
g__Bifidobacterium	42	0.842105263	0.9682392
g__Butyricicoccus	27	0.156400875	0.9682392
g__Butyricimonas	30	0.242806729	0.9682392
g__Candidatus Arthromitus	51	0.660720085	0.9682392
g__CF231	26	0.133300136	0.9682392
g__Collinsella	33	0.356232003	0.9682392
g__Coprobacillus	52	0.603780121	0.9682392
g__Dehalobacterium	24	0.094719522	0.9682392
g__Elusimicrobium	27	0.156400875	0.9682392
g__Escherichia	56	0.400181861	0.9682392
g__Facklamia	62	0.182316136	0.9682392
g__Faecalibacterium	57	0.356232003	0.9682392
g__Fibrobacter	46	0.968239191	0.9682392
g__Flexispira	27	0.156400875	0.9682392
g__Lachnospira	54	0.496698348	0.9682392
g__Methanobrevibacter	53	0.548961874	0.9682392
g__Methanosphaera	48	0.842105263	0.9682392
g__Mogibacterium	38	0.603780121	0.9682392
g__Mucispirillum	38	0.603780121	0.9682392
g__Odoribacter	35	0.446967893	0.9682392
g__Oribacterium	58	0.31537812	0.9682392
g__Paraprevotella	48	0.842105263	0.9682392
g__Peptostreptococcus	37	0.548961874	0.9682392
g__Phascolarctobacterium	44	0.968239191	0.9682392
g__RFN20	58	0.31537812	0.9682392
g__Roseburia	47	0.904825824	0.9682392

Copyright © 2020, Sociedade Brasileira de Genética.

File S2 - One by One comparison of identified taxa (from Domain to Genus) results. Genus. Wilcoxon signed-rank test.

Genus	W value	p-value	FDR
g__Rothia	39	0.660720085	0.9682392
g__Staphylococcus	52	0.603780121	0.9682392
g__Turicibacter	42	0.842105263	0.9682392
g__Veillonella	53	0.548961874	0.9682392
g__Victivallis	31	0.277511962	0.9682392
